# Asymmetrical Evolution of Promoter Methylation of Mammalian Genes after Duplication

**DOI:** 10.1093/molbev/msae259

**Published:** 2024-12-17

**Authors:** Mercedes de la Fuente, Isabel Mendizabal, Mira V Han, Soojin V Yi, David Alvarez-Ponce

**Affiliations:** Departamento de Ciencias y Técnicas Fisicoquímicas, Facultad de Ciencias, Universidad Nacional de Educación a Distancia (UNED), Madrid 28232, Spain; Center for Cooperative Research in Biosciences (CIC bioGUNE), Basque Research and Technology Alliance (BRTA), 48160 Derio, Spain; Ikerbasque, Basque Foundation for Science, Bilbao, Spain; Translational Prostate Cancer Research Lab, CIC bioGUNE-Basurto, Biocruces Bizkaia Health Research Institute, Derio, Spain; School of Life Sciences, University of Nevada Las Vegas, Las Vegas, NV, USA; Nevada Institute of Personalized Medicine, University of Nevada Las Vegas, Las Vegas, NV, USA; Department of Ecology, Evolution, and Marine Biology, University of California, Santa Barbara, CA, USA; Department of Molecular, Cellular and Developmental Biology, University of California, Santa Barbara, CA, USA; Neuroscience Research Institute, University of California, Santa Barbara, CA, USA; Biology Department, University of Nevada, Reno, NV, USA

**Keywords:** gene duplication, DNA methylation, cytosine methylation, paralogs, parental copy, daughter copy

## Abstract

Even though gene duplication is a key source of new genes and evolutionary innovation, it is unclear how duplicates survive the period immediately following gene duplication, in which both copies are functionally redundant. In the absence of epigenetic silencing, the abundance of the gene product would double after gene duplication, which would often have deleterious effects. However, recent duplicates exhibit low expression levels, which could be at least partially explained by high levels of promoter methylation. What evolutionary paths lead to duplicate hypermethylation, and does it affect both duplicates or only one? Here, we compare levels of promoter methylation in 10 human and 16 mouse tissues, between singletons and duplicates and among human–mouse orthologs of different kinds (one-to-one, one-to-many, many-to-one, and many-to-many). Our results indicate that: (i) on average, duplicates are more methylated than singletons in mouse, but less methylated than singletons in human, (ii) recently duplicated genes tend to exhibit high levels of promoter methylation, (iii) genes that undergo duplication tend to be highly methylated before duplication, (iv) after gene duplication, one of the copies (the daughter copy, i.e. the one that relocates to a new genomic context) tends to undergo an additional increase in promoter methylation, whereas the other (the parental copy, which remains in the original genomic location) tends to retain preduplication methylation levels, and (v) daughter copies tend to be lowly expressed. These observations support a model in which daughter copies are repressed via promoter hypermethylation and can thus survive the filter of purifying selection until both copies diverge functionally.

## Introduction

Gene duplication is the main source of new genes and a key source of evolutionary innovation (Ohno 1970; Zhang 2003). However, it is unclear how duplicates survive the steps immediately following gene duplication, in which they are functionally redundant, before they have the chance to functionally diverge (Hahn 2009). Indeed, most gene duplications result in pseudogenization of one of the copies (Lynch and Conery 2000). In the absence of silencing mechanisms, gene duplication is expected to lead to a doubling in the abundance of the mRNA and the encoded protein, which is expected to often be deleterious (e.g. due to disruption of the stoichiometric balance of protein–protein interactions and complexes; Papp et al. 2003; He and Zhang 2006; Birchler and Veitia 2007).

It has been shown that gene duplication is often compensated by a reduction in gene expression that reverts mRNA abundances back to preduplication levels, thus making gene duplication nearly neutral (Qian et al. 2010; Chang and Liao 2012). How are gene duplicates silenced? Modeling studies supported the possibility that gene repositioning and epigenetic silencing may enable persistence of duplicates (Rodin and Riggs 2003; Rodin et al. 2005). Subsequent studies in mammals (Chang and Liao 2012; Keller and Yi 2014) and fishes (Zhong et al. 2016; Huang and Chain 2021) have shown that young duplicates tend to be hypermethylated at their promoter regions (an epigenetic modification that often leads to gene silencing in vertebrates; Newell-Price et al. 2000). This effect fades over time and ends up not being present in older duplicates (Keller and Yi 2014; Zhong et al. 2016; Huang and Chain 2021). This has led to the hypothesis that increased promoter methylation allows recent duplicates to persist in the genome neutrally or nearly neutrally (i.e. with no or minimal deleterious effects) until they undergo functional divergence (subfunctionalization, neofunctionalization, or a combination of both) and their co-existence in the same genome does not result in stoichiometric imbalance.

However, the evolutionary path leading to the hypermethylation of duplicates is currently unknown. One possibility is that gene duplication preferentially affects genes that are highly methylated (i.e. duplicates are hypermethylated before gene duplication). Another possibility, which is not mutually exclusive with the first scenario, is that genes experience increased methylation after gene duplication. In this case, it is possible that both copies undergo hypermethylation, or that only one does.

In certain cases, gene duplication leads to 2 initially identical copies that remain in tandem in the original chromosomal location. In other cases, it leads to 2 distinguishable copies: the parental copy, which remains in the original location, and the daughter copy, which relocates to a different part of the chromosome or to another chromosome, thus facing a different genomic context. Our understanding of how these different genomic contexts influence the evolutionary paths of each duplicate is still limited (Holland et al. 2017). Nonetheless, it has been shown that daughter copies tend to undergo accelerated nonsynonymous substitutions, positive selection, and changes in gene expression, whereas the parental copy continues to evolve at the preduplication rates, presumably maintaining the ancestral function (Han et al. 2009; Pegueroles et al. 2013). We thus hypothesized that hypermethylation may affect only (or mostly) the daughter copy, with the parental copy retaining the preduplication methylation levels.

Here, we use whole-genome bisulfite sequencing (WGBS) data from 10 human and 16 mouse tissues to compare the levels of promoter methylation of different kinds of human–mouse orthologs (one-to-one, one-to-many, many-to-one, and many-to-many), with a special focus on those genes that duplicated after the divergence of primates and rodents (which took place ∼75 million years ago). We show that: (i) on average, duplicates are more methylated than singletons in mouse, but less methylated than singletons in human, (ii) recently duplicated genes (those that duplicated after the primates/rodents split) tend to exhibit higher levels of promoter methylation than those genes that did not duplicate in the same period of time, (iii) genes that undergo duplication tend to be highly methylated before duplication, (iv) after gene duplication, the daughter copy tends to undergo an additional increase in promoter methylation, whereas the parental copy tends to retain preduplication methylation levels, and (v) daughter copies tend to be lowly expressed compared to parental copies. These results deepen our understanding of the mechanisms enabling gene duplication.

## Results

### Singletons and Duplicates Differ in Their Promoter Methylation Levels

**Fig. 1.**
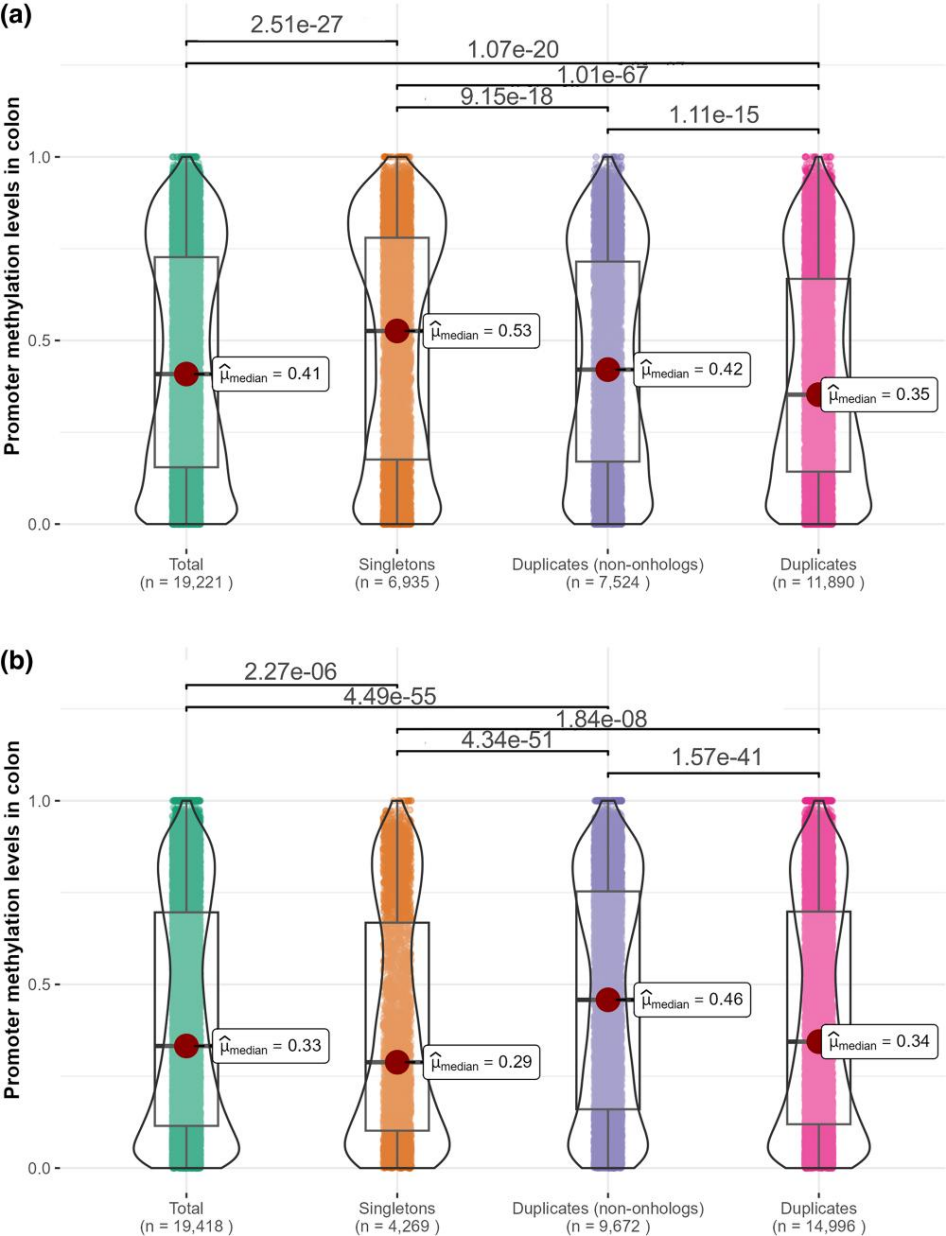
Comparison of colon promoter methylation levels in human and mouse singletons versus. duplicates. a) Human genes; b) mouse genes. Each dot represents a gene. Horizontal lines show significant differences (*P*-values < 0.05) according to Dunn's pairwise tests with Holm correction for multiple comparisons. Similar graphs for other tissues are shown in supplementary dataset S1, Supplementary Material online.

Out of the 68,016 human genes listed in the Ensembl database (Cunningham et al. 2022), 46,479 were classified as singletons and 21,540 were classified as duplicates. Out of these genes, 22,824 were protein-coding genes. For each gene, we obtained promoter methylation data from 10 human tissues (Mendizabal and Yi 2016). Throughout this manuscript, we highlight the results corresponding to colon, but results for the other 9 tissues are consistent and are presented in the Supplementary material (supplementary dataset S1, Supplementary Material online). Levels of promoter methylation in colon were available for 6,935 singleton and 11,890 duplicated genes. Methylation levels were significantly higher for singletons (median = 0.53) than for duplicates (median = 0.35, Mann-Whitney's *U* test, *P* = 2.68 × 10^−67^; Fig. 1a). The difference persisted when ohnologs (genes resulting from the 2 rounds of whole-genome duplication (WGD) that took place in a common ancestor of vertebrates; Holland et al. 1994; Dehal and Boore 2005) were removed from our set of duplicates (Fig. 1a).

Contrasting results were obtained in mouse. Out of 55,414 genes, 33,030 were classified as singletons and 22,384 as duplicates. Out of these genes, 21,885 were protein-coding genes. We obtained data from 16 mouse tissues (Hon et al. 2013). Levels of promoter methylation in colon were available for 4,269 singleton and 14,996 duplicated genes. Methylation levels were significantly higher for duplicates (median = 0.34) than for singletons (median = 0.29, *P* = 3.89 × 10^−9^; Fig. 1b). The difference persisted when ohnologs were removed from our analysis (Fig. 1b).

### Recent Duplicates Are Highly Methylated

**Fig. 2.**
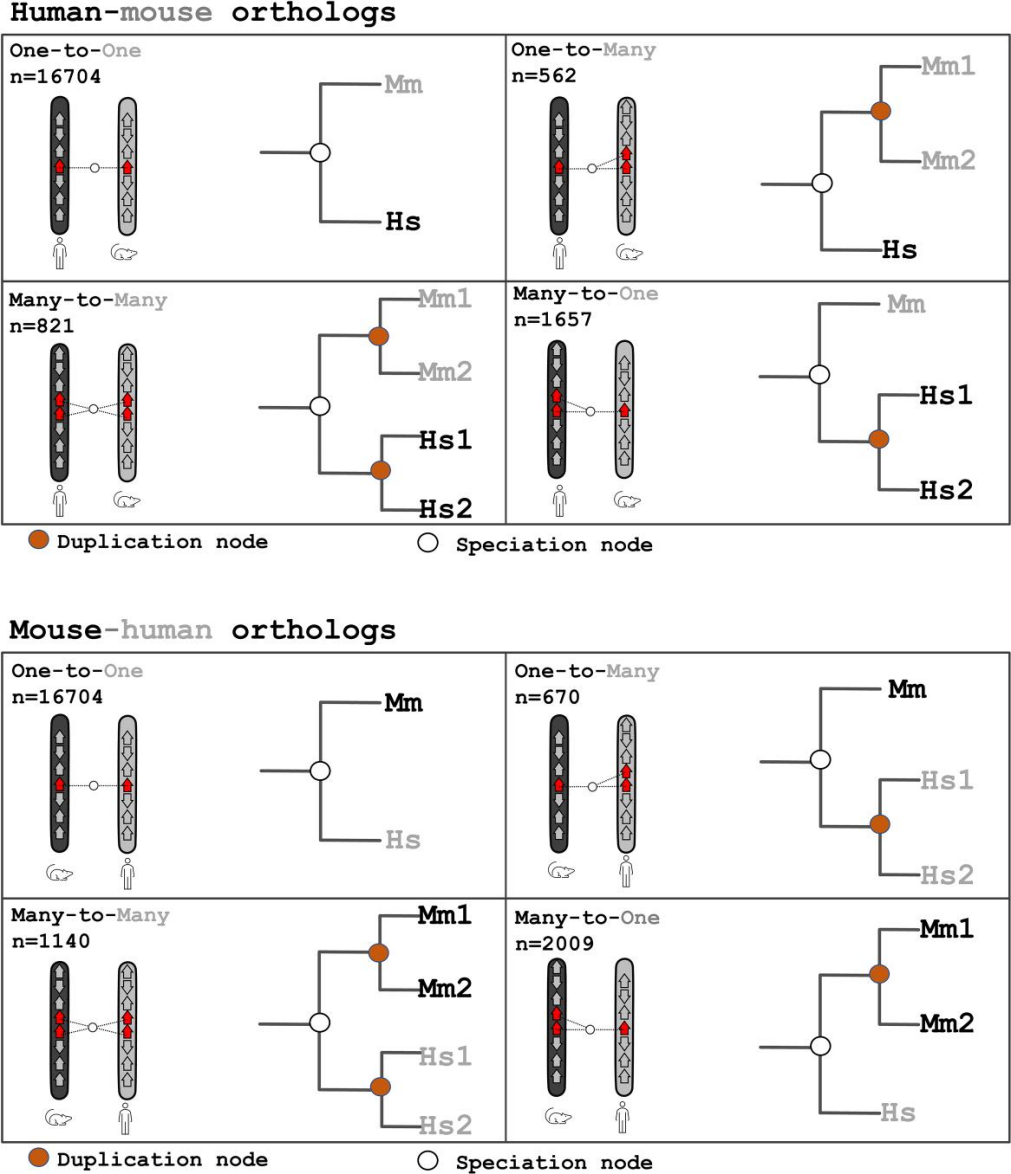
Diagrams representing the evolutionary relationships among different kinds of human–mouse orthologs.

For 19,744 human genes, we identified at least 1 ortholog in mouse. We classified these genes into 4 categories: one-to-one orthologs (1 ortholog in each species, *n* = 16,704), one-to-many orthologs (1 ortholog in human and 2 or more co-orthologs in mouse, *n* = 562), many-to-one orthologs (2 or more co-orthologs in human and 1 ortholog in mouse, *n* = 1,657), and many-to-many orthologs (2 or more co-orthologs in human and 2 or more co-orthologs in mouse due to independent duplication in both lineages, *n* = 821; Fig. 2a). Similarly, for 20,524 mouse genes, we identified at least 1 ortholog in human, and we classified these genes into one-to-one orthologs (1 ortholog in each species, *n* = 16,704), one-to-many orthologs (1 ortholog in mouse and 2 or more co-orthologs in human, *n* = 670), many-to-one orthologs (2 or more co-orthologs in mouse and 1 ortholog in human, *n* = 2,009), and many-to-many orthologs (2 or more co-orthologs in mouse and 2 or more co-orthologs in human, *n* = 1,140; Fig. 2b).

**Fig. 3.**
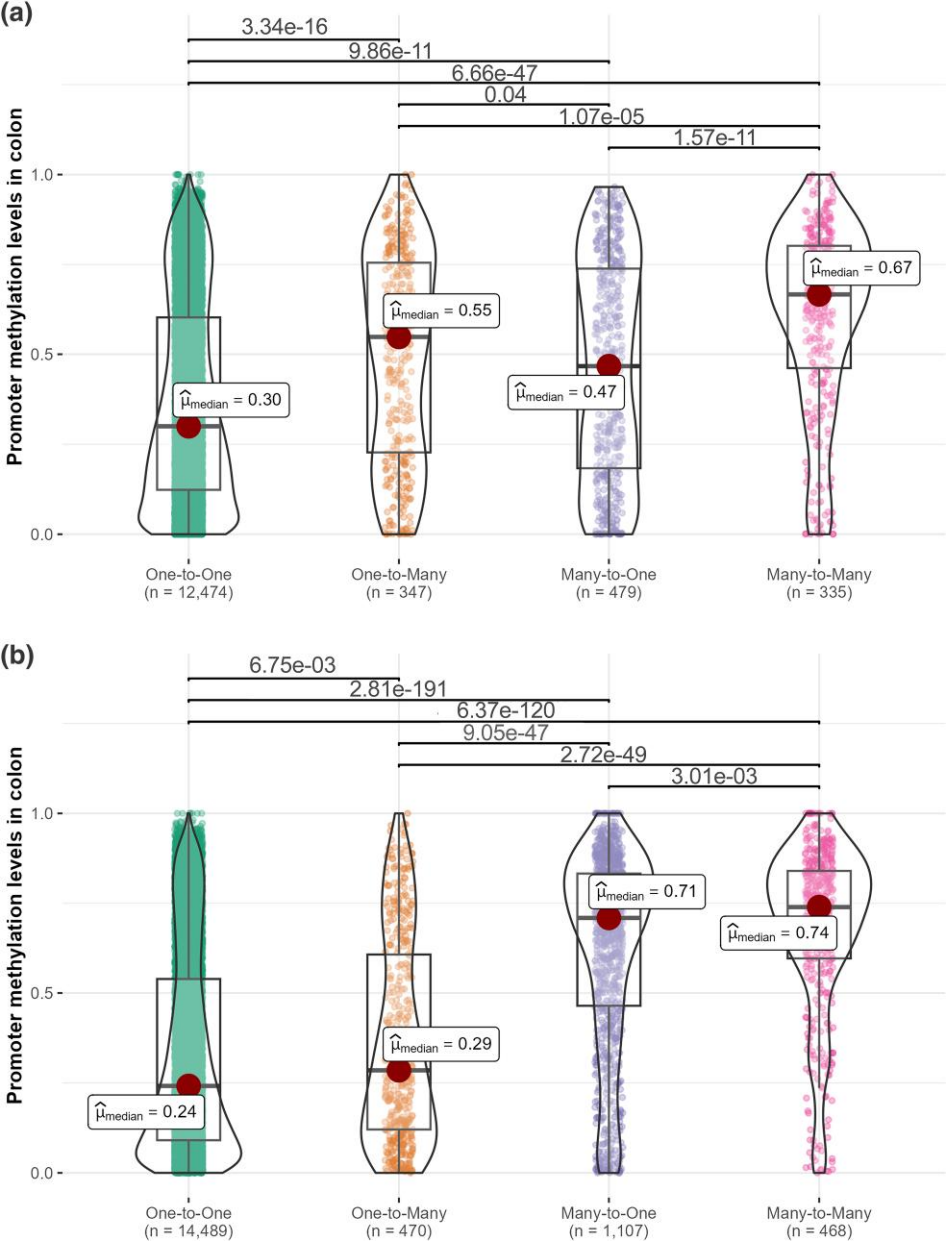
Comparison of colon promoter methylation levels among the different kinds of human–mouse orthologs. a) Human genes; b) mouse genes. Each dot represents a gene. Horizontal lines show significant differences (*P*-values < 0.05) according to Dunn's pairwise tests with Holm correction for multiple comparisons. Similar graphs for other tissues are shown in supplementary dataset S1, Supplementary Material online.

For both human and mouse, the many-to-one and the many-to-many orthologs represent genes that have duplicated after the divergence of human and mouse, which took place ∼75 million years ago. Conversely, the one-to-one and the one-to-many orthologs have not duplicated in the lineage of interest in the same time period. For both species, we found that promoter methylation levels were significantly higher in the many-to-one orthologs than in the one-to-one orthologs, and in the many-to-many orthologs than in the one-to-many orthologs (Fig. 3), indicating that recently duplicated genes (those that duplicated in the last ∼75 million years) were more methylated than those that did not methylate in the same time period.

### Promoter Methylation Levels Are High Prior to, and Further Increase After, Gene Duplication

We considered 2 alternative hypotheses that could account for the increased methylation levels of recently duplicated genes. First, these genes may have been highly methylated prior to duplication. Second, they may have experienced increased methylation after duplication. If the first scenario is correct, then we would expect the one-to-many orthologs in human (not duplicated in human but duplicated in mouse) and the one-to-many orthologs in mouse (not duplicated in mouse but duplicated in human) to be highly methylated.

In both species, one-to-many orthologs are more methylated than one-to-one orthologs. However, the difference is more substantial in humans, and in neither species the difference is large enough to explain the observed differences between one-to-one and many-to-one orthologs, or between one-to-many and many-to-many orthologs (Fig. 3). Indeed, among mouse nonduplicated genes, colon methylation levels are only 20% higher for one-to-many (median: 0.29) than for one-to-one orthologs (median: 0.24), while the differences are of 57% when comparing duplicated human many-to-one (median: 0.47) with nonduplicated human one-to-one orthologs (median: 0.30), and 22% when comparing human many-to-many (median: 0.67) with human one-to-many orthologs (median: 0.55). Similarly, among human nonduplicated genes, while methylation levels are 83% higher for one-to-many (median: 0.55) than for one-to-one orthologs (median = 0.30), the differences are much greater at 196% when comparing mouse duplicated many-to-one (median: 0.71) with mouse nonduplicated one-to-one orthologs (median: 0.24), and 155% when comparing mouse duplicated many-to-many (median: 0.74) with mouse nonduplicated one-to-many orthologs (median: 0.29; Fig. 3).

Taken together, these results support both hypotheses: First, duplicated genes are highly methylated before duplication (especially mouse duplicates, as evidenced by the substantial differences between human nonduplicated one-to-many and human un-duplicated one-to-one orthologs; Fig. 3). Second, duplicated genes undergo an increase in promoter methylation after duplication.

### Promoter Methylation Levels of Recent Duplicates Correlate with the Degree of Conserved Synteny

We considered whether promoter hypermethylation after gene duplication affected all gene copies equally, or whether certain gene copies were more affected than others. After gene duplication, 1 copy (known as the “parental copy”) may remain in the original genomic location, whereas the other (the “daughter copy”) relocates to a different part of the chromosome or to another chromosome (alternatively, both copies can end up in tandem in the original location). One can distinguish both copies from the size of the syntenic region around them, which is expected to be larger for the parental copy (Han and Hahn 2009; Han et al. 2009).

**Fig. 4.**
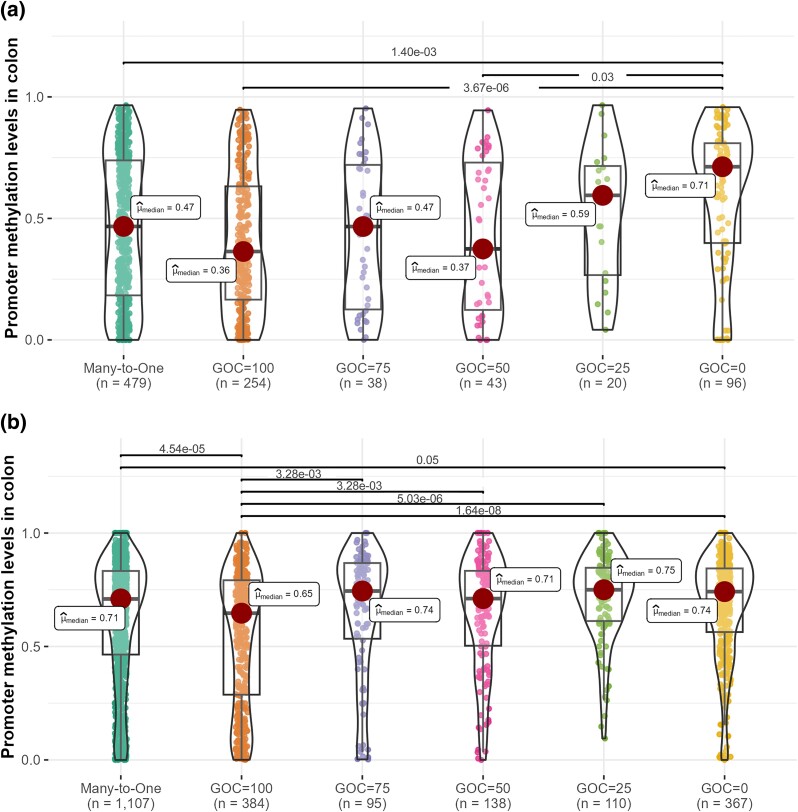
Comparison of colon promoter methylation levels among many-to-one genes with different GOC scores. a) Human genes; b) mouse genes. Each dot represents a gene. Horizontal lines show significant differences (*P*-values < 0.05) according to Dunn's pairwise tests with Holm correction for multiple comparisons. Similar graphs for other tissues are shown in supplementary dataset S1, Supplementary Material online.

For each human gene in a many-to-one group (i.e. genes duplicated in human but not in mouse in the last ∼75 million years), we obtained their gene order conservation (GOC) score from Ensembl (Cunningham et al. 2022). This synteny metric indicates how many of a gene's 4 closest neighbors (the 2 genes immediately upstream and the 2 genes immediately downstream) match between orthologous pairs (i.e. they are in the same order in the orthologous genomic region). The score can take 1 of 5 values: 100% if all 4 genes match, 75% if 3 of the genes match, 50% if 2 of the genes match, 25% if only 1 of the genes match, and 0% if none of the genes match (supplementary fig. S1, Supplementary Material online). Parental copies are expected to have higher GOC scores than daughter copies. Of note, most duplicates exhibited GOC values of 0% or 100% (Fig. 4). In general, we found that promoter methylation levels were low for human gene copies with GOC = 100%, and that they increased as GOC decreased (Fig. 4a). Indeed, promoter methylation levels negatively correlated with GOC scores (supplementary table S1, Supplementary Material online). Although not as pronounced, we obtained similar results when we classified mouse many-to-one orthologs (i.e. genes duplicated in mouse but not in human in the last ∼75 million years) according to their GOC scores (Fig. 4b; supplementary table S1, Supplementary Material online). These results suggest a scenario in which daughter copies underwent hypermethylation after gene duplication.

### The Promoter Methylation Increase following Gene Duplication Preferentially Affects Daughter Copies

Results presented in Fig. 4 suggest that, on average, daughter duplicates tend to undergo promoter hypermethylation after gene duplication. Parental copies, on the other hand, may (i) not experience an increase in levels of methylation, or (ii) not to the same extent. To distinguish between both possibilities, we examined many-to-one groups of genes (i.e. genes duplicated in 1 species but not in the other) in further detail.

For those groups that included more than 2 duplicates from the same species, only the 2 copies with the highest difference in promoter methylation levels were retained. Furthermore, groups in which all duplicates exhibit the same GOC score were removed. We then used the GOC scores to distinguish between the most likely parental copy (that with the highest GOC score) and the most likely daughter copy (that with the lowest GOC score).

**Fig. 5.**
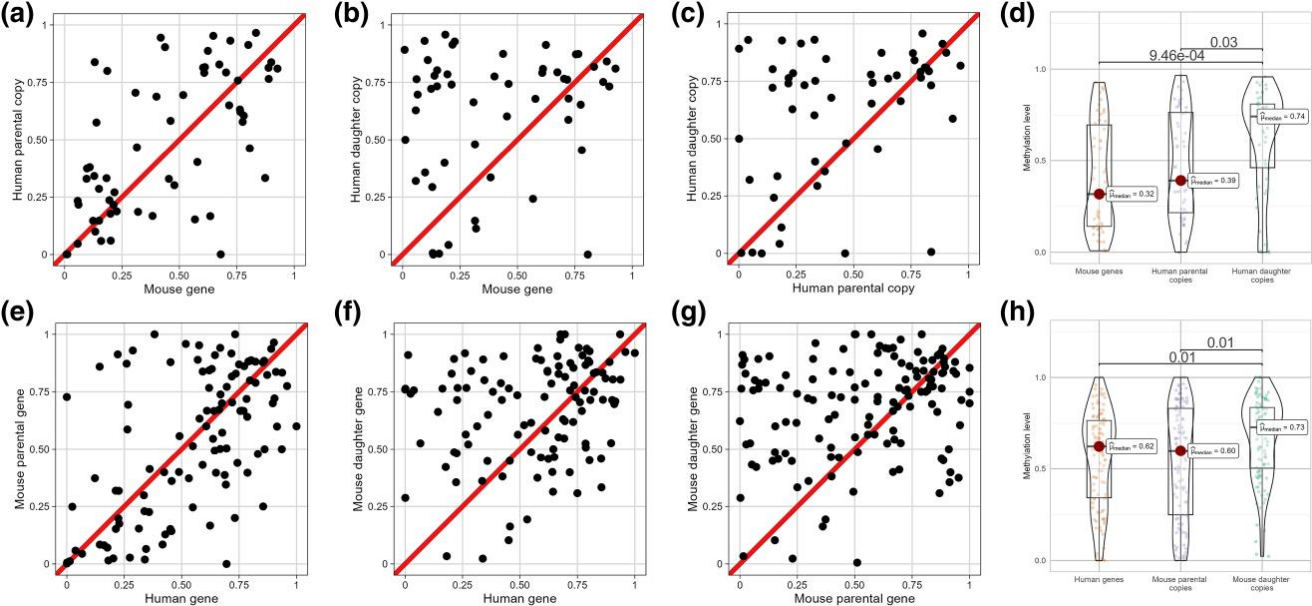
Comparison of colon promoter methylation levels in trios including 2 copies in human (parental and daughter) and 1 in mouse a–d), and trios including 2 copies in mouse (parental and daughter) and 1 in human e–h). Each dot represents a pair of genes within a trio. For each graph, the diagonal line corresponds to an equal amount of methylation in both genes. Similar graphs for other tissues are shown in supplementary dataset S1, Supplementary Material online.

We identified 50 trios including 2 copies in human and only 1 copy in mouse (i.e. genes that duplicated in human but not in mouse in the last ∼75 million years). Colon promoter methylation levels were higher in the human daughter copy more often (in 32 trios) than in the human parental copy (18 trios; one-tailed binomial test, *P* = 0.033; Fig. 5c). Significant differences were also observed in 8 out of the 10 studied human tissues (supplementary table S2, Supplementary Material online). For colon, liver, and placenta (the only 3 tissues for which data are available for both human and mouse), we also compared the levels of methylation of both human copies with those of the mouse gene. Human daughter copies were significantly hypermethylated in all 3 tissues, whereas human parental copies were only hypermethylated in placenta (Fig. 5a and b;  supplementary table S3, Supplementary Material online).

Similarly, we identified 135 to 142 trios that included duplicates in mouse but not in human (i.e. genes that duplicated in mouse but not in human in the last ∼75 million years). Again, colon promoter methylation levels were higher in the daughter copy more often (86 groups) than in the parental copy (49 groups; *P* = 0.0009; Fig. 5g). Significant differences were also observed in all 16 studied mouse tissues (supplementary table S4, Supplementary Material online). For colon, liver, and placenta, we compared the levels of methylation of both mouse copies with those of the human gene. Mouse daughter copies were significantly hypermethylated in colon and liver, whereas mouse parental copies were not hypermethylated in any of the tissues (Fig. 5e and f;  supplementary table S5, Supplementary Material online). A selection of representative trios is illustrated in dataset S2.

Taken together, these results indicate that, after gene duplication, parental copies tend to retain the promoter methylation levels of the ancestral (preduplication) gene (which we infer from the single copy in the other species), whereas daughter copies experience an increase in their promoter methylation levels.

**Fig. 6.**
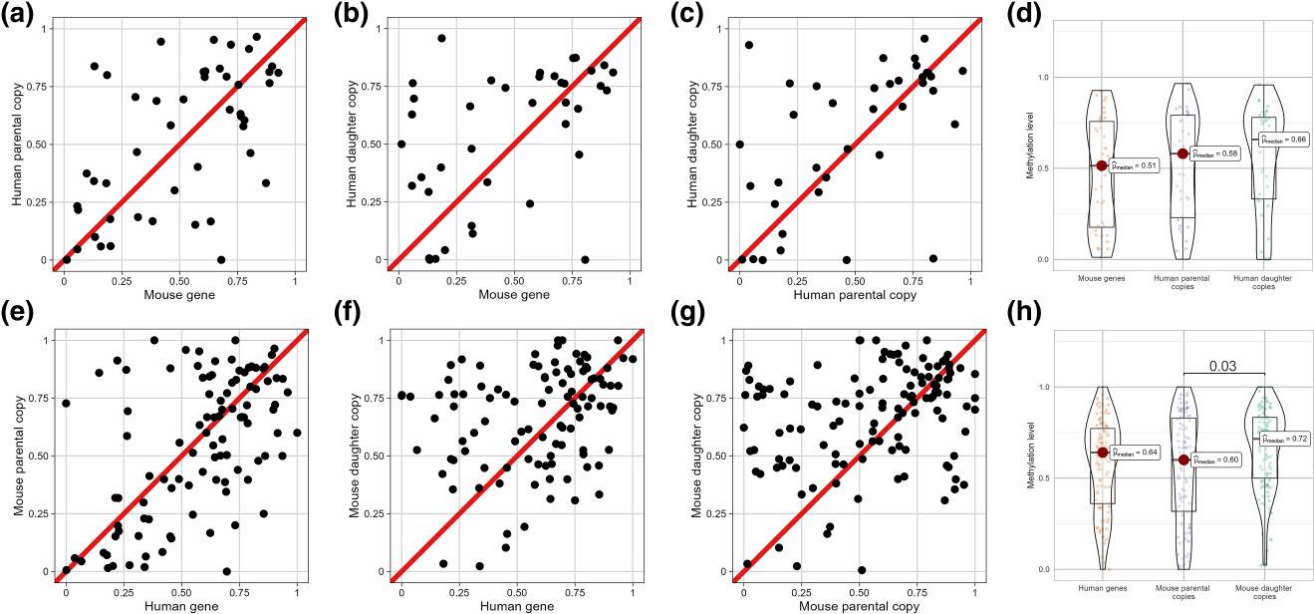
Comparison of colon promoter methylation levels in trios including 2 copies in human (parental and daughter) and 1 in mouse a–d), and trios including 2 copies in mouse (parental and daughter) and 1 in human e–h), excluding retrogenes. Each dot represents a pair of genes within a trio. For each graph, the diagonal line corresponds to an equal amount of methylation in both genes. Similar graphs for other tissues are shown in supplementary dataset S1, Supplementary Material online.

Retrogenes are always daughter gene copies, since they tend to insert in genomic regions located far away from the parental copy, and tend to be highly methylated (Keller and Yi 2014). This raises the possibility that they may be biasing our observations (driving the results presented on Fig. 5). To discard this possibility, we repeated our analyses after removing putative retrogenes. Removing such genes resulted in only 36 trios with 2 copies in human and 1 in mouse, and in 125 to 132 trios with 2 copies in mouse and 1 in human. Analysis of the 36 trios with 2 copies in human and 1 in mouse probably suffered from very limited power. Indeed, we could not find significant differences between human parental and daughter copies, neither in colon (Fig. 6a-d) nor in the other studied human tissues (with the only exception of embryonic stem cells, where daughter human copies were hypermethylated in 24 out of the 36 trios; one-tailed binomial test, *P* = 0.033; supplementary table S2, Supplementary Material online). Significant hypermethylation of human parental and daughter copies compared with their mouse orthologs was observed in placenta, but not in colon or liver (Fig. 6a and b;  supplementary table S3, Supplementary Material online). On the other hand, analysis of the 125 to 132 trios with 2 copies in mouse and 1 in human revealed significant differences between mouse parental and daughter copies in 9 of the 16 studied mouse tissues, including colon (Fig. 6g;  supplementary table S4, Supplementary Material online). Significant differences between mouse daughter copies and their human orthologs were observed in colon and liver (Fig. 6f;  supplementary table S5, Supplementary Material online), and no significant differences between mouse parental copies and their human orthologs were observed in colon, liver, or placenta (Fig. 6f;  supplementary table S5, Supplementary Material online). Taken together, these results indicate that daughter copies undergo hypermethylation after gene duplication, even if they are not retrogenes.

### Gene Body Methylation Generally Does not Follow the Same Trends as Promoter Methylation

While promoter methylation in mammals often results in silencing of gene expression, that is not the case of gene body methylation—on the contrary, gene body methylation is often associated with actively transcribed genes (Jjingo et al. 2012; Greenberg and Bourc’his 2019). Therefore, we did not expect to observe the same trends in both kinds of methylation.

We repeated all our analyses using levels of gene body methylation instead of promoter methylation. In general, we either did not observe the same trends, or we observed similar trends, but much less pronounced. Differences in methylation levels of singletons and duplicates were not always significant (supplementary dataset S3, Supplementary Material online, Section 1), and recent duplicates were often not hypermethylated (supplementary dataset S3, Supplementary Material online, Section 2). Even though levels of gene body methylation are higher for recent duplicates with less preserved synteny in human, the effect is much less pronounced than in promoters, and not observed in mouse (supplementary dataset S3, Supplementary Material online, Section 3). Finally, levels of gene body methylation are not generally higher for daughter copies than for parental copies (supplementary tables S6 to S9, Supplementary Material online, supplementary dataset S3, Supplementary Material online, Sections 4 and 5).

### Daughter Copies Tend to Exhibit a Reduced CpG Content

Methylated CpG dinucleotides tend to mutate to TpG dinucleotides. As a result, increased methylation levels often result in a reduced number of CpG dinucleotides. In fact, Chang and Liao (2012) found that the promoters of human and mouse recent duplicates were depleted in CpG dinucleotides. We thus hypothesized that daughter duplicated copies would often exhibit a lower CpG content than parental copies, since they tend to be highly methylated. To test this hypothesis, for each duplicate we computed its promoter's CpG_o/e_ (i.e. the observed number of CpG dinucleotides divided by the expected number of CpG dinucleotides given the promoter's nucleotide composition; Matsuo et al. 1993, Yi and Goodisman 2009). Among many-to-one orthologs, CpG_o/e_ values positively correlated with GOC scores in both human (ρ = 0.105, *P* = 0.016) and mouse (ρ = 0.191, *P* = 4.35 × 10^−11^; supplementary dataset S4, Supplementary Material online, Section 1). Out of 52 trios that duplicated in human but not in mouse, 33 exhibit lower CpG_o/e_ values in the daughter copy than in the parental copy (one-tailed binomial test, *P* = 0.035). Out of 148 trios that duplicated in mouse but not in human, 97 exhibit lower CpG_o/e_ values in the daughter copy than in the parental copy (one-tailed binomial test, *P* = 9.72 × 10^−5^), and the difference remains significant when retrogenes are removed from the analysis (*P* = 0.0016; supplementary table S10, Supplementary Material online). Taken together, these results indicate that the increased promoter methylation of recent daughter copies has reduced their CpG content, even though duplications took place in the last ∼75 million years.

### Daughter Copies Tend to be Lowly Expressed

**Fig. 7.**
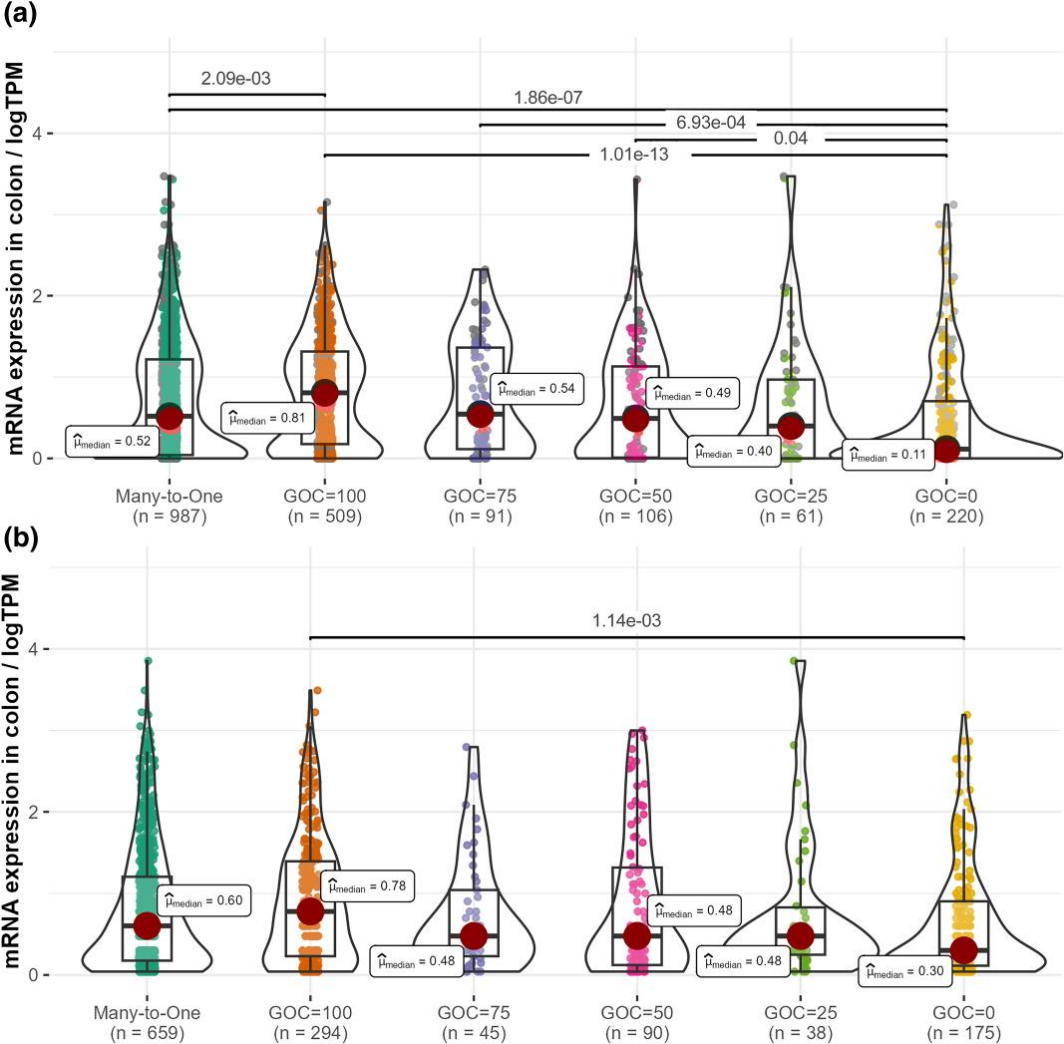
Comparison of colon mRNA abundance levels among many-to-one genes with different GOC scores. a) Human genes; b) mouse genes. Each dot represents a gene. Horizontal lines show significant differences (*P*-values < 0.05) according to Dunn's pairwise tests with Holm correction for multiple comparisons. Similar graphs for other tissues are shown in supplementary dataset S4, Supplementary Material online.

High levels of promoter methylation are generally associated with gene silencing in vertebrates; however, the link between promoter methylation and gene expression is complex (e.g. Newell-Price et al. 2000; de Mendoza et al. 2022). We thus tested whether daughter copies, in addition to exhibiting low levels of promoter methylation, were also lowly expressed compared with parental copies. To that end, we analyzed mRNA abundance data from 5 human and 5 mouse tissues (Merkin et al. 2012; Uhlén et al. 2015). Both in human and mouse, among many-to-one orthologs, mRNA abundances positively correlated with GOC scores (supplementary table S11, Supplementary Material online; Fig. 7; supplementary dataset S4, Supplementary Material online, Section 2). In addition, the number of trios in which daughter copies were less expressed significantly exceeded the number of trios in which parental copies were less expressed (supplementary tables S12 and S13, Supplementary Material online). After removing retrogenes, results remained significant in 3 of the 5 human tissues (supplementary table S12, Supplementary Material online) and in all 5 mouse tissues (supplementary table S13, Supplementary Material online). Taken together, these results indicate that the increased promoter methylation of daughter copies results in reduced levels of gene expression.

## Discussion

Our results indicate that young duplicates in the human and mouse genomes (those that duplicated in the last ∼75 million years) are generally highly methylated at their promoter regions, as evidenced by the observation that methylation levels are higher for many-to-one than for one-to-one orthologs, and for many-to-many than for one-to-many orthologs (Fig. 3; supplementary dataset S1, Supplementary Material online, Section 2). This pattern is consistent with previous observations in mammals and fishes that recent duplicates tend to be highly methylated (Chang and Liao 2012; Keller and Yi 2014; Zhong et al. 2016; Huang and Chain 2021). In addition, we show that recently duplicated genes are generally highly methylated before duplication, as evidenced by the fact that they tend to have highly methylated nonduplicated orthologs in the other species (methylation levels are higher in one-to-many than in one-to-one orthologs, and in many-to-many than in many-to-one orthologs; Fig. 3; supplementary dataset S1, Supplementary Material online, Section 2). Finally, analysis of genes that duplicated in human but not in mouse, or in mouse but not in human, reveals that: (i) recent duplicates with a high degree of synteny conservation (expected in parental copies) tend to be less methylated than those with a low degree of synteny conservation (expected in daughter copies; Fig. 4; supplementary dataset S1, Supplementary Material online, Section 3); (ii) after duplication, the methylation levels of parental copies tend to be similar to those of the nonduplicated orthologs in the other species (indicating that parental copies retain preduplication methylation levels), whereas daughter copies tend to be more methylated than parental copies and the nonduplicated orthologs in the other species (indicating that daughter copies experience an increase in their methylation levels; Figs. 5 and 6; supplementary dataset S1, Supplementary Material online, Sections 4 and 5); and (iii) consistent with their high levels of promoter methylation, daughter copies tend to be lowly expressed compared with parental copies (Fig. 7; supplementary dataset S4, Supplementary Material online, Section 2).

These results are compatible with the epigenetic complementation model (Rodin and Riggs 2003; Rodin et al. 2005), but they also expand it, offering us a more detailed picture of how gene duplications can survive the filter of purifying selection. In the absence of silencing mechanisms, gene duplication would be expected to lead to a doubling in total mRNA and protein abundances, which is expected to be often deleterious (Papp et al. 2003; He and Zhang 2006; Birchler and Veitia 2007). Nonetheless, thanks to silencing of the daughter copy via promoter methylation, total mRNA and protein abundances may remain similar to preduplication abundances. In this scenario, the duplication would be neutral (or nearly neutral), which would allow the daughter copy to survive until it diverges functionally from the parental copy. It is possible that promoter hypermethylation subsequently accelerates promoter sequence evolution and thus expression divergence of the daughter copy by increasing the rate of mutation via spontaneous deamination, as suggested by Huang and Chain (2021). In line with this model, we found that the promoters of daughter copies often exhibit a reduced number of CpG dinucleotides compared to parental copies (supplementary table S10, Supplementary Material online, supplementary dataset S4, Supplementary Material online, Section 1).

Why are duplicates often highly methylated before duplication? It is possible that gene duplication affects all genes with the same likelihood, but those that are highly methylated—and thus generally lowly expressed—are more likely to persist after gene duplication. Another question is: how do daughter copies undergo promoter hypermethylation? One possibility is that daughter copies reposition to random genomic regions after gene duplication, but are only likely to survive when they encounter silencing epigenetic contexts. Indeed, transfection experiments have shown that patterns of gene expression highly depend on the genomic location in which genes are inserted (e.g. Aladjem et al. 1998).

Some of our observations differ from those reported in previous studies. First, Chang and Liao (2012) inferred that DNA methylation occurred primarily after gene duplication, whereas Huang and Chain (2021) inferred that it occurred primarily before gene duplication. Our results support the validity of both models: gene duplication preferentially affects genes that are highly methylated at the promoter regions, and one of the copies undergoes additional promoter methylation after duplication. Second, previous works have inferred increased promoter methylation and/or reduced expression in both duplicated copies rather than in only one (Qian et al. 2010; Chang and Liao 2012; Huang and Chain 2021). If that is the case, then both copies would be expected to maintain their function; otherwise, total activity would be reduced compared with preduplication levels, which would often have deleterious effects (Qian et al. 2010). It should be noted, however, that previous studies focusing on the methylation of duplicates did not distinguish between parental and daughter copies beyond analyzing retrogenes separately.

Unlike small-scale duplication (SSD) events, WGD events do not upset the stoichiometric balance of protein–protein interactions and complexes, given that all genes in the genome are duplicated simultaneously (Veitia 2004, 2005). Therefore, ohnologs (genes resulting from WGD) can be maintained without experiencing the challenges discussed thus far, and are thus not expected to be highly methylated before methylation or to undergo additional methylation after duplication. Indeed, Keller and Yi (2014) found low levels of promoter methylation among ohnologs, a trend that we also observed (Fig. 1; supplementary dataset S1, Supplementary Material online, Section 1). By focusing on genes that duplicated in the human and/or mouse lineage in the last 75 million years, we restricted our analyses to duplicates resulting from SSD and completely excluded ohnologs (resulting from the 2 rounds of WGD that affected an ancestor of vertebrates over 450 million years ago; Holland et al. 1994; Dehal and Boore 2005). The only exception is our comparison of singleton versus duplicated genes (Fig. 1; supplementary dataset S1, Supplementary Material online, Section 1, supplementary dataset S3, Supplementary Material online, Section 1), in which duplicates of all ages (including ohnologs) were included in the same category. Nonetheless, removing ohnologs from this analysis did not alter our results (Fig. 1; supplementary dataset S1, Supplementary Material online, Section 1, supplementary dataset S3, Supplementary Material online, Section 1).

Our analyses have some limitations. First, a subset of our analyses requires availability of WGBS data from the same tissues in both human and mouse, and thus we had to restrict these analyses to colon, liver, and placenta (Figs. 5 and 6; supplementary dataset S1, Supplementary Material online, Sections 4 and 5). Second, in the case of placenta, our observations were somewhat complicated by generally higher methylation levels in human than in mouse (supplementary dataset S1, Supplementary Material online, Sections 4 and 5). This effect could be due to the fact that placenta methylation levels increase during gestation (Novakovic et al. 2011), and gestation is longer in human than in mouse. Indeed, patterns of gene expression in the mouse placenta change in parallel to those experienced in the first half of human placental development (Soncin et al. 2018). In addition, placenta is a complex organ, with some tissues derived from the mother and others (most of them) derived from the embryo (Caruso et al. 2012; Panja and Paria 2021). Third, a known problem when studying the patterns of methylation of recent duplicates is cross-mapping of some sequencing reads with both gene copies. Even though this problem is relatively uncommon when using WGBS (the current gold standard method to study cytosine methylation; Teh et al. 2016), we cannot completely rule out the possibility of some cross-mapping affecting our dataset. If present, this artifact is expected to artificially homogenize the methylation levels of some duplicates. Nonetheless, despite this potential problem, we found significant differences between the patterns of promoter methylation of parental and daughter copies. If anything, these limitations make our results more conservative (i.e. differences between gene copies might be more substantial than we have observed).

In general, our analyses of gene body methylation do not follow the same trends as those of promoter methylation (supplementary dataset S3, Supplementary Material online). This is expected since, unlike promoter methylation, gene body methylation often associates with actively transcribed genes, and its function remains not completely understood (Greenberg and Bourc’his 2019). However, in some of the tissues, some of the trends observed for promoter methylation are also observed for gene body methylation, albeit with much less intensity. This may be due to the positive correlation between promoter and gene body methylation (supplementary table S14, Supplementary Material online).

Gene silencing can take place through mechanism other than promoter methylation. Chang and Liao (2012) found no evidence that recent duplicates in human or mouse were silenced through microRNAs or changes in cis-regulatory sequence; thus, it is unlikely that these mechanisms contribute to the observed silencing of daughter copies. However, other silencing mechanisms, such as enhancer modification or polycomb-mediated repression (Blackledge and Klose 2021; Panigrahi and O’Malley 2021), cannot be discarded. Nevertheless, such mechanisms are not known to be associated with reduced CpG contents, which we also observed.

Thus far, relatively few studies have focused on the differences in the patterns of evolution of parental versus daughter copies after gene duplication, which has limited our understanding of the evolutionary processes underlying the functional divergence of duplicates (Holland et al. 2017). This is in part due to the difficulties associated with differentiating parental from daughter copies (Han and Hahn 2009; Holland et al. 2017). Our work illustrates the suitability of GOC scores to tell both copies apart (Fig. 4; supplementary fig. S1, Supplementary Material online, supplementary dataset S1, Supplementary Material online, Section 3). A gene's GOC score is relatively easy to compute and, even though it only focuses on the immediate vicinity of the gene (the 2 genes immediately upstream and the 2 genes immediately downstream of it), they seem to provide enough resolution to discriminate between parental and daughter copies.

Our results expand our understanding of how parental and daughter copies evolve asymmetrically after gene duplication: whereas parental copies tend to remain similar to the ancestral (preduplication) copy in terms of rates of evolution, expression patterns and promoter methylation, daughter copies tend to undergo accelerated nonsynonymous evolution, positive selection, changes in gene expression and increased promoter methylation (Han et al. 2009; Pegueroles et al. 2013; and our study).

## Methods

### Gene Classification

We classified each human and mouse gene (including protein-coding and nonprotein coding genes) into singletons or duplicates, and into different kinds of orthologs (one-to-one, one-to-many, many-to-one, many-to-many; Fig. 2) using the BioMart interface of Ensembl, version 106 (Cunningham et al. 2022). Duplicates were deemed as ohnologs if they were classified as such in the OHNOLOGS database, v2 (“intermediate” criteria; Singh and Isambert 2020).

For genes that duplicated after the divergence of human and mouse (many-to-one and many-to-many), we retrieved the GOC score from Ensembl. For many-to-one groups of orthologs (genes that duplicated in 1 species but not in the other) with more than 2 copies in human or mouse, the 2 copies with the highest difference in promoter methylation levels were retained, resulting in gene trios (2 copies in 1 species and 1 in the other). Within each trio, the copy with the highest GOC score was deemed to be the parental copy, and the 1 with the lowest GOC score was deemed to be the daughter copy. All trios included protein-coding genes. Recently, duplicated genes were deemed retrogenes if they had a single exon and their paralog had 3 or more exons (as in Lan and Pritchard 2016).

### Cytosine Methylation Data

We obtained the mean CpG fractional methylation values for promoters and gene bodies from published WGBS data from 10 human tissues (colon, liver, placenta, B cells, embryonic stem cells, hair, neurons, adrenal gland, ovary, and sperm; compiled by Mendizabal and Yi 2016) and 16 mouse tissues (colon, liver, placenta, cerebellum, cortex, heart, intestine, kidney, lung, olfactory bulb, pancreas, skin, spleen, stomach, thymus, and uterus; Hon et al. 2013). For each gene, promoter methylation levels (ranging from 0 to 1) were computed as the average of the methylation levels of all cytosines in the region comprised between 1,500 base pairs upstream of the transcription start site (TSS) and the TSS-1 position, and gene body methylation levels (also ranging from 0 to 1) were computed as the average of the methylation levels of the cytosines in the remainder of the gene region. Gene coordinates were accessed from Bioconductor packages TxDb.Hsapiens.UCSC.hg38.knownGene and TxDb.Mmusculus.UCSC.mm9.knownGene. For each gene, the longest transcript was considered. Overlapping genes were removed.

Human CpG dinucleotides were only retained if they were covered by at least 5 reads in all 10 tissues. Positions showing C/T polymorphisms with a minor allele frequency higher than 1% in the 1,000 Genomes Project (1000 Genomes Project Consortium et al. 2015) were removed. Mouse CpG dinucleotides were only retained if they were covered by 3 or more reads in each tissue. Positions displaying C/T polymorphisms according to the UCSC Table Browser were removed.

### CpG_o/e_ Calculations

For each gene with available methylation data, we computed its promoter's CpG_o/e_ as the observed frequency of CpG dinucleotides divided by the expected frequency of CpG dinucleotides (i.e. the frequency of C nucleotides multiplied by the frequency of G nucleotides).

### Gene Expression Data

We obtained mRNA abundance data from 5 human tissues (adrenal gland, colon, liver, ovary, and placenta) from the Human Protein Atlas, version 23.0 (RNA expression consensus dataset; Uhlén et al. 2015), and from 5 mouse tissues (colon, kidney, liver, lung, and spleen) from Expression Atlas (dataset: E-MTAB-2801, strain: C57Bl/6; Merkin et al. 2012). These tissues were a subset of the ones used in our methylation analyses.

### Statistical Analysis

R version 4.4.0. was used for most statistical analyses. Violin plots were generated using the ggstatsplot package version 0.12.3 (Patil 2021).

## Supplementary Material

msae259_Supplementary_Data

## Data Availability

All data used in this work are publicly available, as described in the Methods section.
