# Supplementary material for: Asymmetrical Evolution of Promoter Methylation of Mammalian Genes after Duplication
Source: Mol Biol Evol. 2024 Dec 17;41(12):msae259. doi: 10.1093/molbev/msae259 (PMC11683416; doi:10.1093/molbev/msae259)

Supplementary Materials – Dataset S1: Promoter methylation analyses

CONTENTS

1. Comparison of promoter methylation levels among singletons and duplicates ... 2

1.1. Human genes ... 2

1.2. Mouse genes ... 3

2. Comparison of promoter methylation levels among the different kinds of orthologs ... 6

2.1. Human genes ... 6

2.2. Mouse genes ... 7

3. Correlation of promoter methylation levels of recent duplicates with the Gene Order Conservation (GOC) scores ... 10

3.1. Human genes ... 10

3.2. Mouse genes ... 13

4. Promoter methylation in trios duplicated in human but not in mouse ... **19**

4.1. Comparison of promoter methylation of human daughter copies, human parental copies, and mouse orthologs ... 19

4.2. Comparison of promoter methylation of human daughter copies, human parental copies, and mouse orthologs: violin plots ... 19

4.3. Comparison of promoter methylation of human daughter copies, human parental copies, and mouse orthologs without retrogenes ... 20

4.4. Comparison of promoter methylation of human daughter copies, human parental copies, and mouse orthologs without retrogenes: violin plots ... 20

4.5. Comparison of promoter methylation of human daughter vs. human parental copies ... 21

4.6. Comparison of promoter methylation of human daughter vs. human parental copies: violin plots .. 21

4.7. Comparison of promoter methylation of human daughter copies, human parental copies, and mouse orthologs without retrogenes ... 22

4.8. Comparison of promoter methylation of human daughter copies, human parental copies, and mouse orthologs without retrogenes: violin plots ... 22

5. Promoter methylation in trios duplicated in mouse but not in human ... 23

5.1. Comparison of promoter methylation of mouse daughter copies, mouse parental copies, and human orthologs ... 23

5.2. Comparison of promoter methylation of mouse daughter copies, mouse parental copies, and human orthologs: violin plots ... 23

5.3. Comparison of promoter methylation of mouse daughter copies, mouse parental copies, and human orthologs without retrogenes ... 24

5.4. Comparison of promoter methylation of mouse daughter copies, mouse parental copies, and human orthologs without retrogenes: violin plots ... 24

5.5. Comparison of promoter methylation of mouse daughter vs. mouse parental copies ... 25

5.6. Comparison of promoter methylation of mouse daughter vs. mouse parental copies: violin plots .. 26

5.7. Comparison of promoter methylation of mouse daughter vs. mouse parental copies without retrogenes ... 27

5.8. Comparison of promoter methylation of mouse daughter vs. mouse parental copies without retrogenes: violin plots ... 28

1. Comparison of promoter methylation levels among singletons and duplicates

1.1. Human genes

Below are the representations of the comparison of promoter methylation levels in singletons vs. duplicates in the 10 human tissues analyzed. In each plot, each dot represents a gene. The horizontal lines indicate significant differences based on two-sided Dunn's pairwise tests with Holm correction for multiple comparisons. Comparisons were deemed significant if corrected p-values were lower than 0.05.

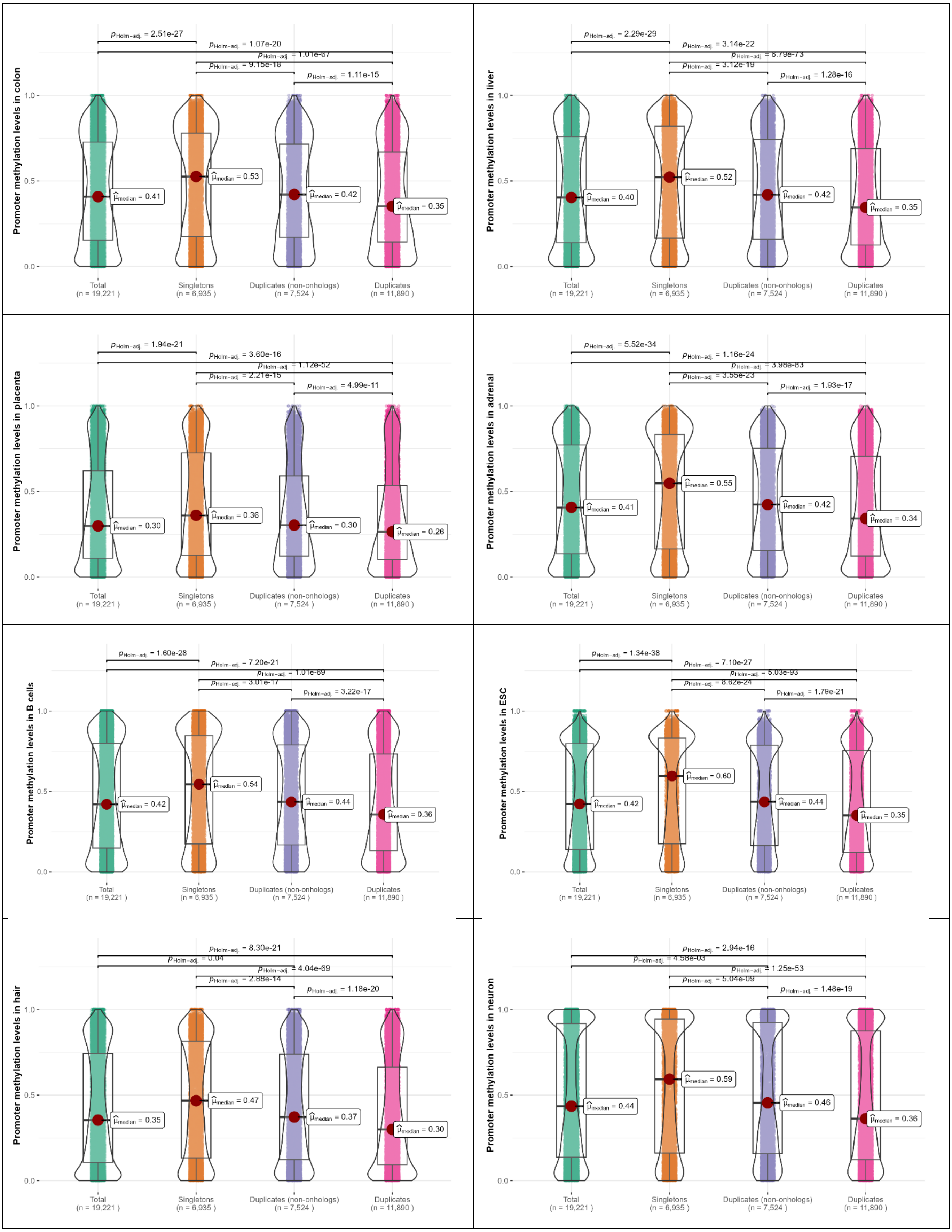

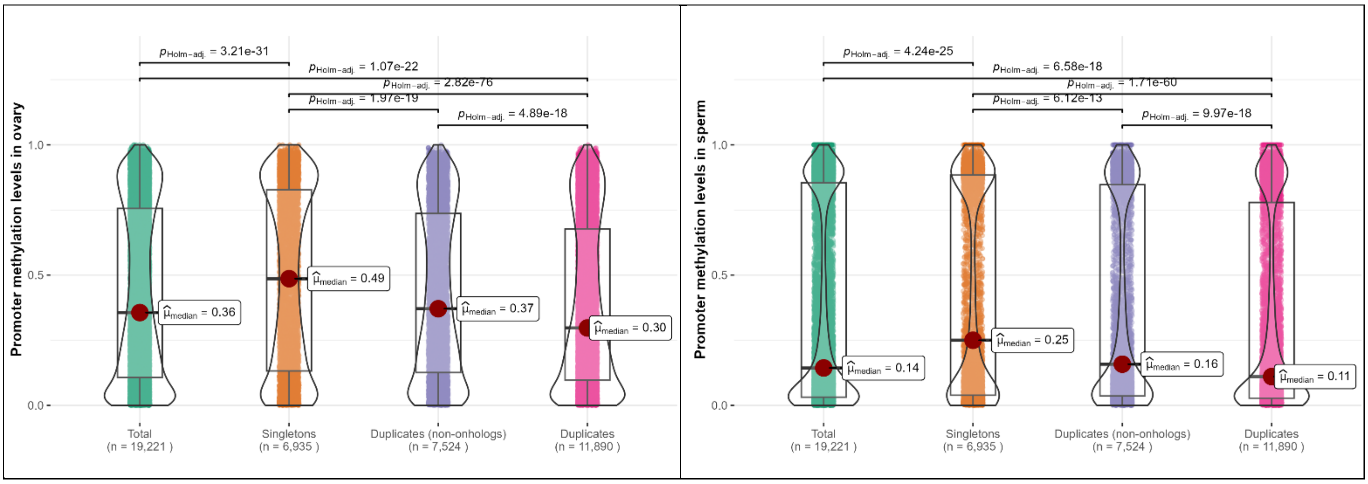

1.2. Mouse genes

Below are the representations of the comparison of promoter methylation levels in singletons vs. duplicates in the 16 mouse tissues analyzed. In each plot, each dot represents a gene. The horizontal lines indicate significant differences based on two-sided Dunn's pairwise tests with Holm correction for multiple comparisons. Comparisons were deemed significant if corrected p-values were lower than 0.05.

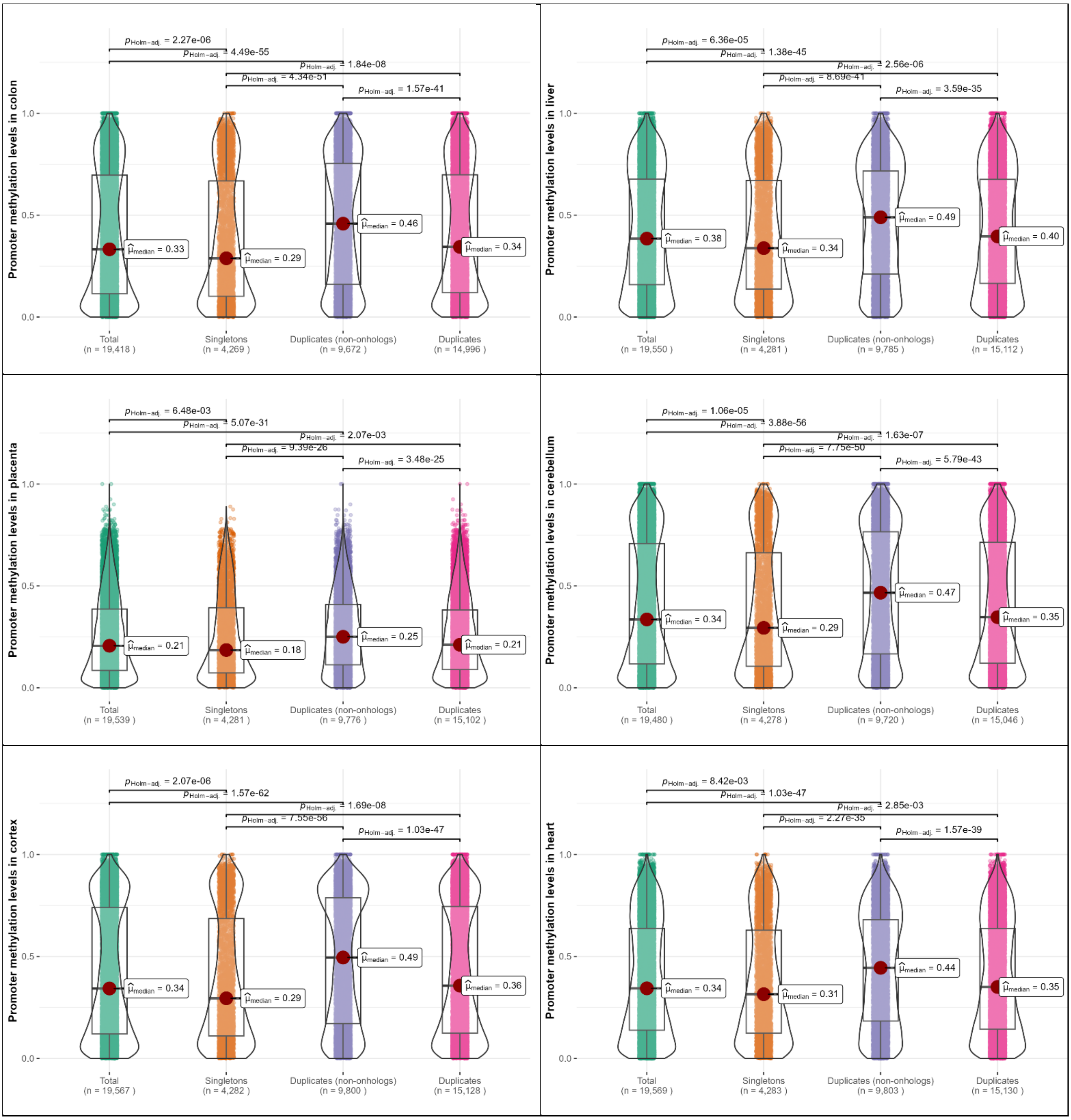

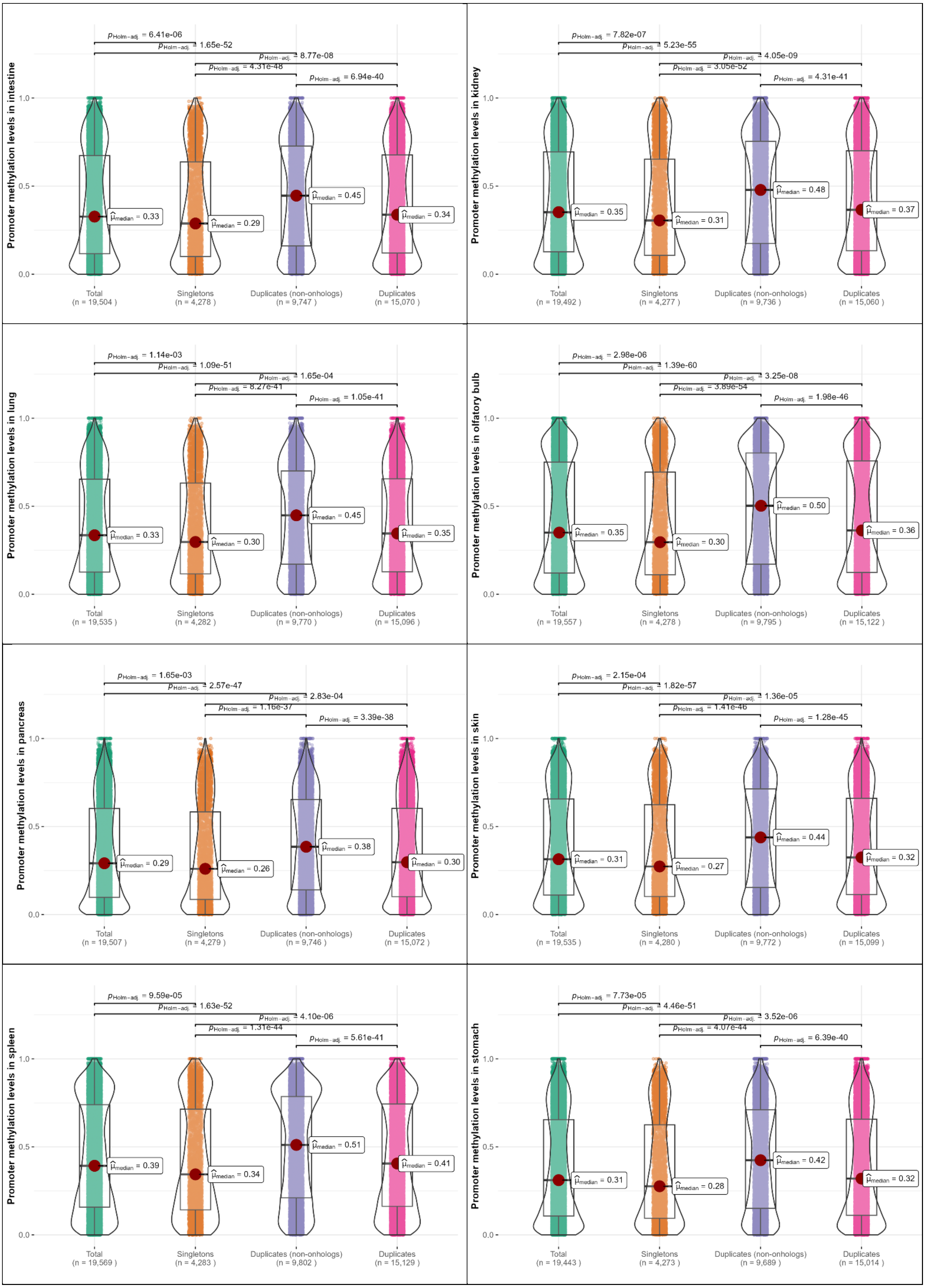

Promoter methylation levels in intestine
pHolm-adj. = 6.41e-06
pHolm-adj. = 1.65e-52
pHolm-adj. = 8.77e-08
pHolm-adj. = 4.31e-48
pHolm-adj. = 6.94e-40
μ̂median = 0.33
μ̂median = 0.29
μ̂median = 0.45
μ̂median = 0.34
Total (n = 19,504)
Singletons (n = 4,278)
Duplicates (non-onhologs) (n = 9,747)
Duplicates (n = 15,070)
Promoter methylation levels in kidney
pHolm-adj. = 7.82e-07
pHolm-adj. = 5.23e-55
pHolm-adj. = 4.05e-09
pHolm-adj. = 3.05e-52
pHolm-adj. = 4.31e-41
μ̂median = 0.35
μ̂median = 0.31
μ̂median = 0.48
μ̂median = 0.37
Total (n = 19,492)
Singletons (n = 4,277)
Duplicates (non-onhologs) (n = 9,736)
Duplicates (n = 15,060)
Promoter methylation levels in lung
pHolm-adj. = 1.14e-03
pHolm-adj. = 1.09e-51
pHolm-adj. = 1.65e-04
pHolm-adj. = 8.27e-41
pHolm-adj. = 1.05e-41
μ̂median = 0.33
μ̂median = 0.30
μ̂median = 0.45
μ̂median = 0.35
Total (n = 19,535)
Singletons (n = 4,282)
Duplicates (non-onhologs) (n = 9,770)
Duplicates (n = 15,096)
Promoter methylation levels in olfatory bulb
pHolm-adj. = 2.98e-06
pHolm-adj. = 1.39e-60
pHolm-adj. = 3.25e-08
pHolm-adj. = 3.89e-54
pHolm-adj. = 1.98e-46
μ̂median = 0.35
μ̂median = 0.30
μ̂median = 0.50
μ̂median = 0.36
Total (n = 19,557)
Singletons (n = 4,278)
Duplicates (non-onhologs) (n = 9,795)
Duplicates (n = 15,122)
Promoter methylation levels in pancreas
pHolm-adj. = 1.65e-03
pHolm-adj. = 2.57e-47
pHolm-adj. = 2.83e-04
pHolm-adj. = 1.16e-37
pHolm-adj. = 3.39e-38
μ̂median = 0.29
μ̂median = 0.26
μ̂median = 0.38
μ̂median = 0.30
Total (n = 19,507)
Singletons (n = 4,279)
Duplicates (non-onhologs) (n = 9,746)
Duplicates (n = 15,072)
Promoter methylation levels in skin
pHolm-adj. = 2.15e-04
pHolm-adj. = 1.82e-57
pHolm-adj. = 1.36e-05
pHolm-adj. = 1.41e-46
pHolm-adj. = 1.28e-45
μ̂median = 0.31
μ̂median = 0.27
μ̂median = 0.44
μ̂median = 0.32
Total (n = 19,535)
Singletons (n = 4,280)
Duplicates (non-onhologs) (n = 9,772)
Duplicates (n = 15,099)
Promoter methylation levels in spleen
pHolm-adj. = 9.59e-05
pHolm-adj. = 1.63e-52
pHolm-adj. = 4.10e-06
pHolm-adj. = 1.31e-44
pHolm-adj. = 5.61e-41
μ̂median = 0.39
μ̂median = 0.34
μ̂median = 0.51
μ̂median = 0.41
Total (n = 19,569)
Singletons (n = 4,283)
Duplicates (non-onhologs) (n = 9,802)
Duplicates (n = 15,129)
Promoter methylation levels in stomach
pHolm-adj. = 7.73e-05
pHolm-adj. = 4.46e-51
pHolm-adj. = 3.52e-06
pHolm-adj. = 4.07e-44
pHolm-adj. = 6.39e-40
μ̂median = 0.31
μ̂median = 0.28
μ̂median = 0.42
μ̂median = 0.32
Total (n = 19,443)
Singletons (n = 4,273)
Duplicates (non-onhologs) (n = 9,689)
Duplicates (n = 15,014)

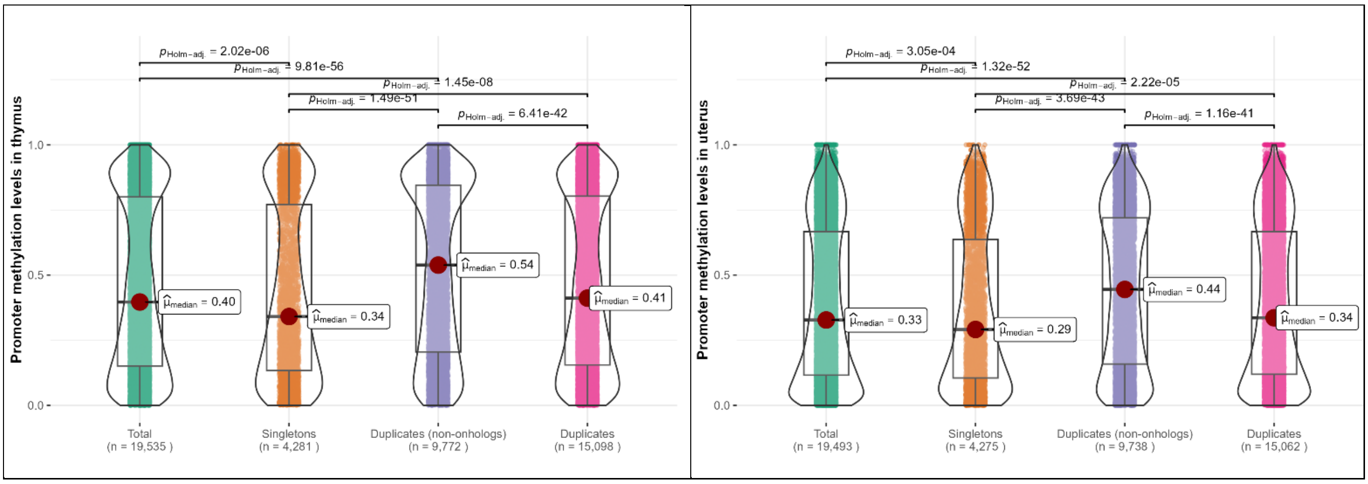

Promoter methylation levels in thymus
$p_{Holm-adj.}$ = 2.02e-06
$p_{Holm-adj.}$ = 9.81e-56
$p_{Holm-adj.}$ = 1.45e-08
$p_{Holm-adj.}$ = 1.49e-51
$p_{Holm-adj.}$ = 6.41e-42
$\widehat{\mu}_{median}$ = 0.40
$\widehat{\mu}_{median}$ = 0.34
$\widehat{\mu}_{median}$ = 0.54
$\widehat{\mu}_{median}$ = 0.41
Total (n = 19,535)
Singletons (n = 4,281)
Duplicates (non-onhologs) (n = 9,772)
Duplicates (n = 15,098)
Promoter methylation levels in uterus
$p_{Holm-adj.}$ = 3.05e-04
$p_{Holm-adj.}$ = 1.32e-52
$p_{Holm-adj.}$ = 2.22e-05
$p_{Holm-adj.}$ = 3.69e-43
$p_{Holm-adj.}$ = 1.16e-41
$\widehat{\mu}_{median}$ = 0.33
$\widehat{\mu}_{median}$ = 0.29
$\widehat{\mu}_{median}$ = 0.44
$\widehat{\mu}_{median}$ = 0.34
Total (n = 19,493)
Singletons (n = 4,275)
Duplicates (non-onhologs) (n = 9,738)
Duplicates (n = 15,062)

2. Comparison of promoter methylation levels among the different kinds of orthologs

2.1. Human genes

Below are the representations of levels of promoter methylation in the 10 human tissues analyzed among the different kinds of human-mouse orthologs. In each plot, each dot represents a gene. The horizontal lines indicate significant differences based on two-sided Dunn's pairwise tests with Holm correction for multiple comparisons. Comparisons were deemed significant if corrected p-values were lower than 0.05.

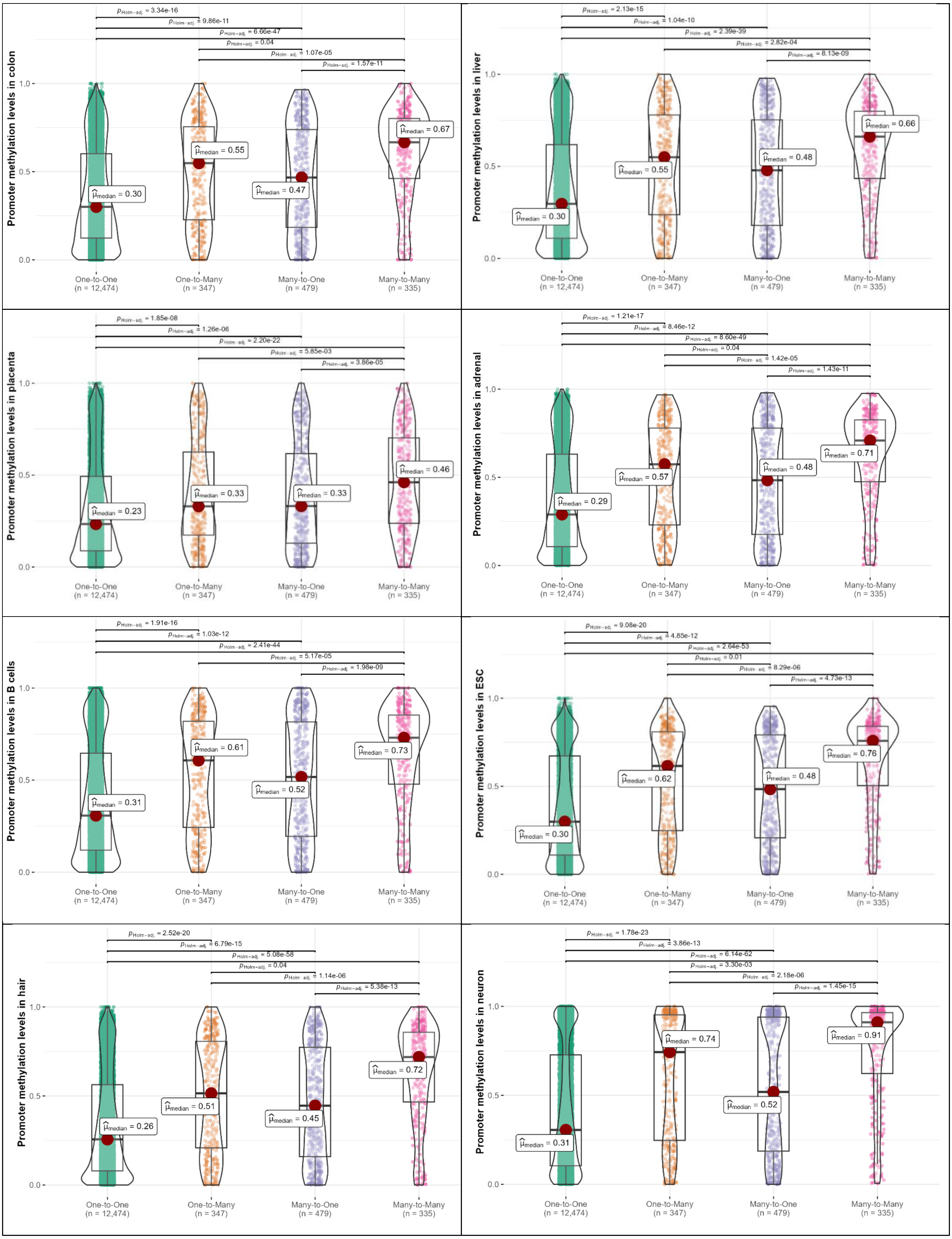

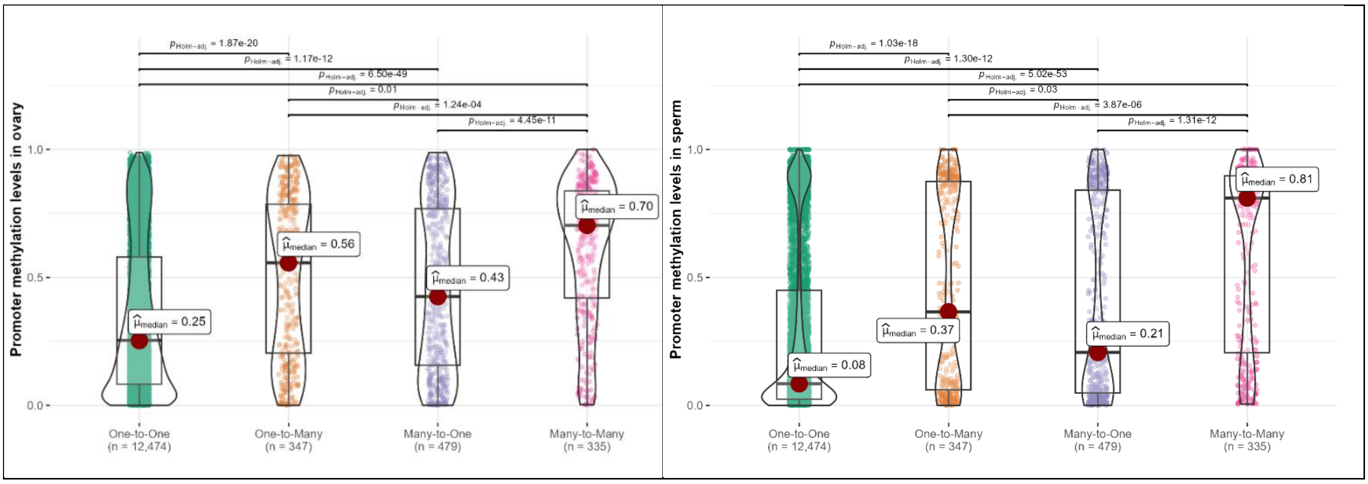

2.2. Mouse genes

Below are the representations of levels of promoter methylation in the 16 mouse tissues analyzed among the different kinds of mouse-human orthologs. In each plot, each dot represents a gene. The horizontal lines indicate significant differences based on two-sided Dunn's pairwise tests with Holm correction for multiple comparisons. Comparisons were deemed significant if corrected p-values were lower than 0.05.

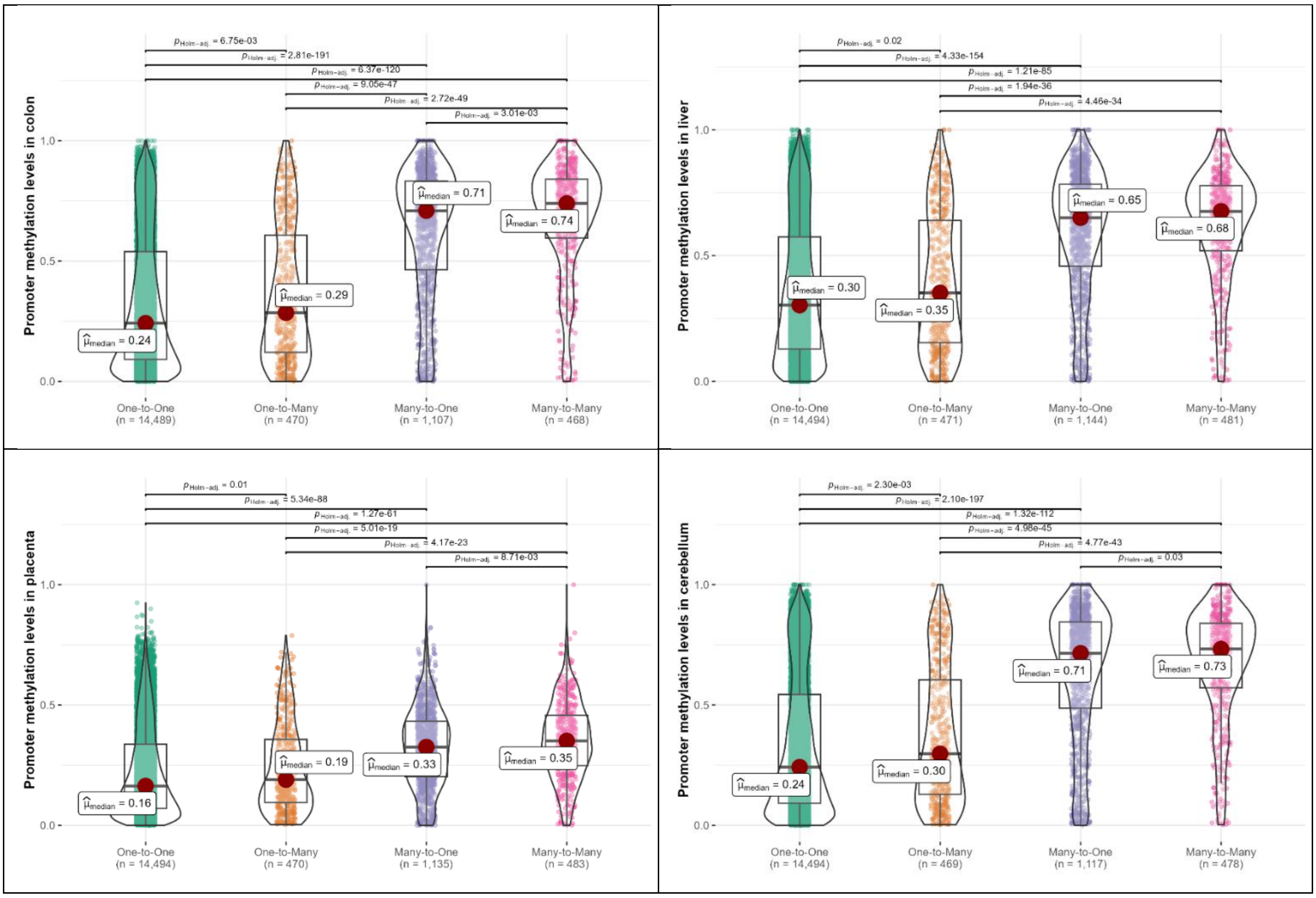

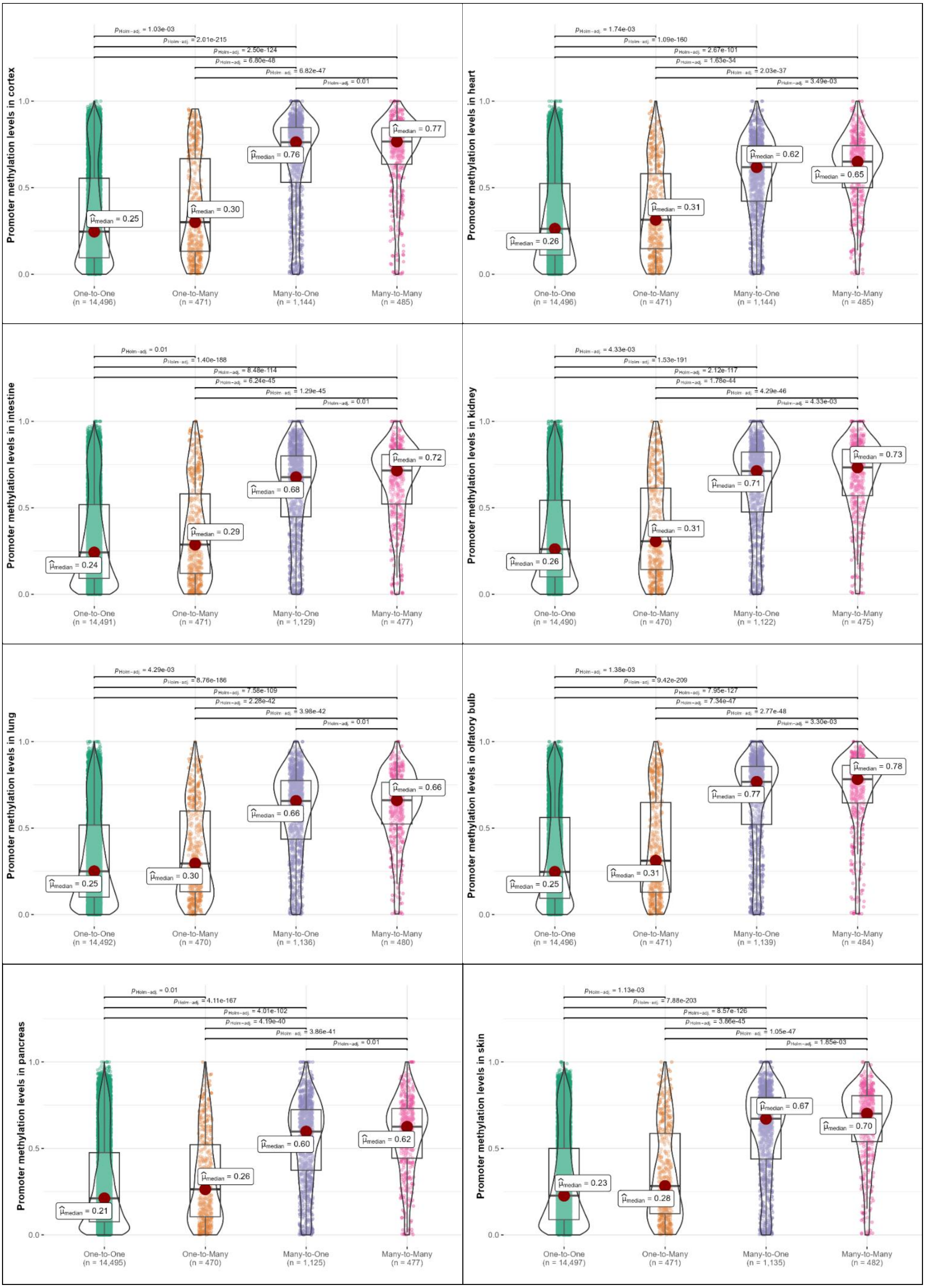
Promoter methylation levels in cortex
$p_{Holm-adj.}$ = 1.03e-03
$p_{Holm-adj.}$ = 2.01e-215
$p_{Holm-adj.}$ = 2.50e-124
$p_{Holm-adj.}$ = 6.80e-48
$p_{Holm-adj.}$ = 6.82e-47
$p_{Holm-adj.}$ = 0.01
$\widehat{\mu}_{median}$ = 0.25
$\widehat{\mu}_{median}$ = 0.30
$\widehat{\mu}_{median}$ = 0.76
$\widehat{\mu}_{median}$ = 0.77
One-to-One (n = 14,496)
One-to-Many (n = 471)
Many-to-One (n = 1,144)
Many-to-Many (n = 485)
Promoter methylation levels in heart
$p_{Holm-adj.}$ = 1.74e-03
$p_{Holm-adj.}$ = 1.09e-160
$p_{Holm-adj.}$ = 2.67e-101
$p_{Holm-adj.}$ = 1.63e-34
$p_{Holm-adj.}$ = 2.03e-37
$p_{Holm-adj.}$ = 3.49e-03
$\widehat{\mu}_{median}$ = 0.26
$\widehat{\mu}_{median}$ = 0.31
$\widehat{\mu}_{median}$ = 0.62
$\widehat{\mu}_{median}$ = 0.65
One-to-One (n = 14,496)
One-to-Many (n = 471)
Many-to-One (n = 1,144)
Many-to-Many (n = 485)
Promoter methylation levels in intestine
$p_{Holm-adj.}$ = 0.01
$p_{Holm-adj.}$ = 1.40e-188
$p_{Holm-adj.}$ = 8.48e-114
$p_{Holm-adj.}$ = 6.24e-45
$p_{Holm-adj.}$ = 1.29e-45
$p_{Holm-adj.}$ = 0.01
$\widehat{\mu}_{median}$ = 0.24
$\widehat{\mu}_{median}$ = 0.29
$\widehat{\mu}_{median}$ = 0.68
$\widehat{\mu}_{median}$ = 0.72
One-to-One (n = 14,491)
One-to-Many (n = 471)
Many-to-One (n = 1,129)
Many-to-Many (n = 477)
Promoter methylation levels in kidney
$p_{Holm-adj.}$ = 4.33e-03
$p_{Holm-adj.}$ = 1.53e-191
$p_{Holm-adj.}$ = 2.12e-117
$p_{Holm-adj.}$ = 1.78e-44
$p_{Holm-adj.}$ = 4.29e-46
$p_{Holm-adj.}$ = 4.33e-03
$\widehat{\mu}_{median}$ = 0.26
$\widehat{\mu}_{median}$ = 0.31
$\widehat{\mu}_{median}$ = 0.71
$\widehat{\mu}_{median}$ = 0.73
One-to-One (n = 14,490)
One-to-Many (n = 470)
Many-to-One (n = 1,122)
Many-to-Many (n = 475)
Promoter methylation levels in lung
$p_{Holm-adj.}$ = 4.29e-03
$p_{Holm-adj.}$ = 8.76e-186
$p_{Holm-adj.}$ = 7.58e-109
$p_{Holm-adj.}$ = 2.28e-42
$p_{Holm-adj.}$ = 3.98e-42
$p_{Holm-adj.}$ = 0.01
$\widehat{\mu}_{median}$ = 0.25
$\widehat{\mu}_{median}$ = 0.30
$\widehat{\mu}_{median}$ = 0.66
$\widehat{\mu}_{median}$ = 0.66
One-to-One (n = 14,492)
One-to-Many (n = 470)
Many-to-One (n = 1,136)
Many-to-Many (n = 480)
Promoter methylation levels in olfatory bulb
$p_{Holm-adj.}$ = 1.38e-03
$p_{Holm-adj.}$ = 9.42e-209
$p_{Holm-adj.}$ = 7.95e-127
$p_{Holm-adj.}$ = 7.34e-47
$p_{Holm-adj.}$ = 2.77e-48
$p_{Holm-adj.}$ = 3.30e-03
$\widehat{\mu}_{median}$ = 0.25
$\widehat{\mu}_{median}$ = 0.31
$\widehat{\mu}_{median}$ = 0.77
$\widehat{\mu}_{median}$ = 0.78
One-to-One (n = 14,496)
One-to-Many (n = 471)
Many-to-One (n = 1,139)
Many-to-Many (n = 484)
Promoter methylation levels in pancreas
$p_{Holm-adj.}$ = 0.01
$p_{Holm-adj.}$ = 4.11e-167
$p_{Holm-adj.}$ = 4.01e-102
$p_{Holm-adj.}$ = 4.19e-40
$p_{Holm-adj.}$ = 3.86e-41
$p_{Holm-adj.}$ = 0.01
$\widehat{\mu}_{median}$ = 0.21
$\widehat{\mu}_{median}$ = 0.26
$\widehat{\mu}_{median}$ = 0.60
$\widehat{\mu}_{median}$ = 0.62
One-to-One (n = 14,495)
One-to-Many (n = 470)
Many-to-One (n = 1,125)
Many-to-Many (n = 477)
Promoter methylation levels in skin
$p_{Holm-adj.}$ = 1.13e-03
$p_{Holm-adj.}$ = 7.88e-203
$p_{Holm-adj.}$ = 8.57e-126
$p_{Holm-adj.}$ = 3.86e-45
$p_{Holm-adj.}$ = 1.05e-47
$p_{Holm-adj.}$ = 1.85e-03
$\widehat{\mu}_{median}$ = 0.23
$\widehat{\mu}_{median}$ = 0.28
$\widehat{\mu}_{median}$ = 0.67
$\widehat{\mu}_{median}$ = 0.70
One-to-One (n = 14,497)
One-to-Many (n = 471)
Many-to-One (n = 1,135)
Many-to-Many (n = 482)

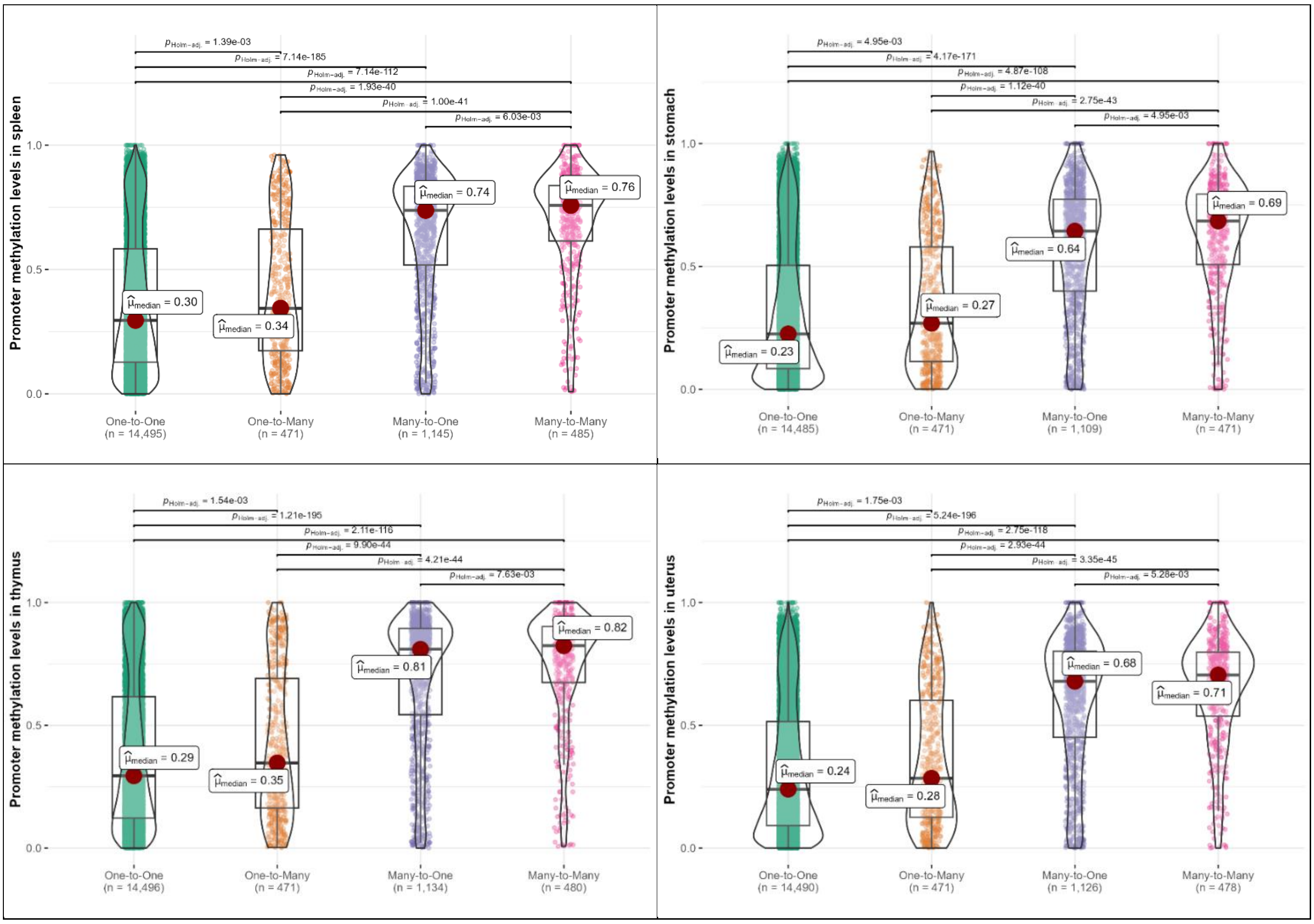
$p_{Holm-adj.}$ = 1.39e-03
$p_{Holm-adj.}$ = 7.14e-185
$p_{Holm-adj.}$ = 7.14e-112
$p_{Holm-adj.}$ = 1.93e-40
$p_{Holm-adj.}$ = 1.00e-41
$p_{Holm-adj.}$ = 6.03e-03
Promoter methylation levels in spleen
1.0
0.5
0.0
$\widehat{\mu}_{median}$ = 0.30
$\widehat{\mu}_{median}$ = 0.34
$\widehat{\mu}_{median}$ = 0.74
$\widehat{\mu}_{median}$ = 0.76
One-to-One (n = 14,495)
One-to-Many (n = 471)
Many-to-One (n = 1,145)
Many-to-Many (n = 485)
$p_{Holm-adj.}$ = 4.95e-03
$p_{Holm-adj.}$ = 4.17e-171
$p_{Holm-adj.}$ = 4.87e-108
$p_{Holm-adj.}$ = 1.12e-40
$p_{Holm-adj.}$ = 2.75e-43
$p_{Holm-adj.}$ = 4.95e-03
Promoter methylation levels in stomach
1.0
0.5
0.0
$\widehat{\mu}_{median}$ = 0.23
$\widehat{\mu}_{median}$ = 0.27
$\widehat{\mu}_{median}$ = 0.64
$\widehat{\mu}_{median}$ = 0.69
One-to-One (n = 14,485)
One-to-Many (n = 471)
Many-to-One (n = 1,109)
Many-to-Many (n = 471)
$p_{Holm-adj.}$ = 1.54e-03
$p_{Holm-adj.}$ = 1.21e-195
$p_{Holm-adj.}$ = 2.11e-116
$p_{Holm-adj.}$ = 9.90e-44
$p_{Holm-adj.}$ = 4.21e-44
$p_{Holm-adj.}$ = 7.63e-03
Promoter methylation levels in thymus
1.0
0.5
0.0
$\widehat{\mu}_{median}$ = 0.29
$\widehat{\mu}_{median}$ = 0.35
$\widehat{\mu}_{median}$ = 0.81
$\widehat{\mu}_{median}$ = 0.82
One-to-One (n = 14,496)
One-to-Many (n = 471)
Many-to-One (n = 1,134)
Many-to-Many (n = 480)
$p_{Holm-adj.}$ = 1.75e-03
$p_{Holm-adj.}$ = 5.24e-196
$p_{Holm-adj.}$ = 2.75e-118
$p_{Holm-adj.}$ = 2.93e-44
$p_{Holm-adj.}$ = 3.35e-45
$p_{Holm-adj.}$ = 5.28e-03
Promoter methylation levels in uterus
1.0
0.5
0.0
$\widehat{\mu}_{median}$ = 0.24
$\widehat{\mu}_{median}$ = 0.28
$\widehat{\mu}_{median}$ = 0.68
$\widehat{\mu}_{median}$ = 0.71
One-to-One (n = 14,490)
One-to-Many (n = 471)
Many-to-One (n = 1,126)
Many-to-Many (n = 478)

3. Correlation of promoter methylation levels of recent duplicates with the Gene Order Conservation (GOC) scores

3.1. Human genes

Below are the representations of the promoter methylation levels among many-to-one genes (duplicated in human, but not duplicated in mouse) with different Gene Order Conservation (GOC) scores, in the 10 human tissues analyzed. The horizontal lines indicate significant differences based on two-sided Dunn's pairwise tests with Holm correction for multiple comparisons. Comparisons were deemed significant if corrected p-values were lower than 0.05.

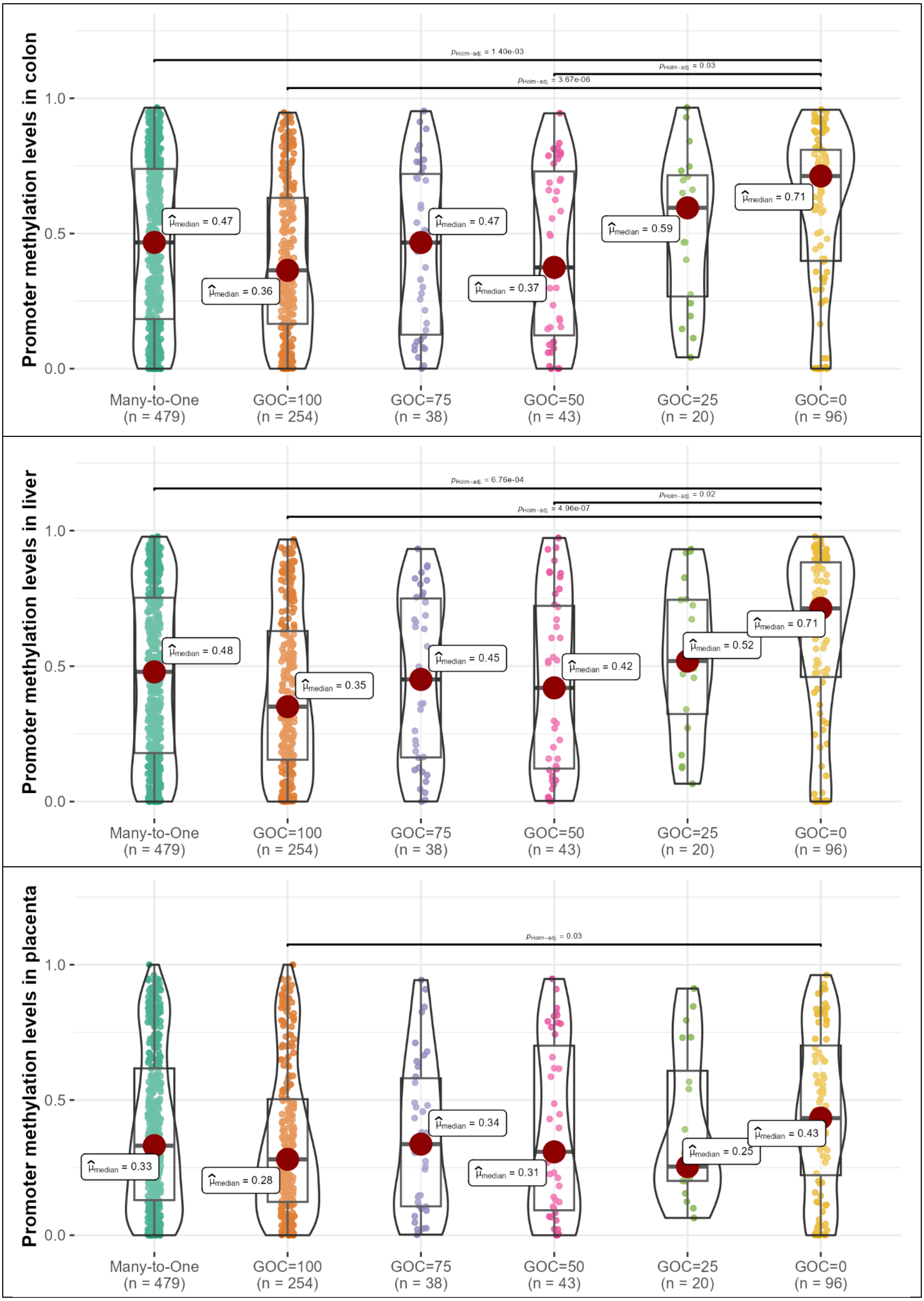

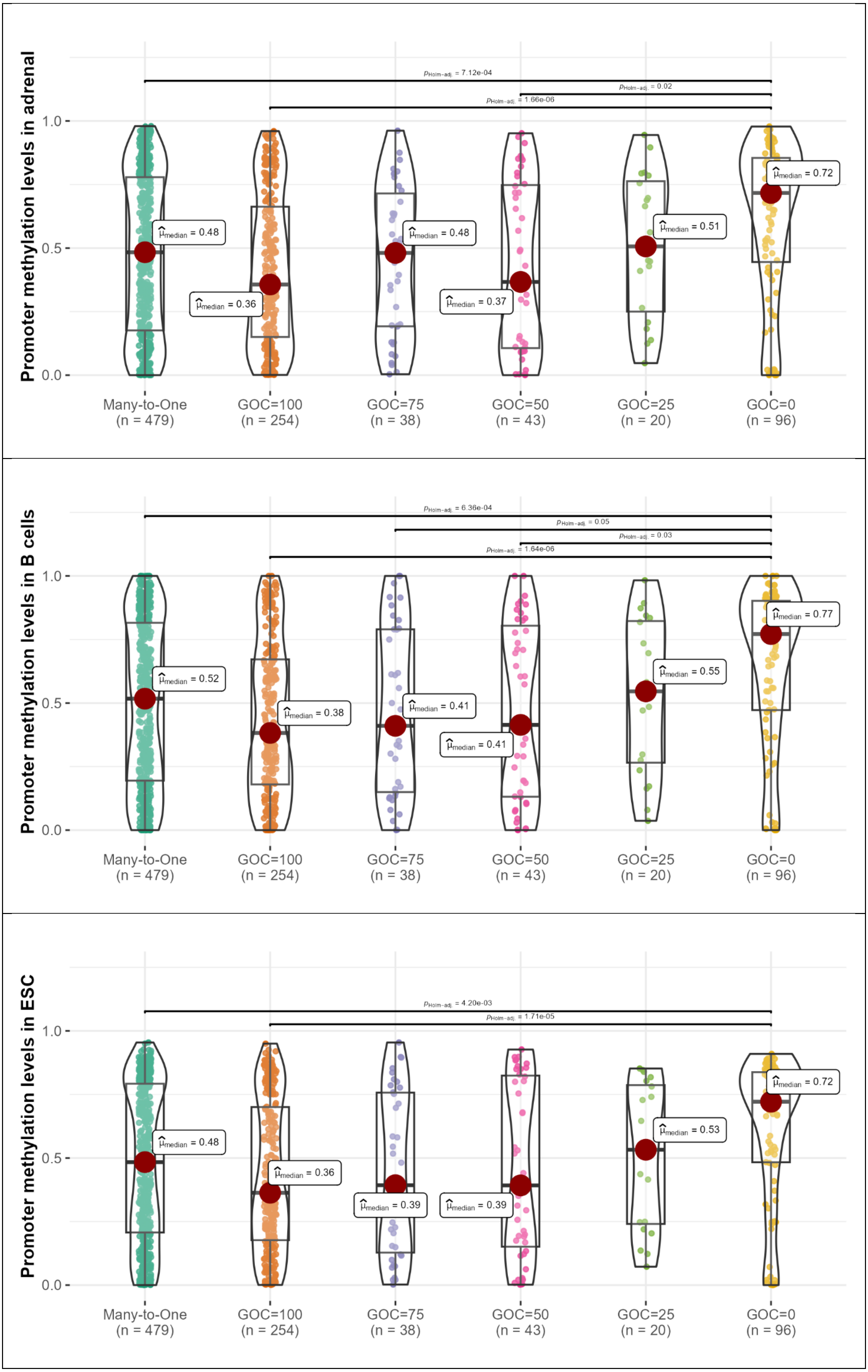

Promoter methylation levels in adrenal
pHolm-adj. = 7.12e-04
pHolm-adj. = 0.02
pHolm-adj. = 1.66e-06
μ̂median = 0.48
μ̂median = 0.36
μ̂median = 0.48
μ̂median = 0.37
μ̂median = 0.51
μ̂median = 0.72
Promoter methylation levels in B cells
pHolm-adj. = 6.36e-04
pHolm-adj. = 0.05
pHolm-adj. = 0.03
pHolm-adj. = 1.64e-06
μ̂median = 0.52
μ̂median = 0.38
μ̂median = 0.41
μ̂median = 0.41
μ̂median = 0.55
μ̂median = 0.77
Promoter methylation levels in ESC
pHolm-adj. = 4.20e-03
pHolm-adj. = 1.71e-05
μ̂median = 0.48
μ̂median = 0.36
μ̂median = 0.39
μ̂median = 0.39
μ̂median = 0.53
μ̂median = 0.72
0.0
0.5
1.0
Many-to-One (n = 479)
GOC=100 (n = 254)
GOC=75 (n = 38)
GOC=50 (n = 43)
GOC=25 (n = 20)
GOC=0 (n = 96)

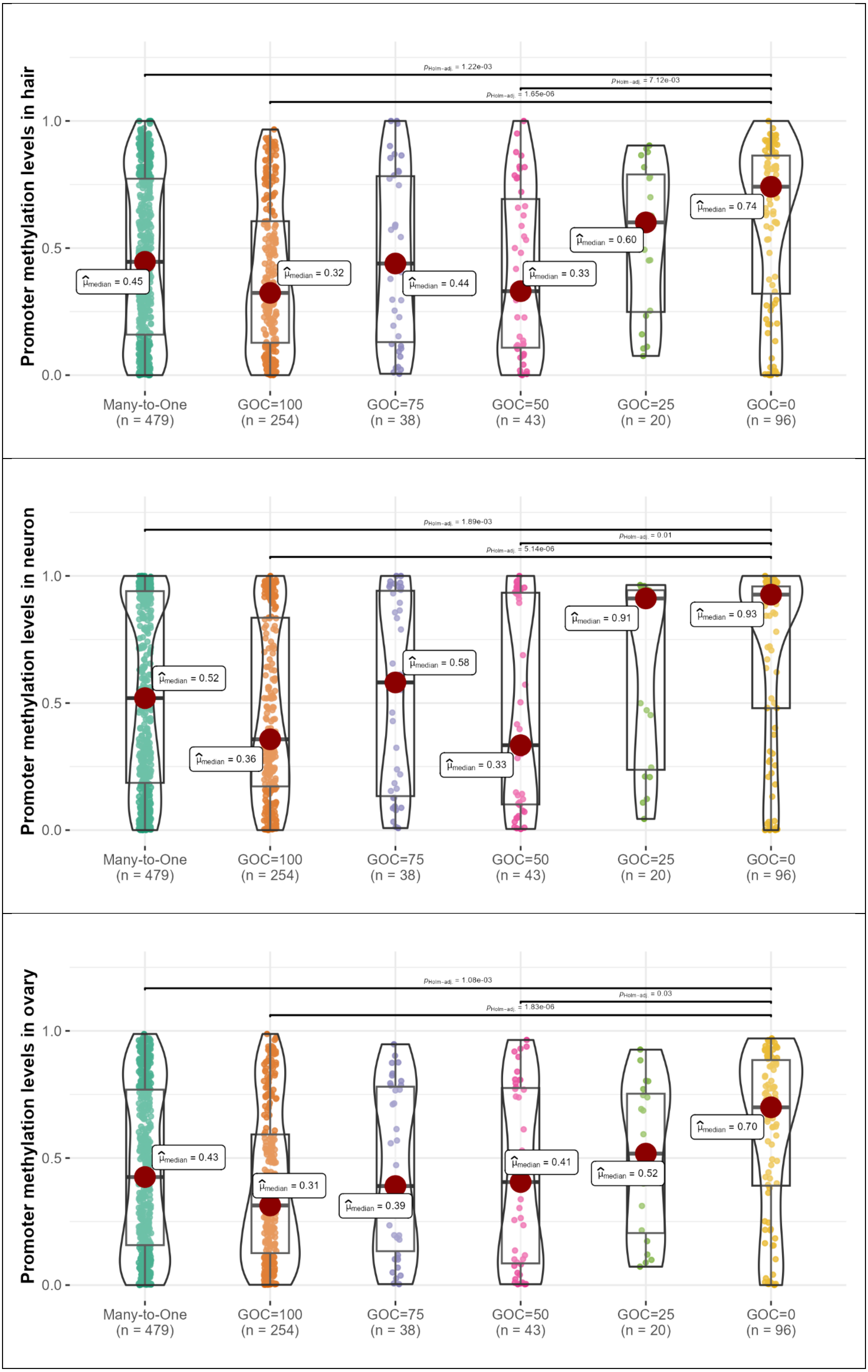

Promoter methylation levels in hair
Promoter methylation levels in neuron
Promoter methylation levels in ovary
Many-to-One (n = 479)
GOC=100 (n = 254)
GOC=75 (n = 38)
GOC=50 (n = 43)
GOC=25 (n = 20)
GOC=0 (n = 96)

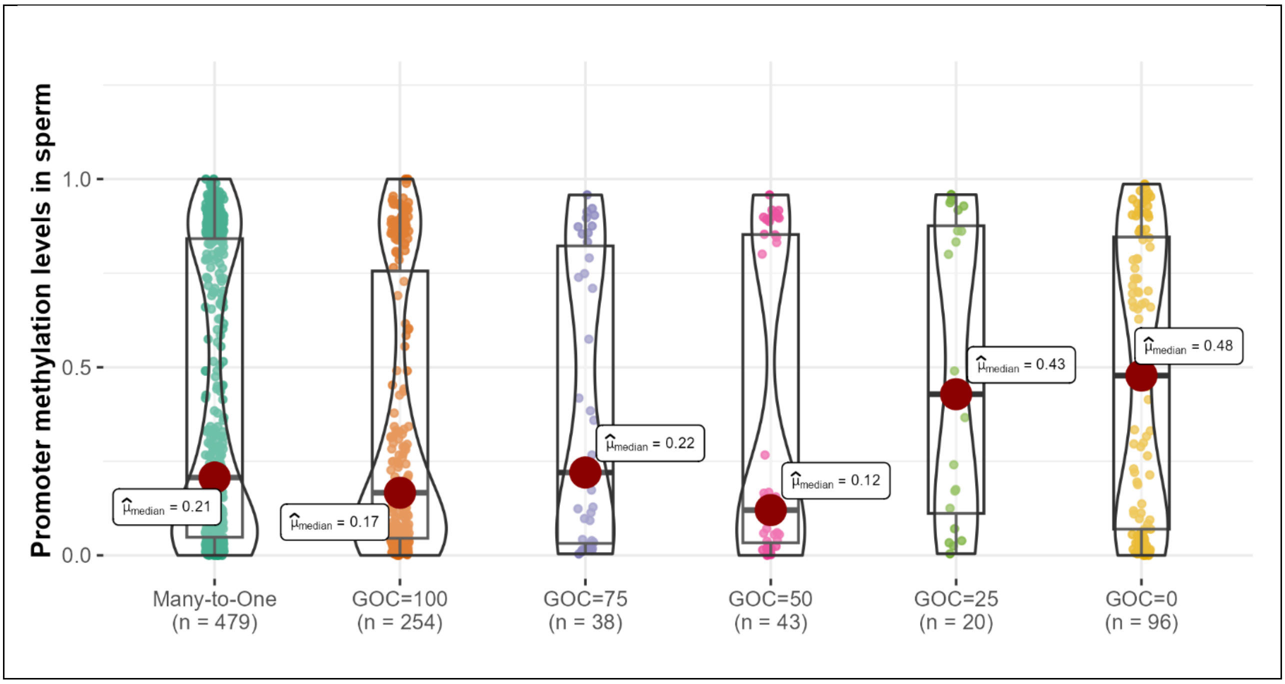

3.2. Mouse genes

Below are the representations of the promoter methylation levels among many-to-one genes (duplicated in mouse, but not duplicated in human) with different Gene Order Conservation (GOC) scores, in the 16 mouse tissues analyzed. The horizontal lines indicate significant differences based on two-sided Dunn's pairwise tests with Holm correction for multiple comparisons. Comparisons were deemed significant if corrected p-values were lower than 0.05.

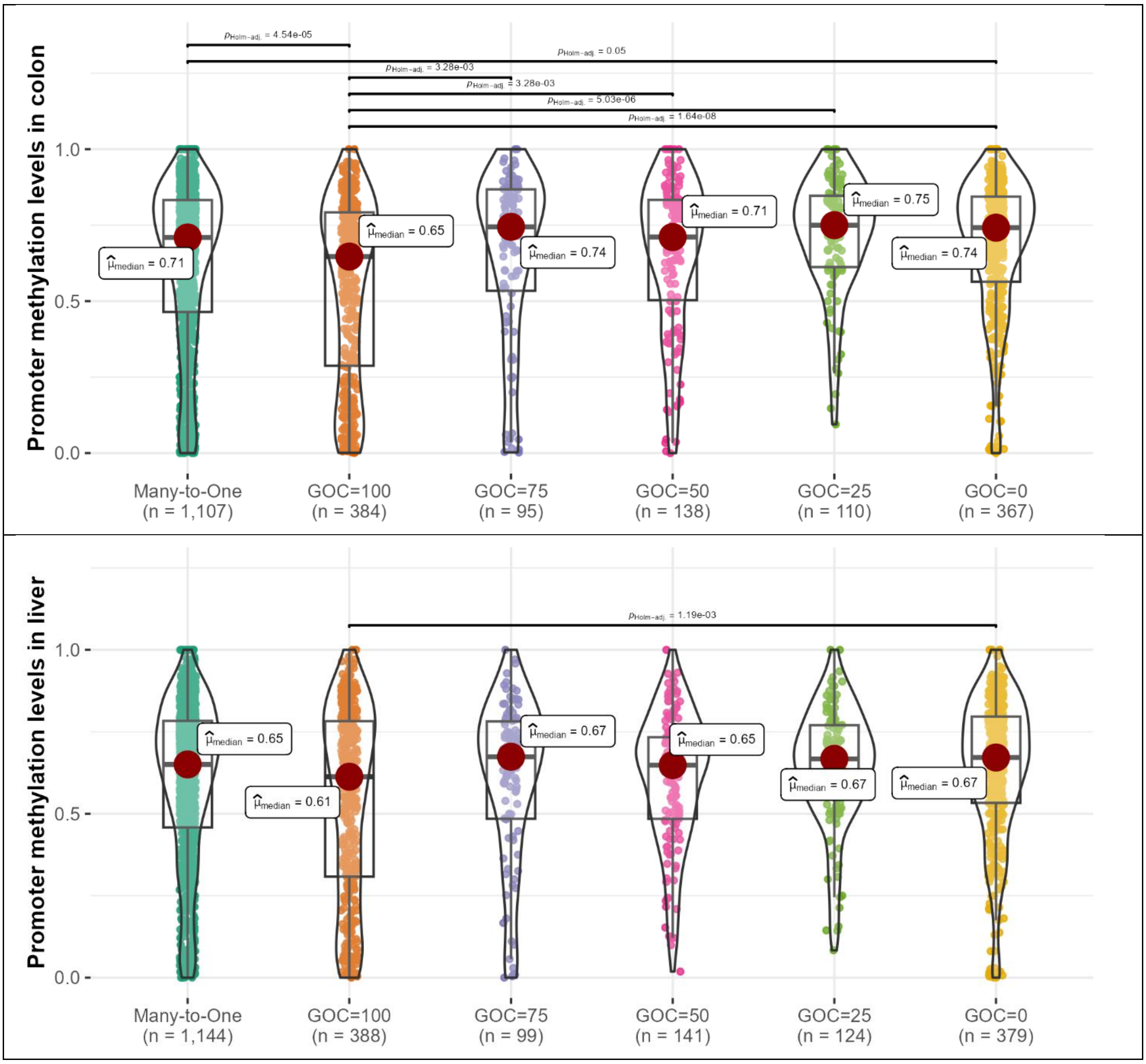

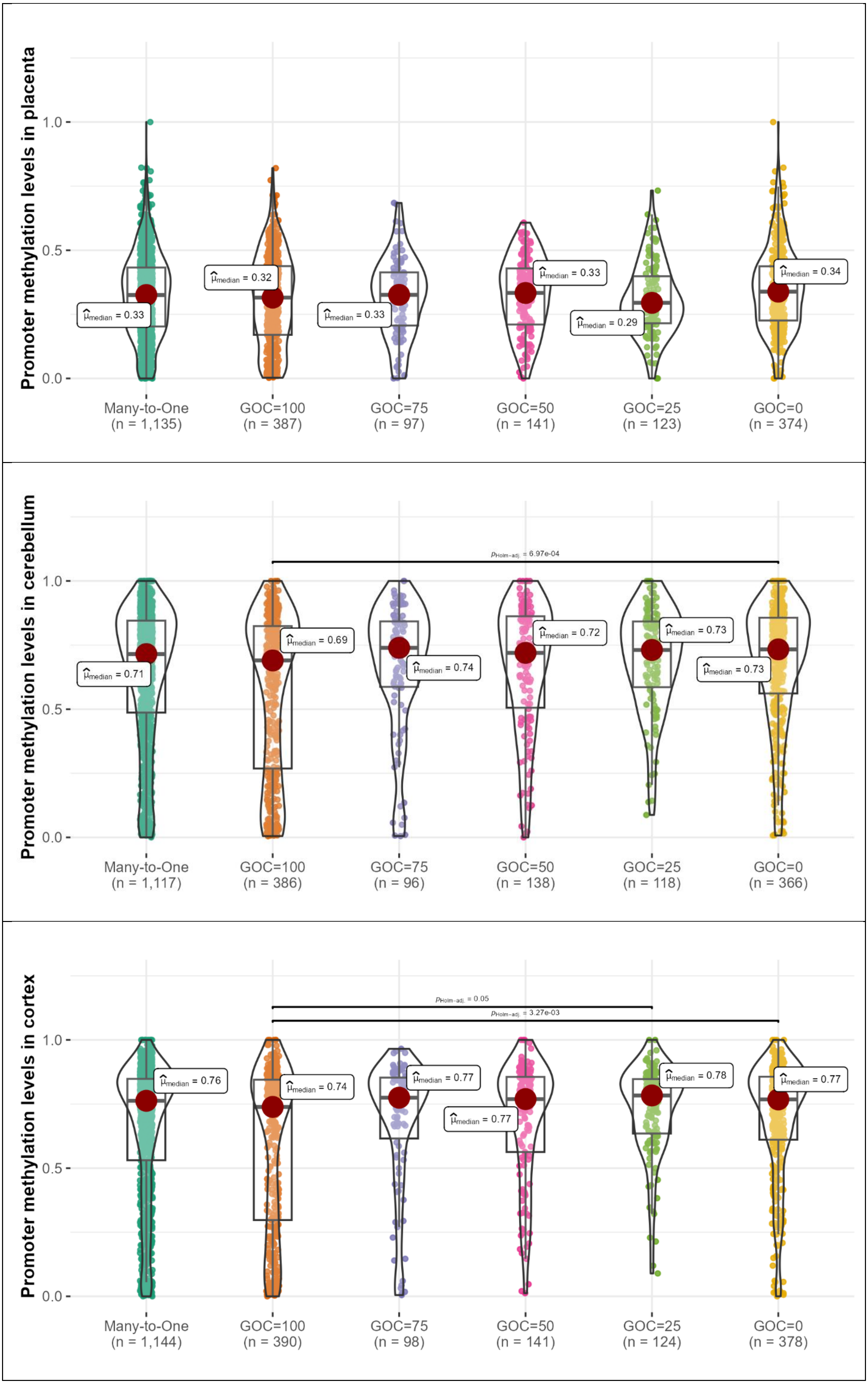

Promoter methylation levels in placenta
1.0
0.5
0.0
$\hat{\mu}_{median}$ = 0.33
$\hat{\mu}_{median}$ = 0.32
$\hat{\mu}_{median}$ = 0.33
$\hat{\mu}_{median}$ = 0.33
$\hat{\mu}_{median}$ = 0.29
$\hat{\mu}_{median}$ = 0.34
Many-to-One (n = 1,135)
GOC=100 (n = 387)
GOC=75 (n = 97)
GOC=50 (n = 141)
GOC=25 (n = 123)
GOC=0 (n = 374)
Promoter methylation levels in cerebellum
$p_{Holm-adj.}$ = 6.97e-04
$\hat{\mu}_{median}$ = 0.71
$\hat{\mu}_{median}$ = 0.69
$\hat{\mu}_{median}$ = 0.74
$\hat{\mu}_{median}$ = 0.72
$\hat{\mu}_{median}$ = 0.73
$\hat{\mu}_{median}$ = 0.73
Many-to-One (n = 1,117)
GOC=100 (n = 386)
GOC=75 (n = 96)
GOC=50 (n = 138)
GOC=25 (n = 118)
GOC=0 (n = 366)
Promoter methylation levels in cortex
$p_{Holm-adj.}$ = 0.05
$p_{Holm-adj.}$ = 3.27e-03
$\hat{\mu}_{median}$ = 0.76
$\hat{\mu}_{median}$ = 0.74
$\hat{\mu}_{median}$ = 0.77
$\hat{\mu}_{median}$ = 0.77
$\hat{\mu}_{median}$ = 0.78
$\hat{\mu}_{median}$ = 0.77
Many-to-One (n = 1,144)
GOC=100 (n = 390)
GOC=75 (n = 98)
GOC=50 (n = 141)
GOC=25 (n = 124)
GOC=0 (n = 378)

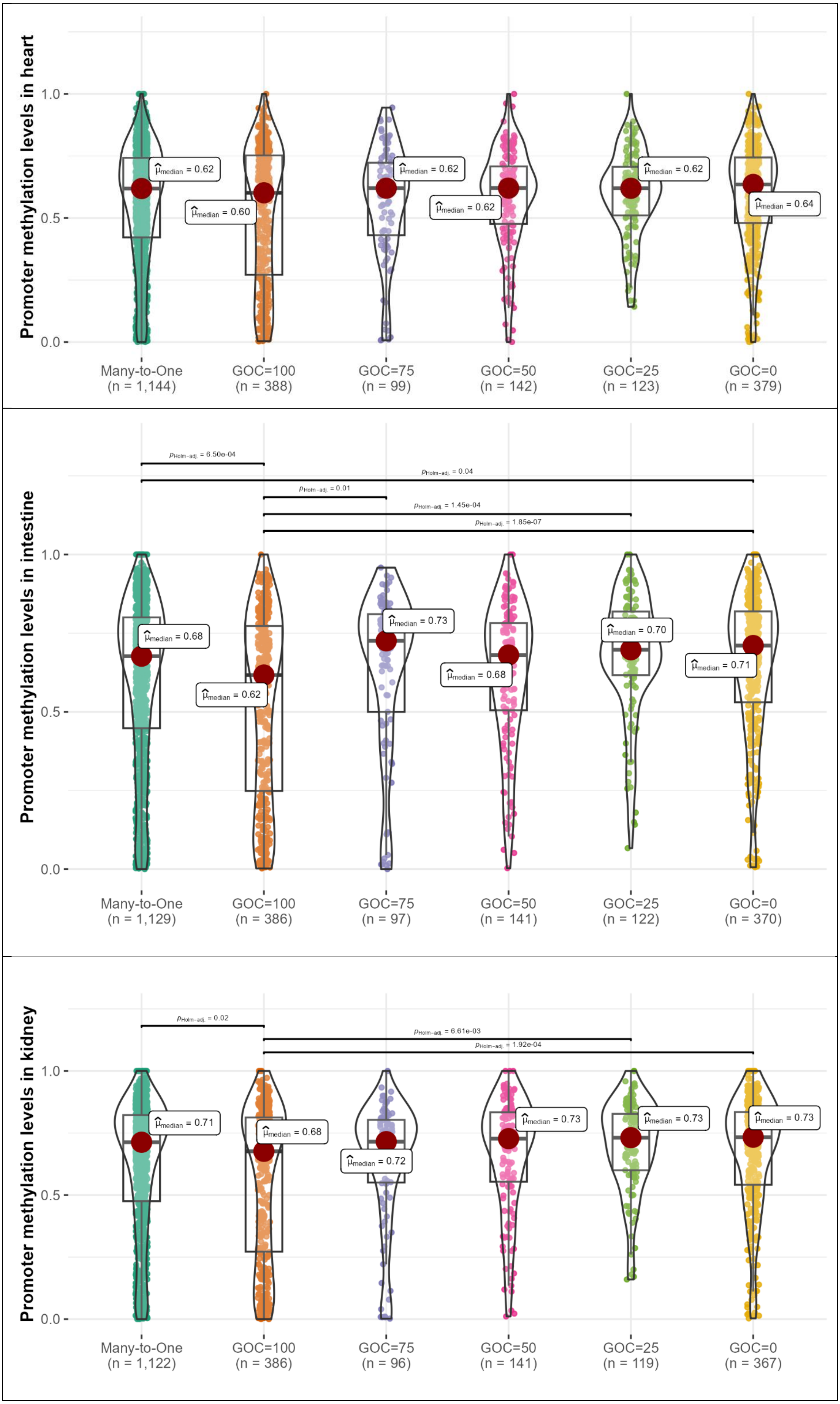

Promoter methylation levels in heart
1.0
0.5
0.0
μ̂median = 0.62
μ̂median = 0.60
μ̂median = 0.62
μ̂median = 0.62
μ̂median = 0.62
μ̂median = 0.64
Many-to-One (n = 1,144)
GOC=100 (n = 388)
GOC=75 (n = 99)
GOC=50 (n = 142)
GOC=25 (n = 123)
GOC=0 (n = 379)
Promoter methylation levels in intestine
pHolm-adj. = 6.50e-04
pHolm-adj. = 0.04
pHolm-adj. = 0.01
pHolm-adj. = 1.45e-04
pHolm-adj. = 1.85e-07
μ̂median = 0.68
μ̂median = 0.62
μ̂median = 0.73
μ̂median = 0.68
μ̂median = 0.70
μ̂median = 0.71
Many-to-One (n = 1,129)
GOC=100 (n = 386)
GOC=75 (n = 97)
GOC=50 (n = 141)
GOC=25 (n = 122)
GOC=0 (n = 370)
Promoter methylation levels in kidney
pHolm-adj. = 0.02
pHolm-adj. = 6.61e-03
pHolm-adj. = 1.92e-04
μ̂median = 0.71
μ̂median = 0.68
μ̂median = 0.72
μ̂median = 0.73
μ̂median = 0.73
μ̂median = 0.73
Many-to-One (n = 1,122)
GOC=100 (n = 386)
GOC=75 (n = 96)
GOC=50 (n = 141)
GOC=25 (n = 119)
GOC=0 (n = 367)

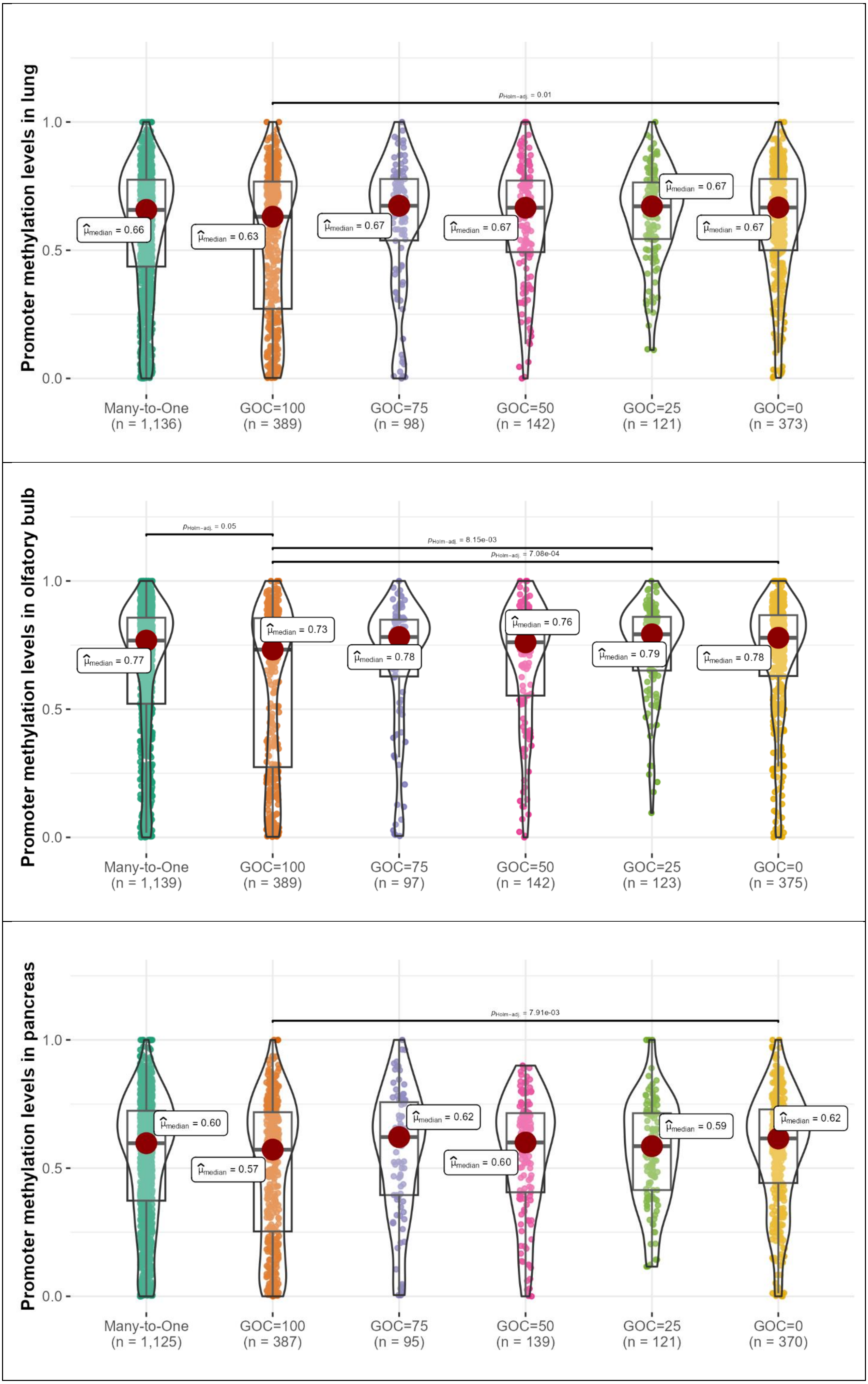
Promoter methylation levels in lung
pHolm-adj. = 0.01
μ̂median = 0.66
μ̂median = 0.63
μ̂median = 0.67
μ̂median = 0.67
μ̂median = 0.67
μ̂median = 0.67
1.0
0.5
0.0
Many-to-One (n = 1,136)
GOC=100 (n = 389)
GOC=75 (n = 98)
GOC=50 (n = 142)
GOC=25 (n = 121)
GOC=0 (n = 373)
Promoter methylation levels in olfatory bulb
pHolm-adj. = 0.05
pHolm-adj. = 8.15e-03
pHolm-adj. = 7.08e-04
μ̂median = 0.77
μ̂median = 0.73
μ̂median = 0.78
μ̂median = 0.76
μ̂median = 0.79
μ̂median = 0.78
1.0
0.5
0.0
Many-to-One (n = 1,139)
GOC=100 (n = 389)
GOC=75 (n = 97)
GOC=50 (n = 142)
GOC=25 (n = 123)
GOC=0 (n = 375)
Promoter methylation levels in pancreas
pHolm-adj. = 7.91e-03
μ̂median = 0.60
μ̂median = 0.57
μ̂median = 0.62
μ̂median = 0.60
μ̂median = 0.59
μ̂median = 0.62
1.0
0.5
0.0
Many-to-One (n = 1,125)
GOC=100 (n = 387)
GOC=75 (n = 95)
GOC=50 (n = 139)
GOC=25 (n = 121)
GOC=0 (n = 370)

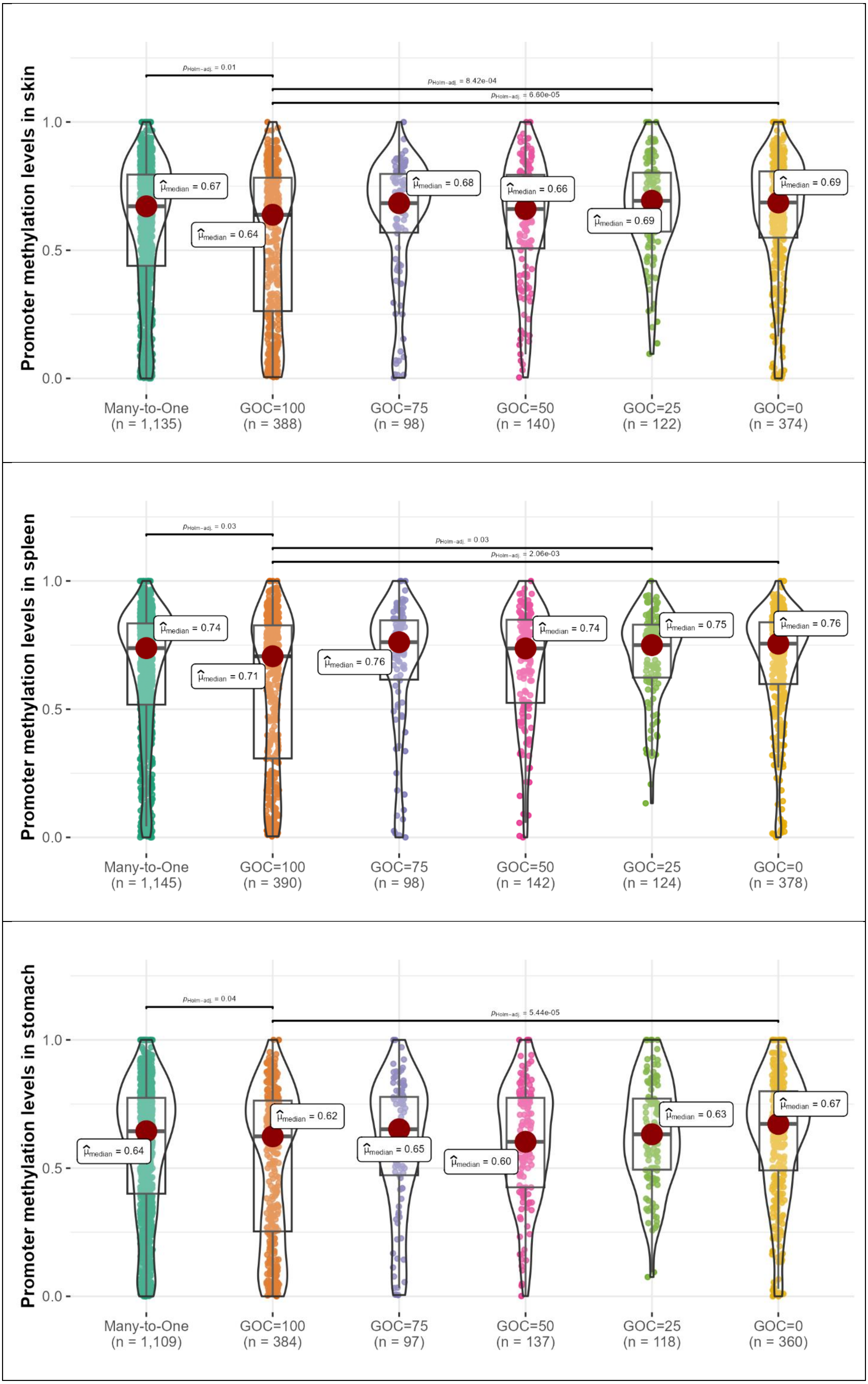

Promoter methylation levels in skin
p_Holm-adj. = 0.01
p_Holm-adj. = 8.42e-04
p_Holm-adj. = 6.60e-05
μ̂_median = 0.67
μ̂_median = 0.64
μ̂_median = 0.68
μ̂_median = 0.66
μ̂_median = 0.69
μ̂_median = 0.69
1.0
0.5
0.0
Many-to-One (n = 1,135)
GOC=100 (n = 388)
GOC=75 (n = 98)
GOC=50 (n = 140)
GOC=25 (n = 122)
GOC=0 (n = 374)
Promoter methylation levels in spleen
p_Holm-adj. = 0.03
p_Holm-adj. = 0.03
p_Holm-adj. = 2.06e-03
μ̂_median = 0.74
μ̂_median = 0.71
μ̂_median = 0.76
μ̂_median = 0.74
μ̂_median = 0.75
μ̂_median = 0.76
1.0
0.5
0.0
Many-to-One (n = 1,145)
GOC=100 (n = 390)
GOC=75 (n = 98)
GOC=50 (n = 142)
GOC=25 (n = 124)
GOC=0 (n = 378)
Promoter methylation levels in stomach
p_Holm-adj. = 0.04
p_Holm-adj. = 5.44e-05
μ̂_median = 0.64
μ̂_median = 0.62
μ̂_median = 0.65
μ̂_median = 0.60
μ̂_median = 0.63
μ̂_median = 0.67
1.0
0.5
0.0
Many-to-One (n = 1,109)
GOC=100 (n = 384)
GOC=75 (n = 97)
GOC=50 (n = 137)
GOC=25 (n = 118)
GOC=0 (n = 360)

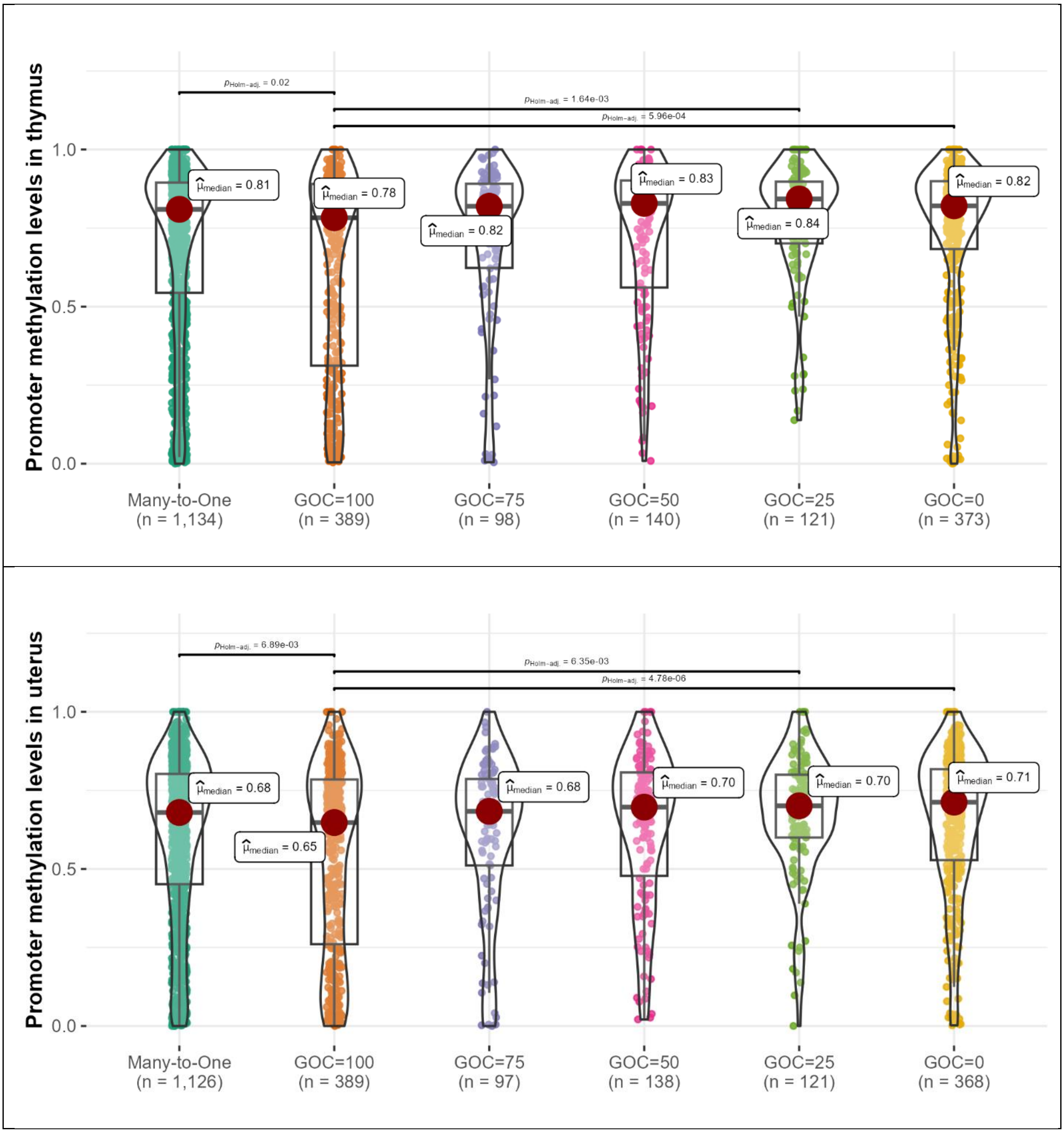

Promoter methylation levels in thymus
$p_{Holm-adj.}$ = 0.02
$p_{Holm-adj.}$ = 1.64e-03
$p_{Holm-adj.}$ = 5.96e-04
$\hat{\mu}_{median}$ = 0.81
$\hat{\mu}_{median}$ = 0.78
$\hat{\mu}_{median}$ = 0.82
$\hat{\mu}_{median}$ = 0.83
$\hat{\mu}_{median}$ = 0.84
$\hat{\mu}_{median}$ = 0.82
1.0
0.5
0.0
Many-to-One (n = 1,134)
GOC=100 (n = 389)
GOC=75 (n = 98)
GOC=50 (n = 140)
GOC=25 (n = 121)
GOC=0 (n = 373)
Promoter methylation levels in uterus
$p_{Holm-adj.}$ = 6.89e-03
$p_{Holm-adj.}$ = 6.35e-03
$p_{Holm-adj.}$ = 4.78e-06
$\hat{\mu}_{median}$ = 0.68
$\hat{\mu}_{median}$ = 0.65
$\hat{\mu}_{median}$ = 0.68
$\hat{\mu}_{median}$ = 0.70
$\hat{\mu}_{median}$ = 0.70
$\hat{\mu}_{median}$ = 0.71
1.0
0.5
0.0
Many-to-One (n = 1,126)
GOC=100 (n = 389)
GOC=75 (n = 97)
GOC=50 (n = 138)
GOC=25 (n = 121)
GOC=0 (n = 368)

4. Promoter methylation in trios duplicated in human but not in mouse

4.1. Comparison of promoter methylation of human daughter copies, human parental copies, and mouse orthologs

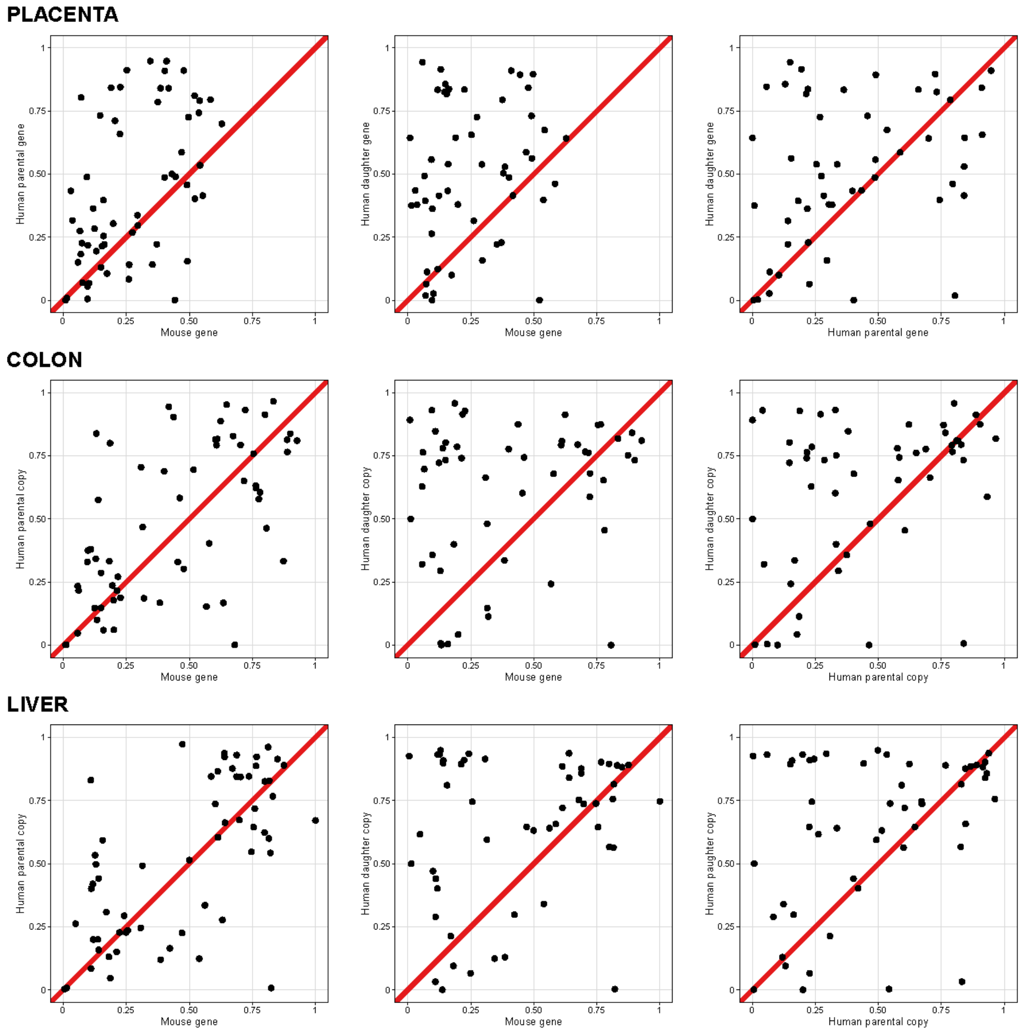

4.2. Comparison of promoter methylation of human daughter copies, human parental copies, and mouse orthologs: violin plots

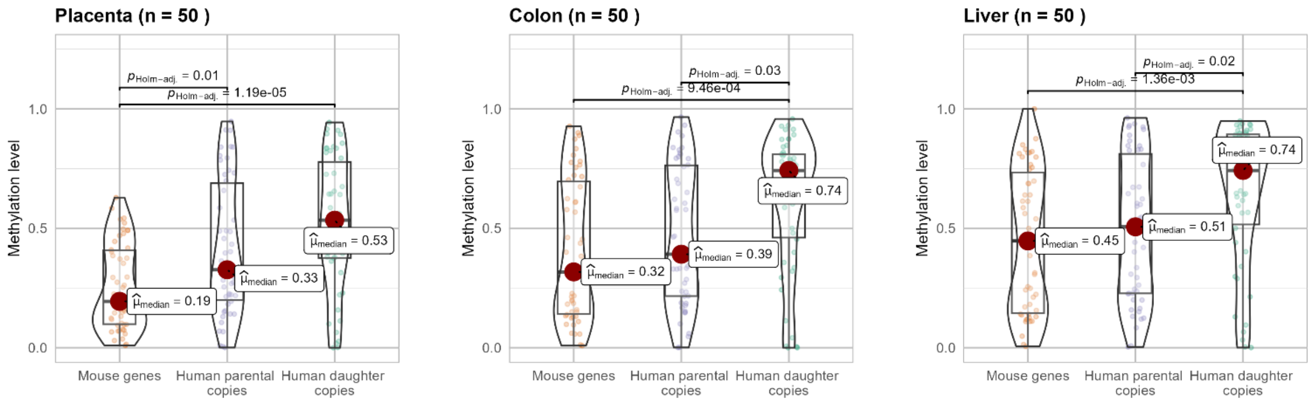

4.3. Comparison of promoter methylation of human daughter copies, human parental copies, and mouse orthologs without retrogenes

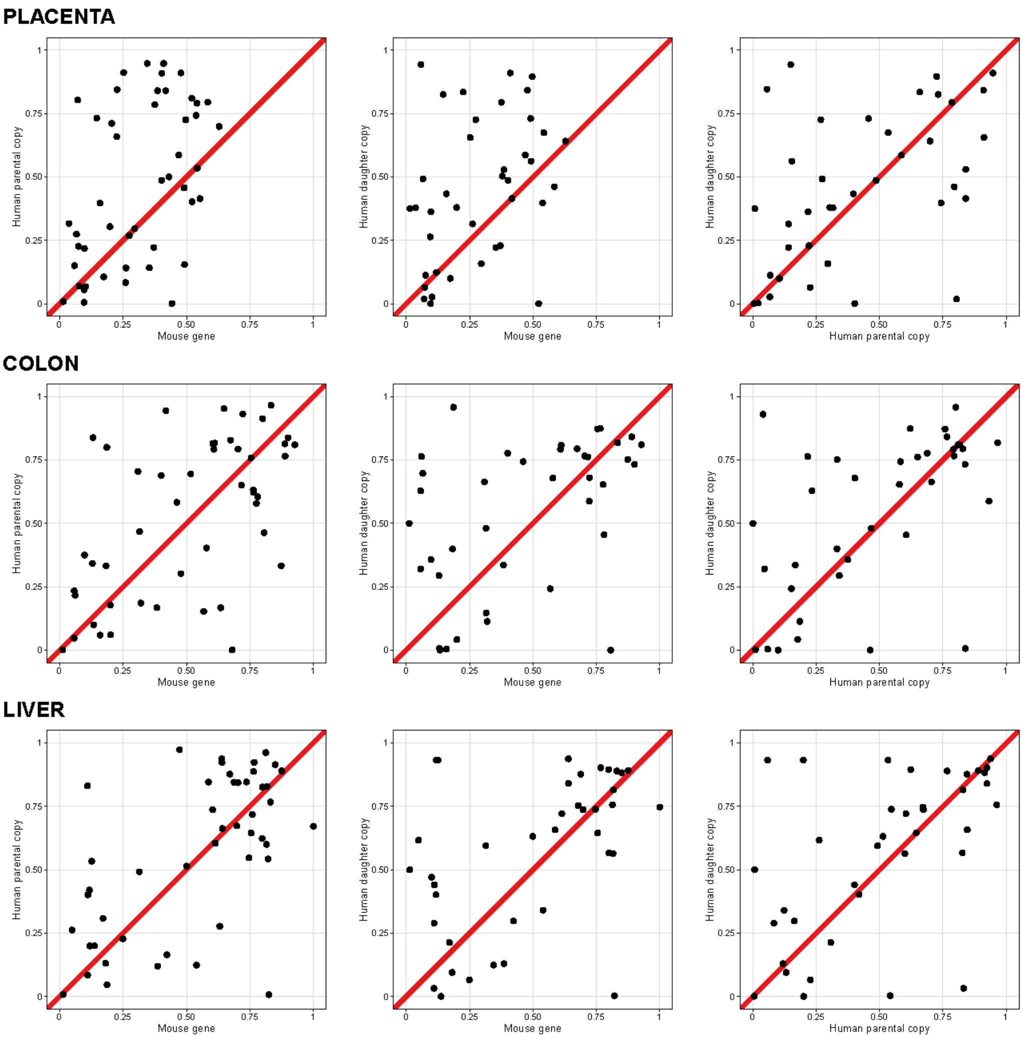

Each dot represents a pair of genes within a trio. For each graph, the red line corresponds to an equal amount of methylation in both genes.

4.4. Comparison of promoter methylation of human daughter copies, human parental copies, and mouse orthologs without retrogenes: violin plots

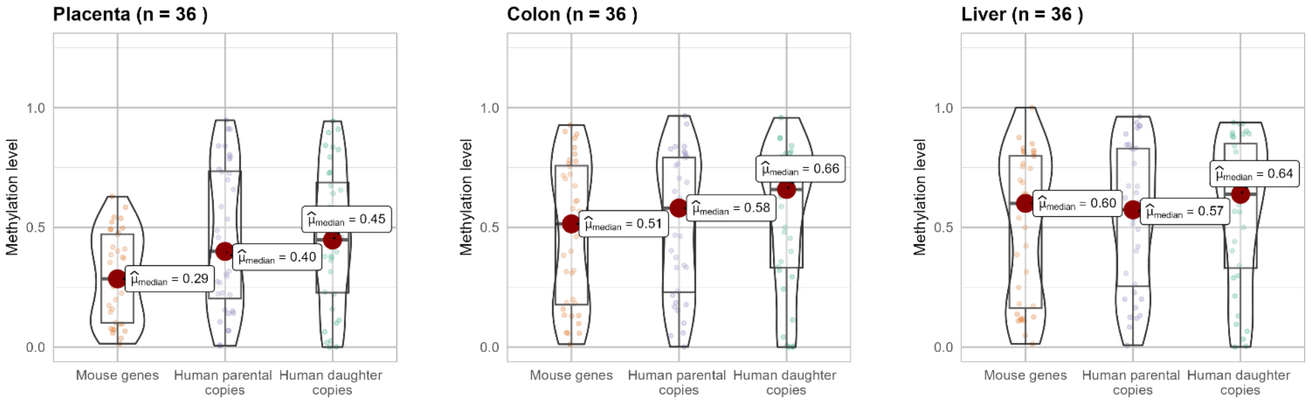

4.5. Comparison of promoter methylation of human daughter vs. human parental copies

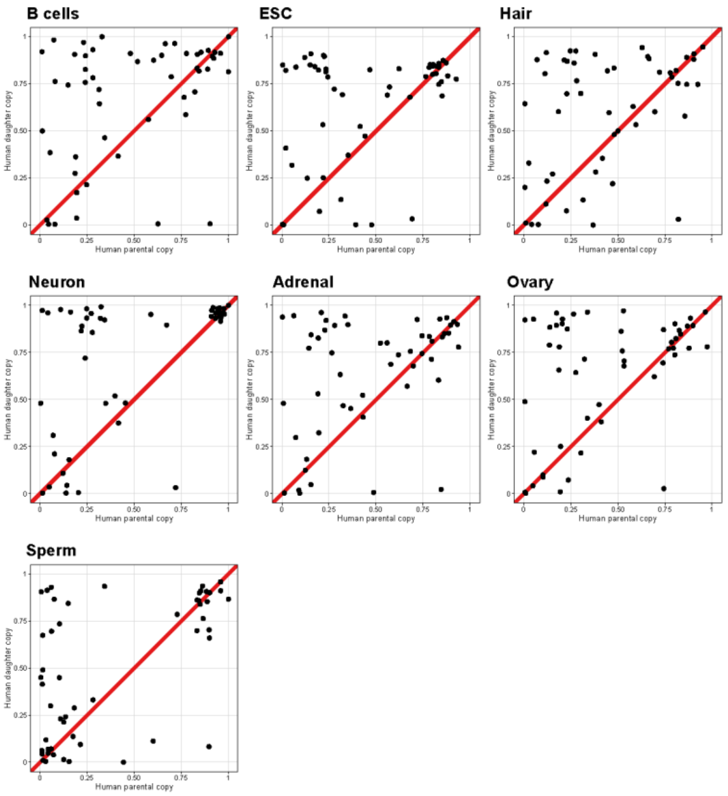

4.6. Comparison of promoter methylation of human daughter vs. human parental copies: violin plots

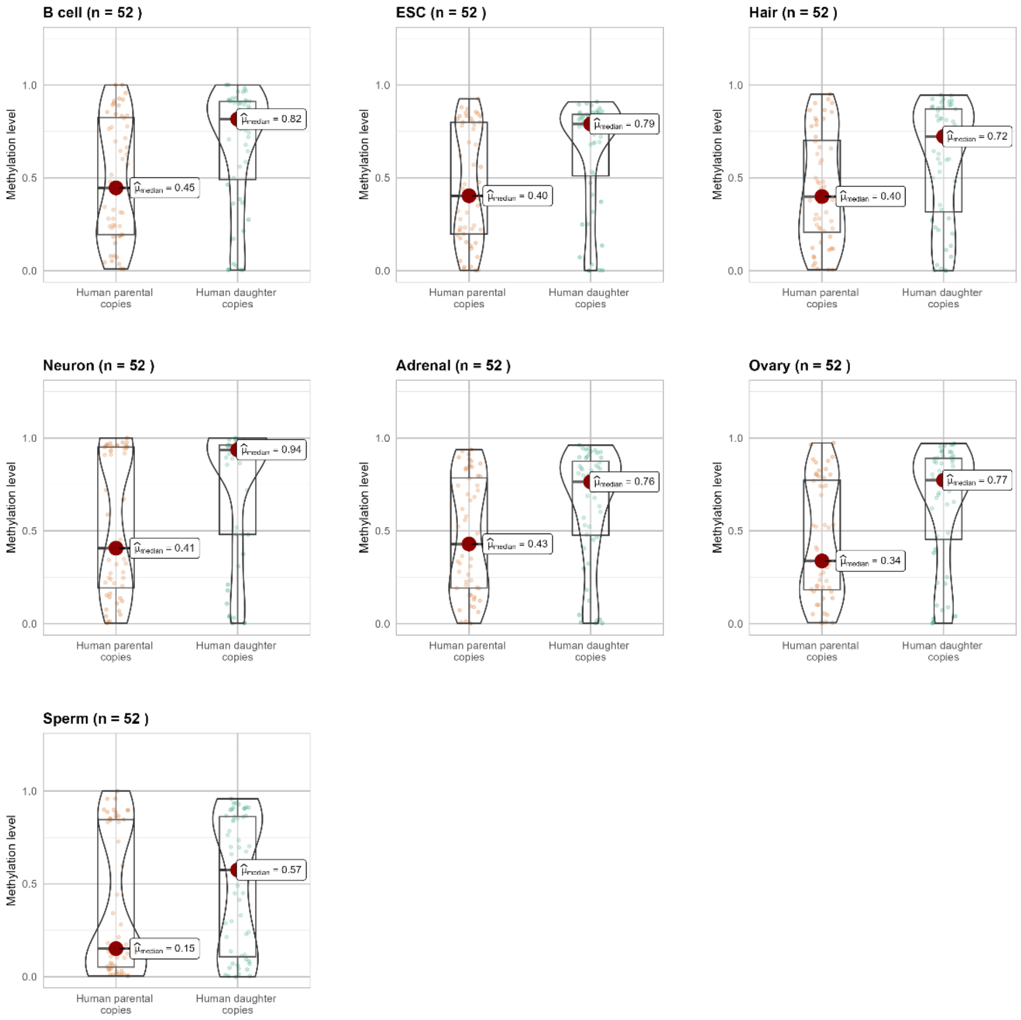

4.7. Comparison of promoter methylation of human daughter copies, human parental copies, and mouse orthologs without retrogenes

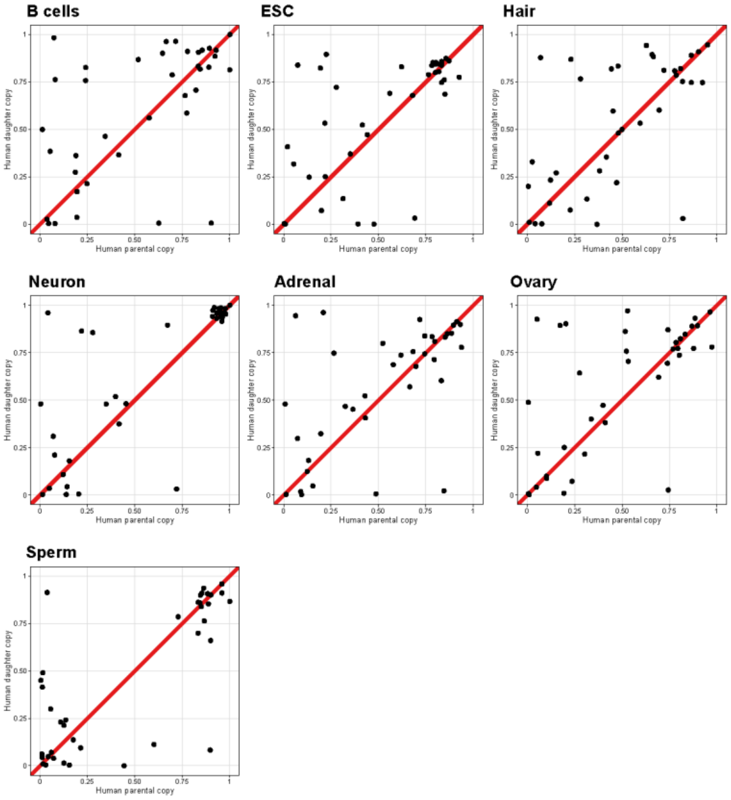

Each dot represents a pair of genes within a trio. For each graph, the red line corresponds to an equal amount of methylation in both genes.

4.8. Comparison of promoter methylation of human daughter copies, human parental copies, and mouse orthologs without retrogenes: violin plots

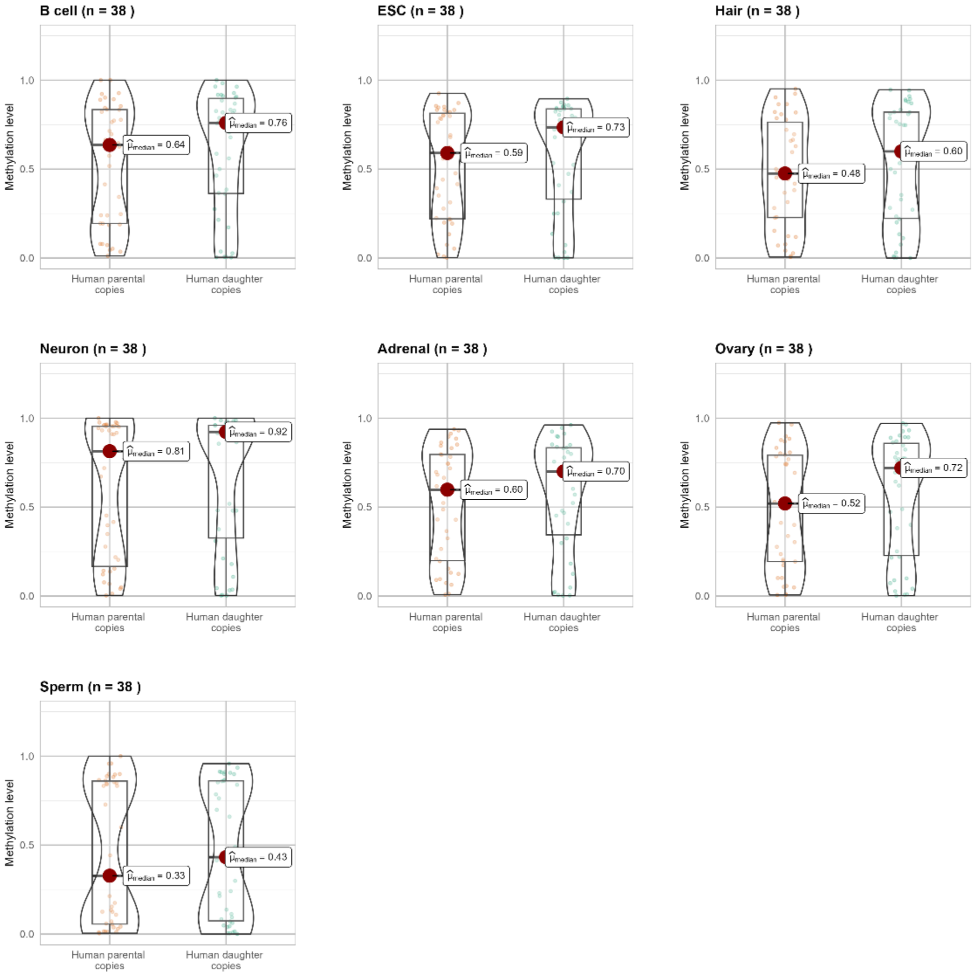

5. Promoter methylation in trios duplicated in mouse but not in human

5.1. Comparison of promoter methylation of mouse daughter copies, mouse parental copies, and human orthologs

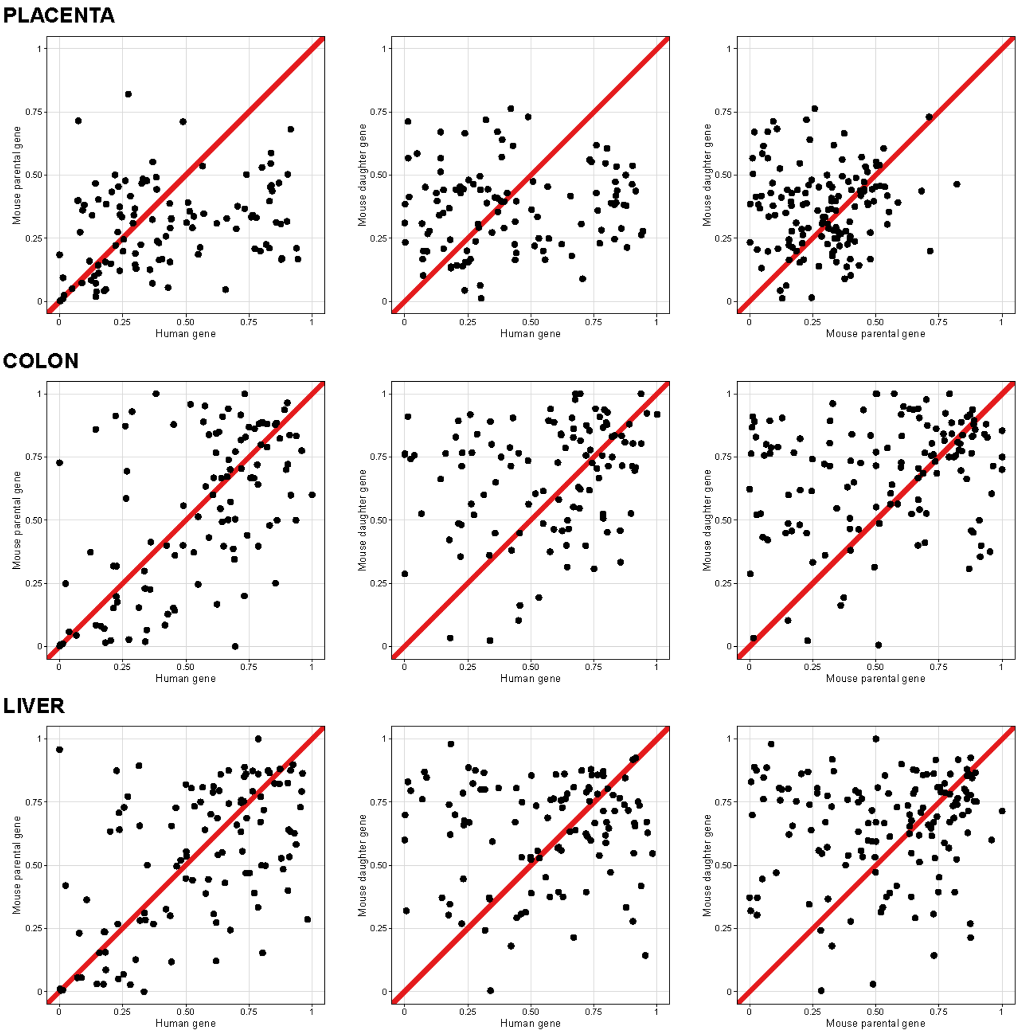

5.2. Comparison of promoter methylation of mouse daughter copies, mouse parental copies, and human orthologs: violin plots

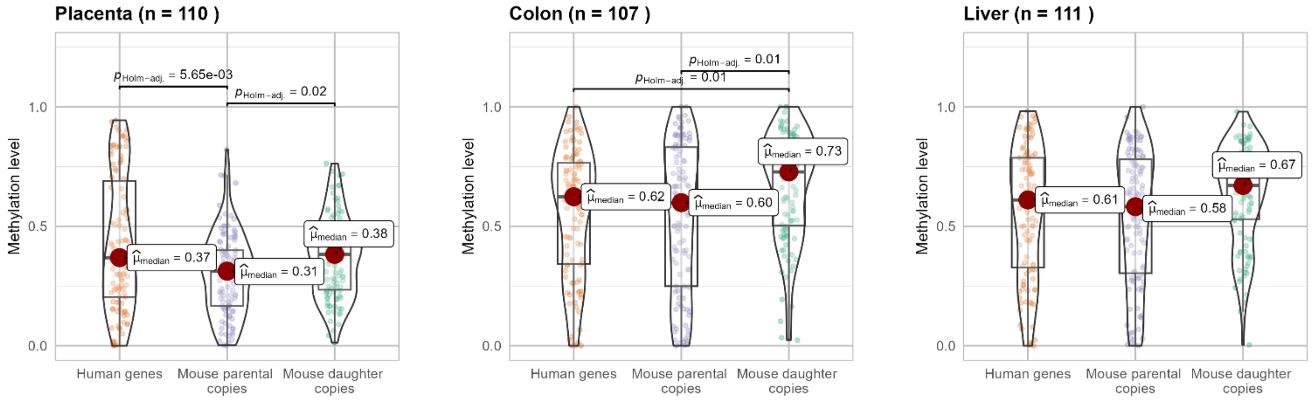

5.3. Comparison of promoter methylation of mouse daughter copies, mouse parental copies, and human orthologs without retrogenes

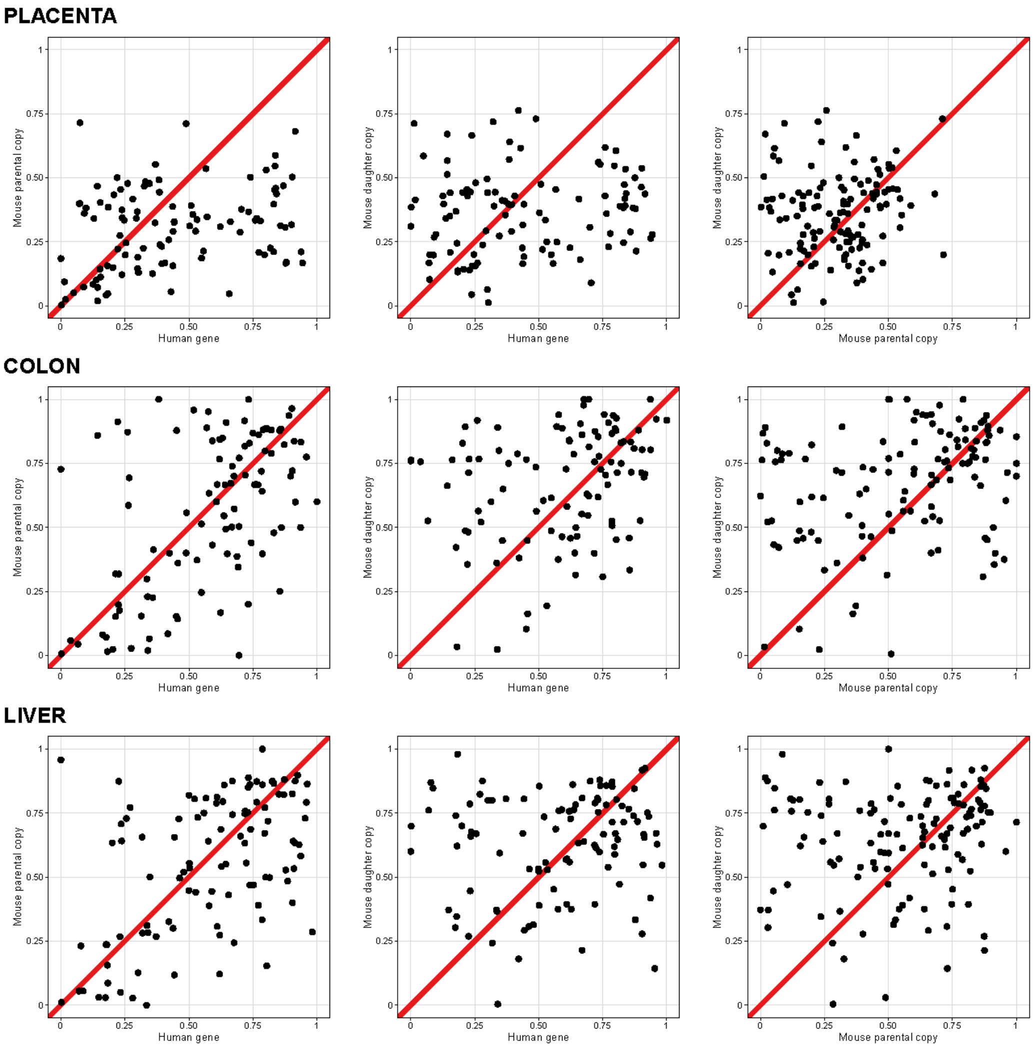

Each dot represents a pair of genes within a trio. For each graph, the red line corresponds to an equal amount of methylation in both genes.

5.4. Comparison of promoter methylation of mouse daughter copies, mouse parental copies, and human orthologs without retrogenes: violin plots

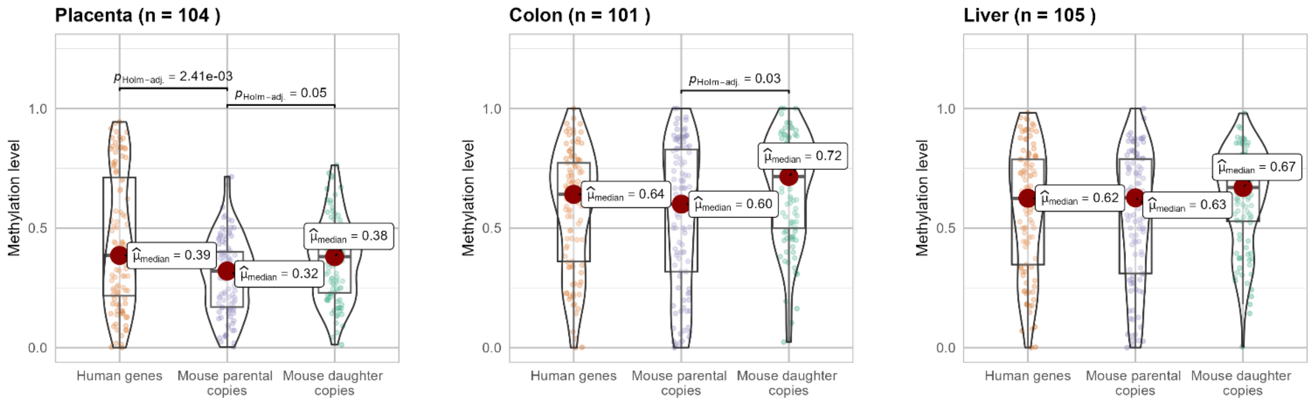

5.5. Comparison of promoter methylation of mouse daughter vs. mouse parental copies

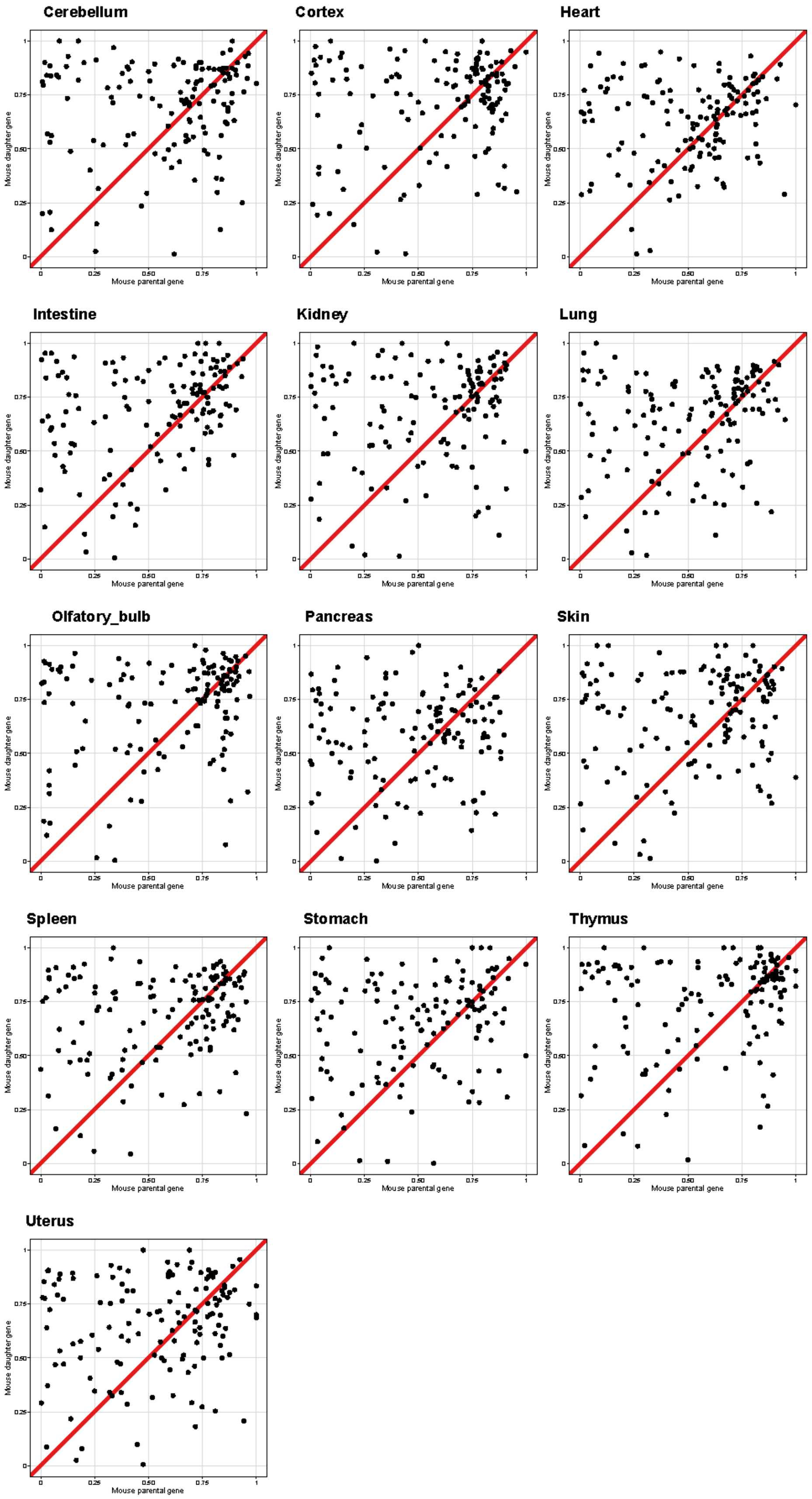

Each dot represents a pair of genes within a trio. For each graph, the red line corresponds to an equal amount of methylation in both genes.

5.6. Comparison of promoter methylation of mouse daughter vs. mouse parental copies: violin plots

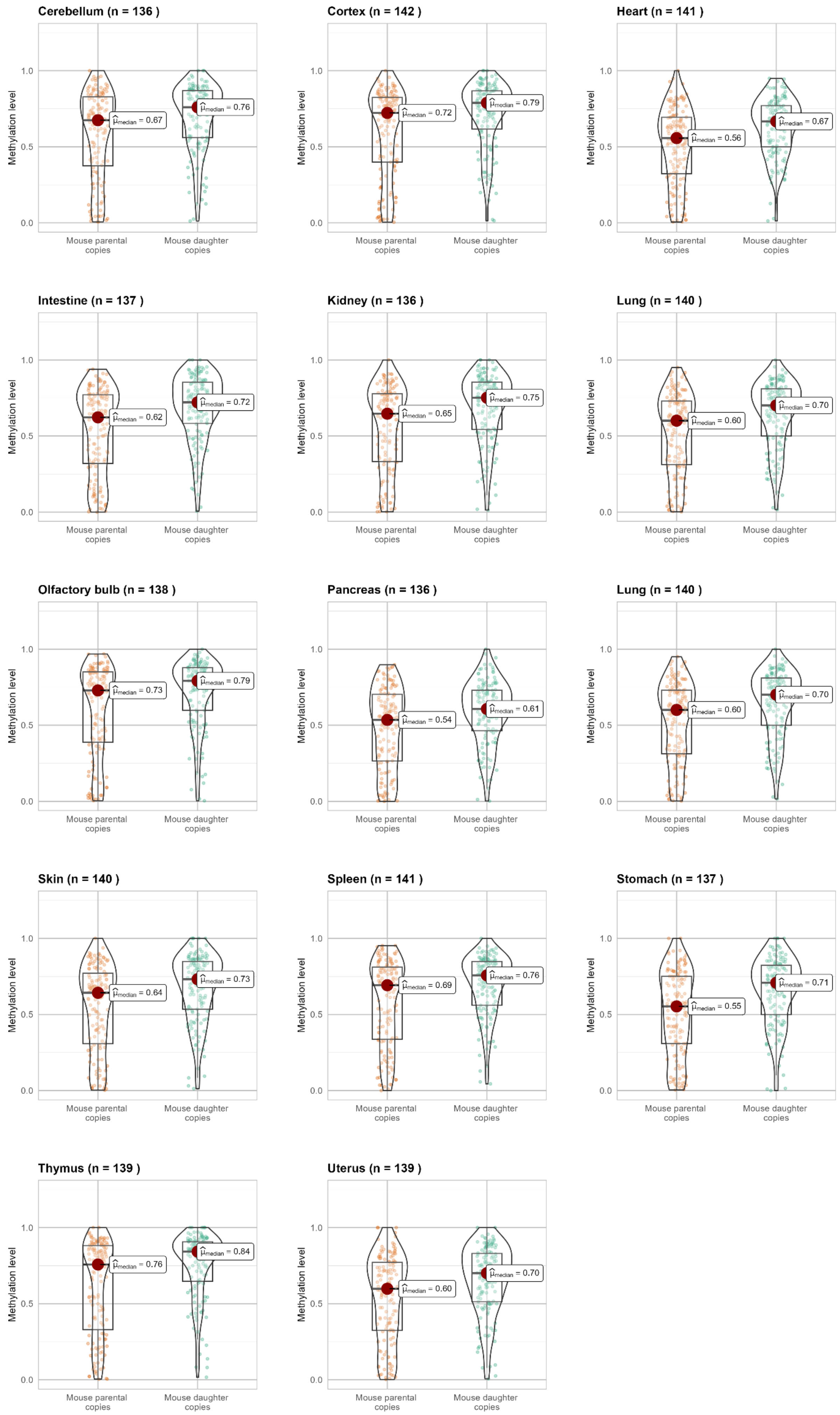

5.7. Comparison of promoter methylation of mouse daughter vs. mouse parental copies without retrogenes

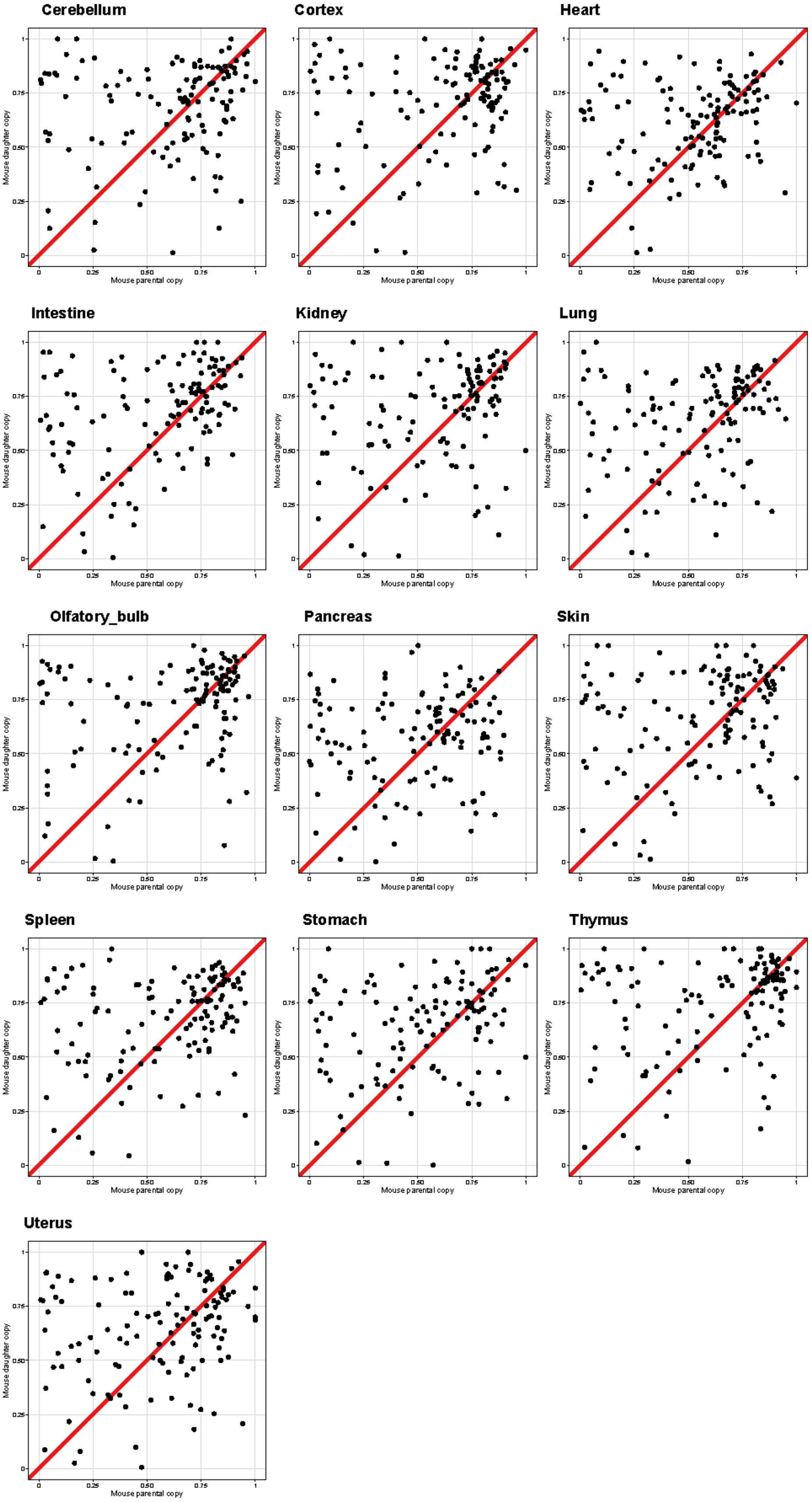

Each dot represents a pair of genes within a trio. For each graph, the red line corresponds to an equal amount of methylation in both genes.

5.8. Comparison of promoter methylation of mouse daughter vs. mouse parental copies without retrogenes: violin plots

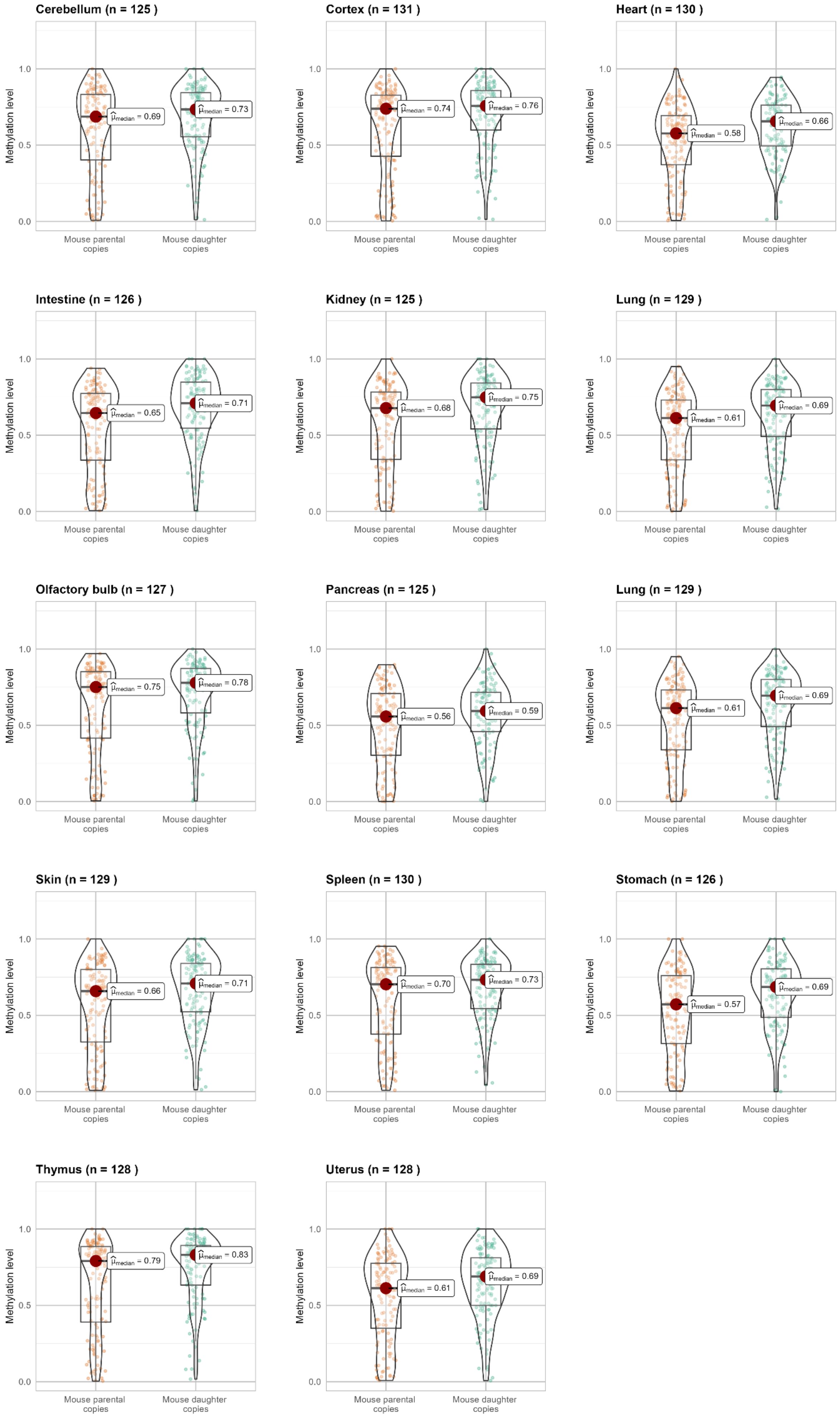

Supplement: msae259_Supplementary_Data [file msae259_supplementary_data.zip › DatasetS1.pdf]
