# Supplementary material for: Asymmetrical Evolution of Promoter Methylation of Mammalian Genes after Duplication
Source: Mol Biol Evol. 2024 Dec 17;41(12):msae259. doi: 10.1093/molbev/msae259 (PMC11683416; doi:10.1093/molbev/msae259)
Supplement: msae259_Supplementary_Data [file msae259_supplementary_data.zip › DatasetS2.pdf]

Supplementary Materials – Dataset S2: UCSC Genome browser screenshots displaying DNA methylation data for specific genes

The screenshots below highlight the differential methylation patterns between specific daughter and ancestral gene copies, emphasizing the hypermethylation observed in the daughter-copy compared to the ancestral-copy. Examples were selected based on genes that align with the overall trends revealed in the analysis. Additionally, orthologous genes in the other species are shown, illustrating consistent methylation trends across species. The tracks were selected from the MethBase2 hub, ensuring that they include methylation data from placenta, liver and colon tissues on the human GRCh38/hg38 and mouse GRCm39/mm39 assemblies. Red boxes mark regions encompassing promoters.

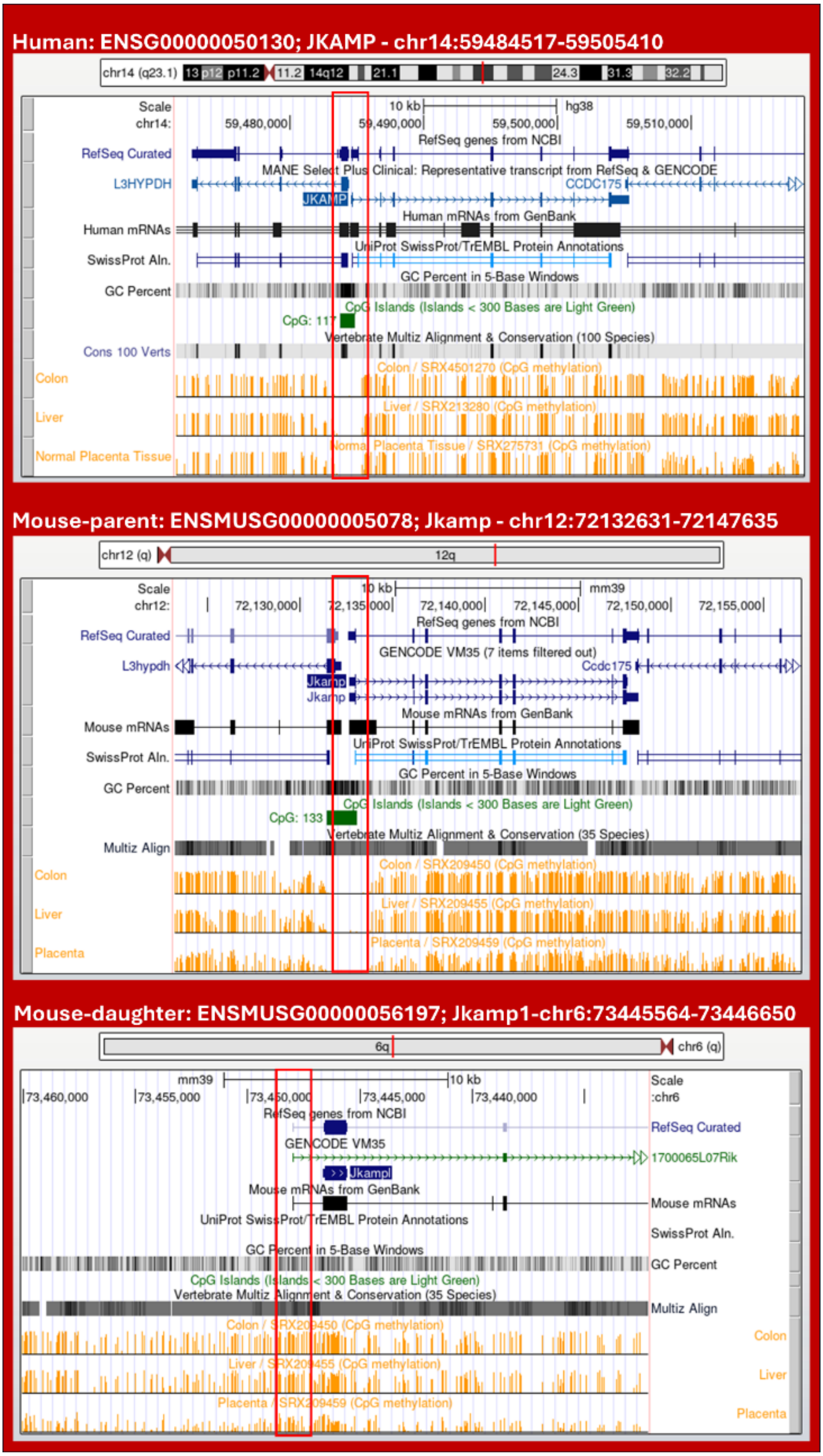

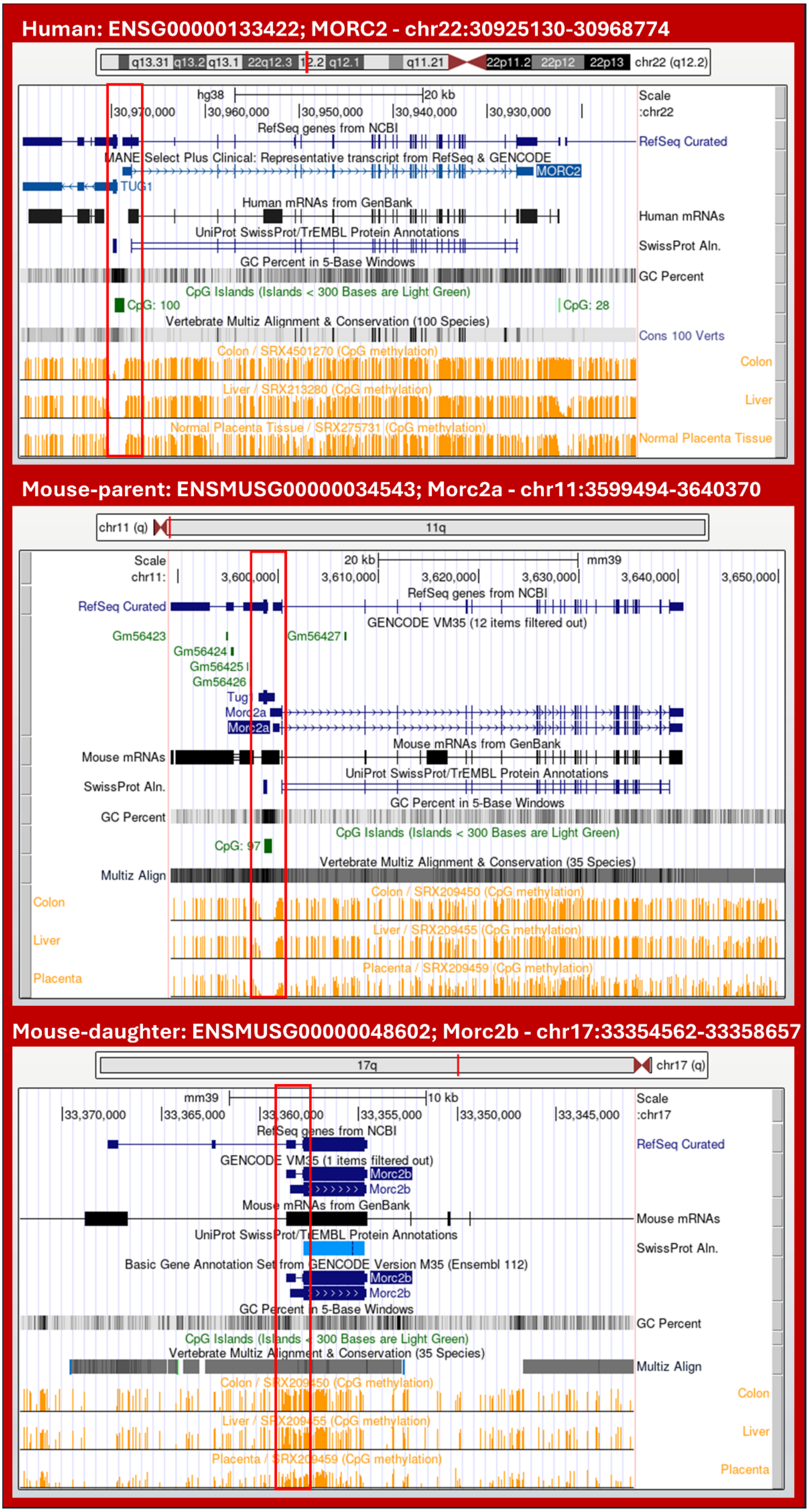
Human: ENSG00000133422; MORC2 - chr22:30925130-30968774
hg38
20 kb
Scale
chr22
RefSeq genes from NCBI
RefSeq Curated
MANE Select Plus Clinical: Representative transcript from RefSeq & GENCODE
MORC2
TUG1
Human mRNAs from GenBank
Human mRNAs
UniProt SwissProt/TrEMBL Protein Annotations
SwissProt Aln.
GC Percent in 5-Base Windows
GC Percent
CpG Islands (Islands < 300 Bases are Light Green)
CpG: 100
CpG: 28
Vertebrate Multiz Alignment & Conservation (100 Species)
Cons 100 Verts
Colon / SRX4501270 (CpG methylation)
Colon
Liver / SRX213280 (CpG methylation)
Liver
Normal Placenta Tissue / SRX275731 (CpG methylation)
Normal Placenta Tissue
Mouse-parent: ENSMUSG00000034543; Morc2a - chr11:3599494-3640370
chr11 (q)
11q
Scale
20 kb
mm39
RefSeq genes from NCBI
RefSeq Curated
GENCODE VM35 (12 items filtered out)
Gm56423
Gm56424
Gm56425
Gm56426
Gm56427
Tug1
Morc2a
Mouse mRNAs from GenBank
Mouse mRNAs
UniProt SwissProt/TrEMBL Protein Annotations
SwissProt Aln.
GC Percent in 5-Base Windows
GC Percent
CpG Islands (Islands < 300 Bases are Light Green)
CpG: 97
Vertebrate Multiz Alignment & Conservation (35 Species)
Multiz Align
Colon / SRX209450 (CpG methylation)
Colon
Liver / SRX209455 (CpG methylation)
Liver
Placenta / SRX209459 (CpG methylation)
Placenta
Mouse-daughter: ENSMUSG00000048602; Morc2b - chr17:33354562-33358657
17q
chr17 (q)
mm39
10 kb
Scale
chr17
RefSeq genes from NCBI
RefSeq Curated
GENCODE VM35 (1 items filtered out)
Morc2b
Mouse mRNAs from GenBank
Mouse mRNAs
UniProt SwissProt/TrEMBL Protein Annotations
SwissProt Aln.
Basic Gene Annotation Set from GENCODE Version M35 (Ensembl 112)
GC Percent in 5-Base Windows
GC Percent
CpG Islands (Islands < 300 Bases are Light Green)
Vertebrate Multiz Alignment & Conservation (35 Species)
Multiz Align
Colon / SRX209450 (CpG methylation)
Colon
Liver / SRX209455 (CpG methylation)
Liver
Placenta / SRX209459 (CpG methylation)
Placenta

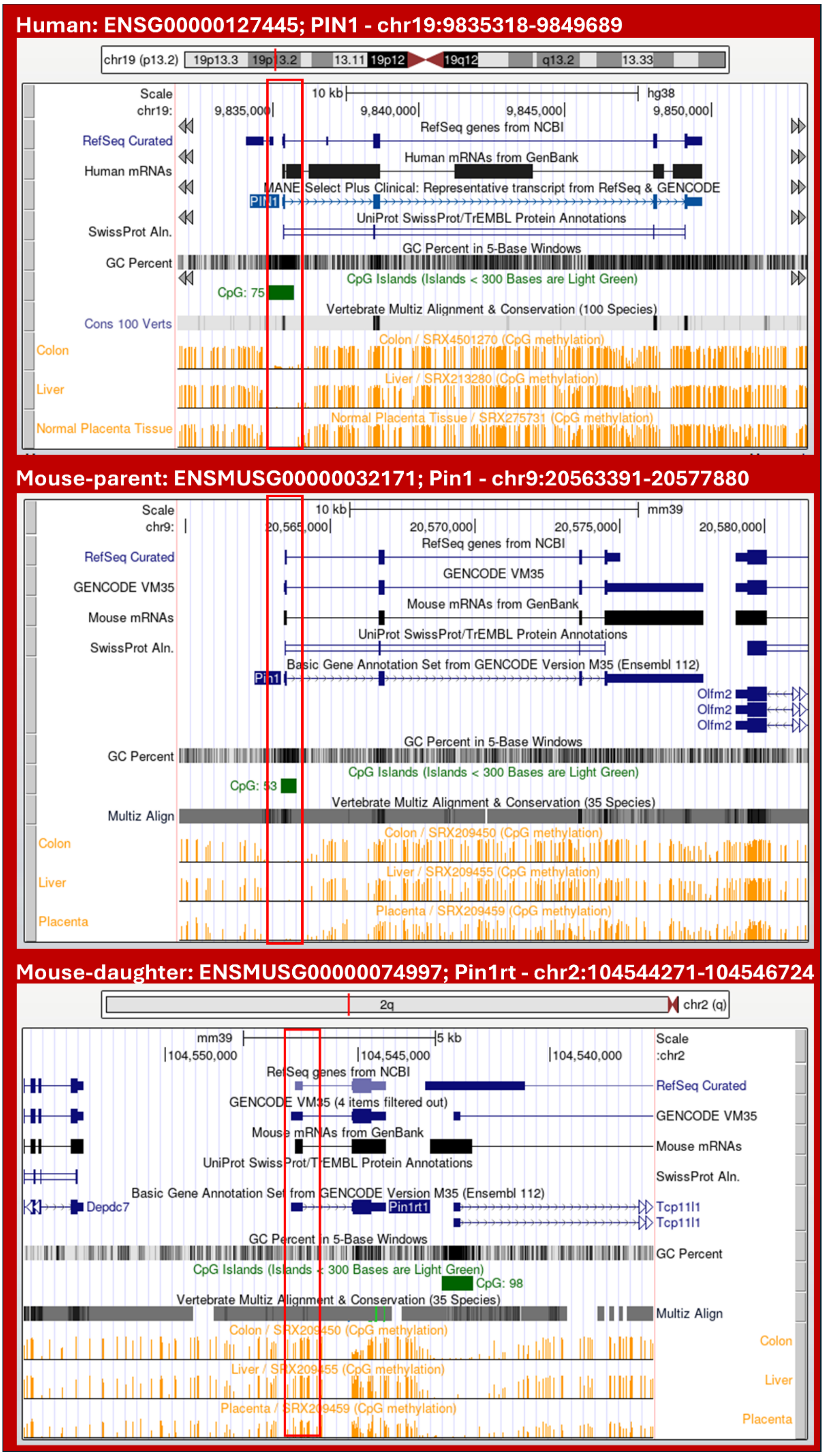
Human: ENSG00000127445; PIN1 - chr19:9835318-9849689
chr19 (p13.2)
Scale
chr19:
10 kb
hg38
9,835,000
9,840,000
9,845,000
9,850,000
RefSeq Curated
RefSeq genes from NCBI
Human mRNAs
Human mRNAs from GenBank
MANE Select Plus Clinical: Representative transcript from RefSeq & GENCODE
PIN1
SwissProt Aln.
UniProt SwissProt/TrEMBL Protein Annotations
GC Percent
GC Percent in 5-Base Windows
CpG Islands (Islands < 300 Bases are Light Green)
CpG: 75
Cons 100 Verts
Vertebrate Multiz Alignment & Conservation (100 Species)
Colon
Colon / SRX4501270 (CpG methylation)
Liver
Liver / SRX213280 (CpG methylation)
Normal Placenta Tissue
Normal Placenta Tissue / SRX275731 (CpG methylation)
Mouse-parent: ENSMUSG00000032171; Pin1 - chr9:20563391-20577880
Scale
chr9:
10 kb
mm39
20,565,000
20,570,000
20,575,000
20,580,000
RefSeq Curated
RefSeq genes from NCBI
GENCODE VM35
Mouse mRNAs
Mouse mRNAs from GenBank
SwissProt Aln.
UniProt SwissProt/TrEMBL Protein Annotations
Basic Gene Annotation Set from GENCODE Version M35 (Ensembl 112)
Pin1
Olfm2
Olfm2
Olfm2
GC Percent
GC Percent in 5-Base Windows
CpG Islands (Islands < 300 Bases are Light Green)
CpG: 53
Multiz Align
Vertebrate Multiz Alignment & Conservation (35 Species)
Colon
Colon / SRX209450 (CpG methylation)
Liver
Liver / SRX209455 (CpG methylation)
Placenta
Placenta / SRX209459 (CpG methylation)
Mouse-daughter: ENSMUSG00000074997; Pin1rt - chr2:104544271-104546724
2q
chr2 (q)
mm39
5 kb
104,550,000
104,545,000
104,540,000
Scale
:chr2
RefSeq genes from NCBI
RefSeq Curated
GENCODE VM35 (4 items filtered out)
GENCODE VM35
Mouse mRNAs from GenBank
Mouse mRNAs
UniProt SwissProt/TrEMBL Protein Annotations
SwissProt Aln.
Basic Gene Annotation Set from GENCODE Version M35 (Ensembl 112)
Depdc7
Pin1rt1
Tcp11l1
Tcp11l1
GC Percent in 5-Base Windows
GC Percent
CpG Islands (Islands < 300 Bases are Light Green)
CpG: 98
Vertebrate Multiz Alignment & Conservation (35 Species)
Multiz Align
Colon / SRX209450 (CpG methylation)
Colon
Liver / SRX209455 (CpG methylation)
Liver
Placenta / SRX209459 (CpG methylation)
Placenta

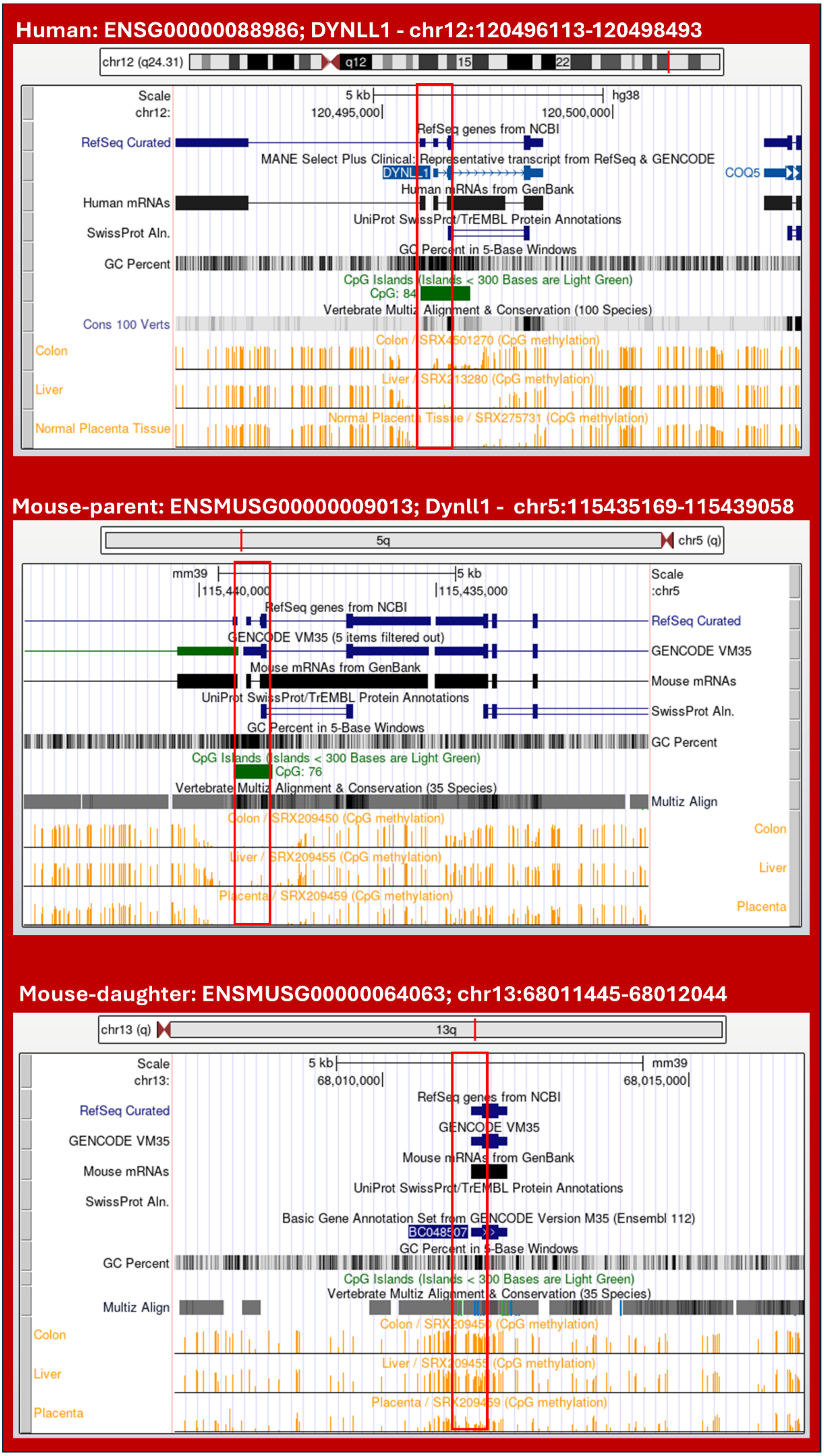
Human: ENSG00000088986; DYNLL1 - chr12:120496113-120498493
chr12 (q24.31)
q12
15
22
Scale
chr12:
5 kb
hg38
120,495,000
120,500,000
RefSeq genes from NCBI
RefSeq Curated
MANE Select Plus Clinical: Representative transcript from RefSeq & GENCODE
DYNLL1
COQ5
Human mRNAs from GenBank
Human mRNAs
UniProt SwissProt/TrEMBL Protein Annotations
SwissProt Aln.
GC Percent in 5-Base Windows
GC Percent
CpG Islands (Islands < 300 Bases are Light Green)
CpG: 84
Vertebrate Multiz Alignment & Conservation (100 Species)
Cons 100 Verts
Colon
Colon / SRX4501270 (CpG methylation)
Liver
Liver / SRX213280 (CpG methylation)
Normal Placenta Tissue
Normal Placenta Tissue / SRX275731 (CpG methylation)
Mouse-parent: ENSMUSG00000009013; Dynll1 - chr5:115435169-115439058
5q
chr5 (q)
mm39
5 kb
115,440,000
115,435,000
Scale
:chr5
RefSeq genes from NCBI
RefSeq Curated
GENCODE VM35 (5 items filtered out)
GENCODE VM35
Mouse mRNAs from GenBank
Mouse mRNAs
UniProt SwissProt/TrEMBL Protein Annotations
SwissProt Aln.
GC Percent in 5-Base Windows
GC Percent
CpG Islands (Islands < 300 Bases are Light Green)
CpG: 76
Vertebrate Multiz Alignment & Conservation (35 Species)
Multiz Align
Colon / SRX209450 (CpG methylation)
Colon
Liver / SRX209455 (CpG methylation)
Liver
Placenta / SRX209459 (CpG methylation)
Placenta
Mouse-daughter: ENSMUSG00000064063; chr13:68011445-68012044
chr13 (q)
13q
Scale
chr13:
5 kb
mm39
68,010,000
68,015,000
RefSeq genes from NCBI
RefSeq Curated
GENCODE VM35
GENCODE VM35
Mouse mRNAs from GenBank
Mouse mRNAs
UniProt SwissProt/TrEMBL Protein Annotations
SwissProt Aln.
Basic Gene Annotation Set from GENCODE Version M35 (Ensembl 112)
BC048507
GC Percent in 5-Base Windows
GC Percent
CpG Islands (Islands < 300 Bases are Light Green)
Vertebrate Multiz Alignment & Conservation (35 Species)
Multiz Align
Colon
Colon / SRX209450 (CpG methylation)
Liver
Liver / SRX209455 (CpG methylation)
Placenta
Placenta / SRX209459 (CpG methylation)

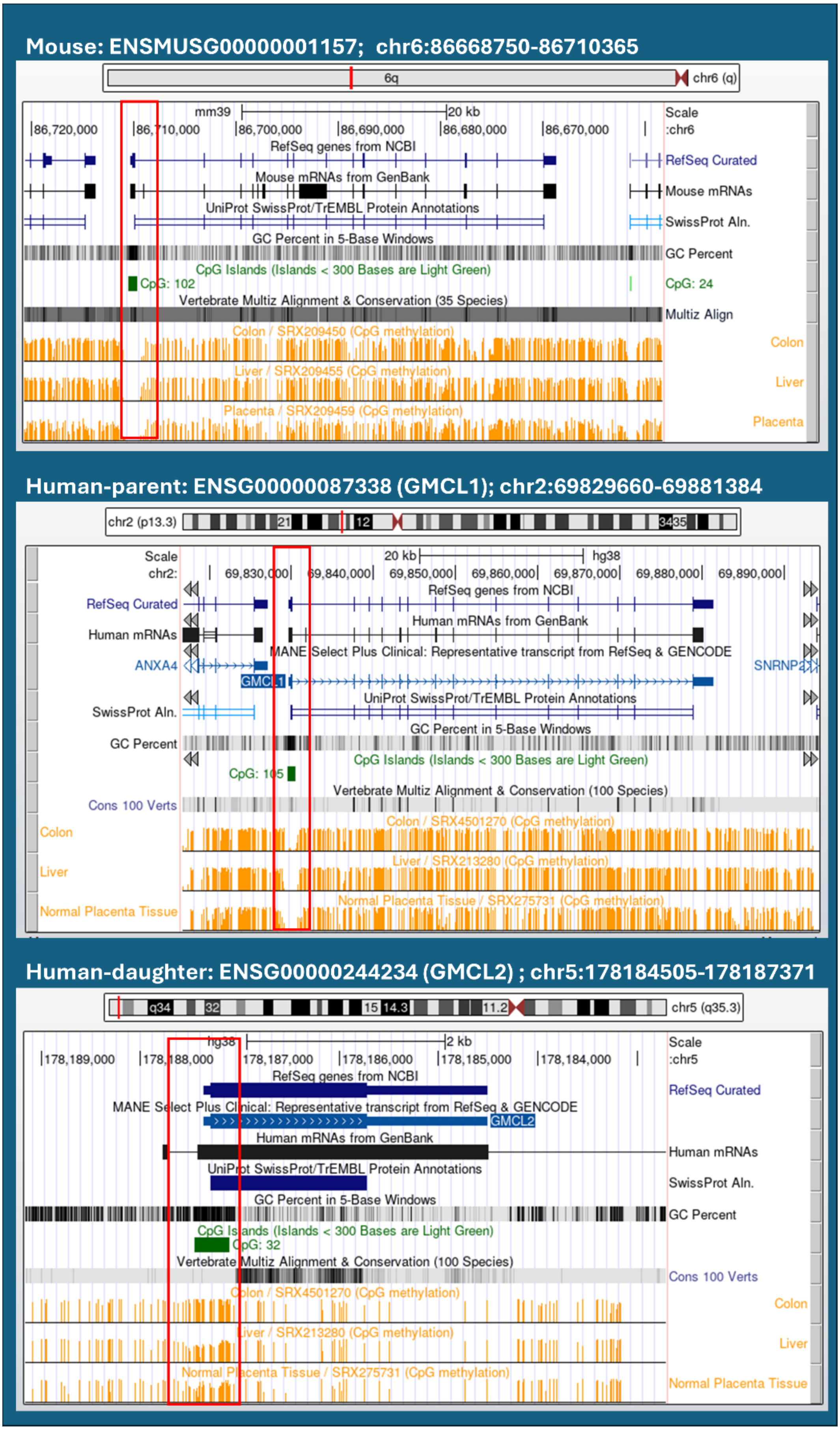
Mouse: ENSMUSG00000001157; chr6:86668750-86710365
6q
chr6 (q)
mm39
20 kb
Scale
:chr6
86,720,000
86,710,000
86,700,000
86,690,000
86,680,000
86,670,000
RefSeq genes from NCBI
RefSeq Curated
Mouse mRNAs from GenBank
Mouse mRNAs
UniProt SwissProt/TrEMBL Protein Annotations
SwissProt Aln.
GC Percent in 5-Base Windows
GC Percent
CpG Islands (Islands < 300 Bases are Light Green)
CpG: 102
CpG: 24
Vertebrate Multiz Alignment & Conservation (35 Species)
Multiz Align
Colon / SRX209450 (CpG methylation)
Colon
Liver / SRX209455 (CpG methylation)
Liver
Placenta / SRX209459 (CpG methylation)
Placenta
Human-parent: ENSG00000087338 (GMCL1); chr2:69829660-69881384
chr2 (p13.3)
Scale
chr2:
20 kb
hg38
69,830,000
69,840,000
69,850,000
69,860,000
69,870,000
69,880,000
69,890,000
RefSeq genes from NCBI
RefSeq Curated
Human mRNAs from GenBank
Human mRNAs
MANE Select Plus Clinical: Representative transcript from RefSeq & GENCODE
ANXA4
GMCL1
SNRNP27
UniProt SwissProt/TrEMBL Protein Annotations
SwissProt Aln.
GC Percent in 5-Base Windows
GC Percent
CpG Islands (Islands < 300 Bases are Light Green)
CpG: 105
Vertebrate Multiz Alignment & Conservation (100 Species)
Cons 100 Verts
Colon / SRX4501270 (CpG methylation)
Colon
Liver / SRX213280 (CpG methylation)
Liver
Normal Placenta Tissue / SRX275731 (CpG methylation)
Normal Placenta Tissue
Human-daughter: ENSG00000244234 (GMCL2) ; chr5:178184505-178187371
chr5 (q35.3)
hg38
2 kb
Scale
:chr5
178,189,000
178,188,000
178,187,000
178,186,000
178,185,000
178,184,000
RefSeq genes from NCBI
RefSeq Curated
MANE Select Plus Clinical: Representative transcript from RefSeq & GENCODE
GMCL2
Human mRNAs from GenBank
Human mRNAs
UniProt SwissProt/TrEMBL Protein Annotations
SwissProt Aln.
GC Percent in 5-Base Windows
GC Percent
CpG Islands (Islands < 300 Bases are Light Green)
CpG: 32
Vertebrate Multiz Alignment & Conservation (100 Species)
Cons 100 Verts
Colon / SRX4501270 (CpG methylation)
Colon
Liver / SRX213280 (CpG methylation)
Liver
Normal Placenta Tissue / SRX275731 (CpG methylation)
Normal Placenta Tissue

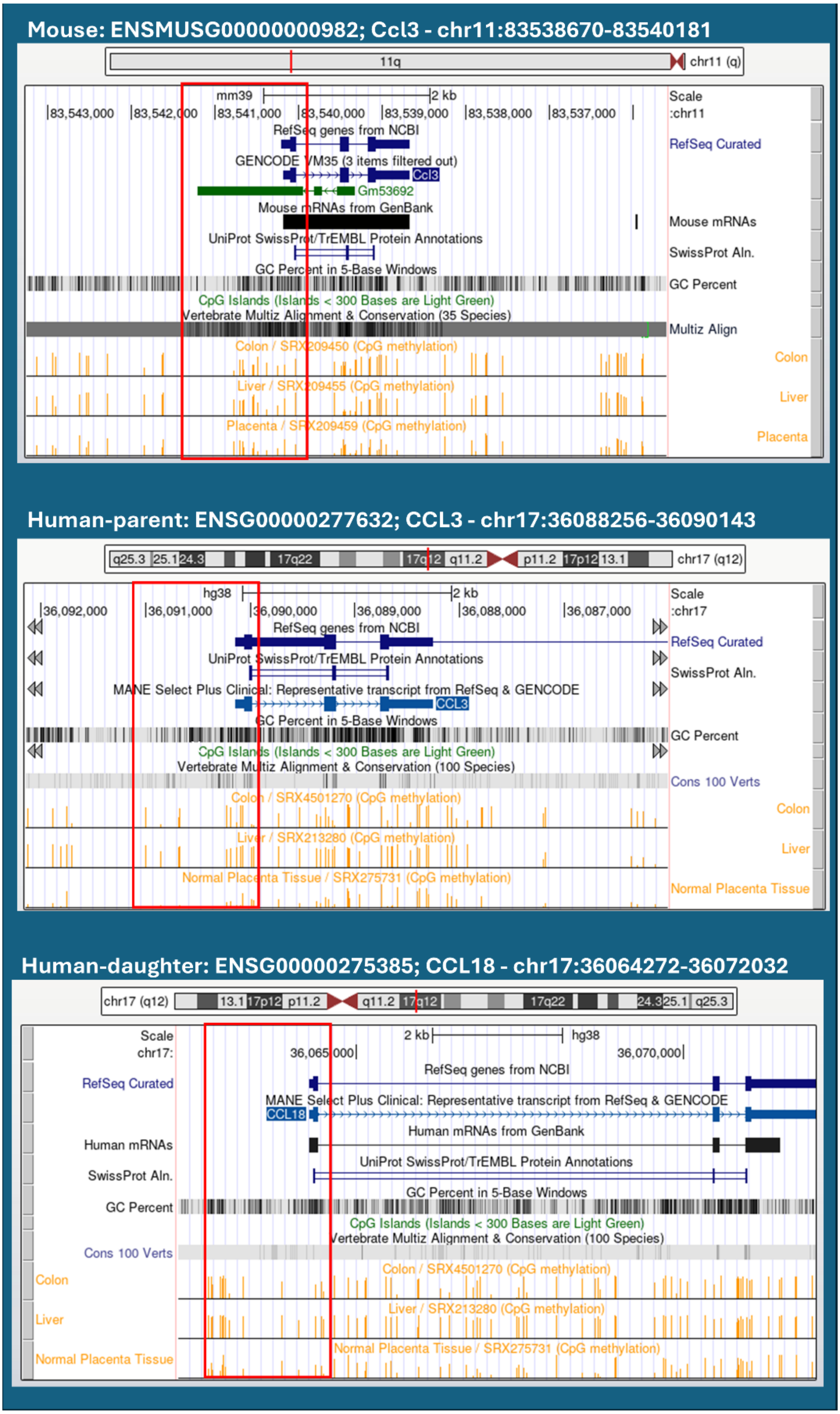
Mouse: ENSMUSG00000000982; Ccl3 - chr11:83538670-83540181
11q
chr11 (q)
mm39
2 kb
83,543,000
83,542,000
83,541,000
83,540,000
83,539,000
83,538,000
83,537,000
Scale
chr11
RefSeq genes from NCBI
RefSeq Curated
GENCODE VM35 (3 items filtered out)
Ccl3
Gm53692
Mouse mRNAs from GenBank
Mouse mRNAs
UniProt SwissProt/TrEMBL Protein Annotations
SwissProt Aln.
GC Percent in 5-Base Windows
GC Percent
CpG Islands (Islands < 300 Bases are Light Green)
Vertebrate Multiz Alignment & Conservation (35 Species)
Multiz Align
Colon / SRX209450 (CpG methylation)
Colon
Liver / SRX209455 (CpG methylation)
Liver
Placenta / SRX209459 (CpG methylation)
Placenta
Human-parent: ENSG00000277632; CCL3 - chr17:36088256-36090143
q25.3
25.1
24.3
17q22
17q12
q11.2
p11.2
17p12
13.1
chr17 (q12)
hg38
2 kb
36,092,000
36,091,000
36,090,000
36,089,000
36,088,000
36,087,000
Scale
chr17
RefSeq genes from NCBI
RefSeq Curated
UniProt SwissProt/TrEMBL Protein Annotations
SwissProt Aln.
MANE Select Plus Clinical: Representative transcript from RefSeq & GENCODE
CCL3
GC Percent in 5-Base Windows
GC Percent
CpG Islands (Islands < 300 Bases are Light Green)
Vertebrate Multiz Alignment & Conservation (100 Species)
Cons 100 Verts
Colon / SRX4501270 (CpG methylation)
Colon
Liver / SRX213280 (CpG methylation)
Liver
Normal Placenta Tissue / SRX275731 (CpG methylation)
Normal Placenta Tissue
Human-daughter: ENSG00000275385; CCL18 - chr17:36064272-36072032
chr17 (q12)
13.1
17p12
p11.2
q11.2
17q12
17q22
24.3
25.1
q25.3
Scale
chr17:
2 kb
hg38
36,065,000
36,070,000
RefSeq genes from NCBI
RefSeq Curated
MANE Select Plus Clinical: Representative transcript from RefSeq & GENCODE
CCL18
Human mRNAs from GenBank
Human mRNAs
UniProt SwissProt/TrEMBL Protein Annotations
SwissProt Aln.
GC Percent in 5-Base Windows
GC Percent
CpG Islands (Islands < 300 Bases are Light Green)
Vertebrate Multiz Alignment & Conservation (100 Species)
Cons 100 Verts
Colon / SRX4501270 (CpG methylation)
Colon
Liver / SRX213280 (CpG methylation)
Liver
Normal Placenta Tissue / SRX275731 (CpG methylation)
Normal Placenta Tissue

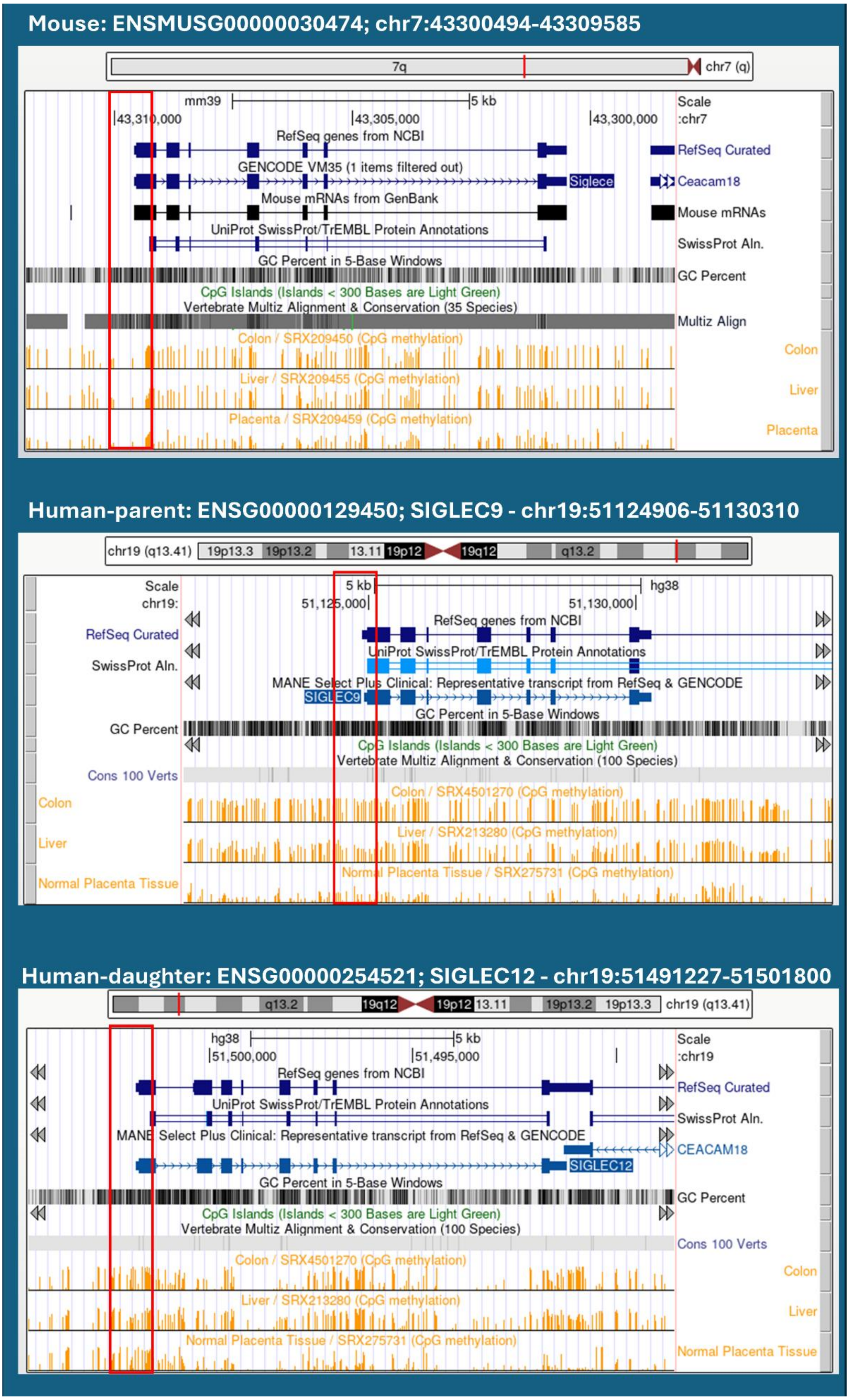
Mouse: ENSMUSG00000030474; chr7:43300494-43309585
7q
chr7 (q)
mm39
5 kb
Scale
:chr7
43,310,000
43,305,000
43,300,000
RefSeq genes from NCBI
RefSeq Curated
GENCODE VM35 (1 items filtered out)
Siglece
Ceacam18
Mouse mRNAs from GenBank
Mouse mRNAs
UniProt SwissProt/TrEMBL Protein Annotations
SwissProt Aln.
GC Percent in 5-Base Windows
GC Percent
CpG Islands (Islands < 300 Bases are Light Green)
Vertebrate Multiz Alignment & Conservation (35 Species)
Multiz Align
Colon / SRX209450 (CpG methylation)
Colon
Liver / SRX209455 (CpG methylation)
Liver
Placenta / SRX209459 (CpG methylation)
Placenta
Human-parent: ENSG00000129450; SIGLEC9 - chr19:51124906-51130310
chr19 (q13.41)
19p13.3
19p13.2
13.11
19p12
19q12
q13.2
Scale
chr19:
5 kb
hg38
51,125,000
51,130,000
RefSeq genes from NCBI
RefSeq Curated
UniProt SwissProt/TrEMBL Protein Annotations
SwissProt Aln.
MANE Select Plus Clinical: Representative transcript from RefSeq & GENCODE
SIGLEC9
GC Percent in 5-Base Windows
GC Percent
CpG Islands (Islands < 300 Bases are Light Green)
Vertebrate Multiz Alignment & Conservation (100 Species)
Cons 100 Verts
Colon / SRX4501270 (CpG methylation)
Colon
Liver / SRX213280 (CpG methylation)
Liver
Normal Placenta Tissue / SRX275731 (CpG methylation)
Normal Placenta Tissue
Human-daughter: ENSG00000254521; SIGLEC12 - chr19:51491227-51501800
q13.2
19q12
19p12
13.11
19p13.2
19p13.3
chr19 (q13.41)
hg38
5 kb
Scale
:chr19
51,500,000
51,495,000
RefSeq genes from NCBI
RefSeq Curated
UniProt SwissProt/TrEMBL Protein Annotations
SwissProt Aln.
MANE Select Plus Clinical: Representative transcript from RefSeq & GENCODE
CEACAM18
SIGLEC12
GC Percent in 5-Base Windows
GC Percent
CpG Islands (Islands < 300 Bases are Light Green)
Vertebrate Multiz Alignment & Conservation (100 Species)
Cons 100 Verts
Colon / SRX4501270 (CpG methylation)
Colon
Liver / SRX213280 (CpG methylation)
Liver
Normal Placenta Tissue / SRX275731 (CpG methylation)
Normal Placenta Tissue
