# Supplementary material for: Asymmetrical Evolution of Promoter Methylation of Mammalian Genes after Duplication
Source: Mol Biol Evol. 2024 Dec 17;41(12):msae259. doi: 10.1093/molbev/msae259 (PMC11683416; doi:10.1093/molbev/msae259)

Supplementary Materials – Dataset S3: Gene body methylation analyses

CONTENTS

1. Comparison of gene body methylation levels among singletons and duplicates 2

1.1. Human genes 2

1.2. Mouse genes 3

2. Comparison of gene body methylation levels among the different kinds of orthologs 5

2.1. Human genes 5

2.2. Mouse genes 6

3. Correlation of gene body methylation levels of recent duplicates with the Gene Order Conservation (GOC) scores 8

3.1. Human genes 8

3.2. Mouse genes 11

4. Gene body methylation in trios duplicated in human but not in mouse 17

4.1. Comparison of gene body methylation of human daughter copies, human parental copies, and mouse orthologs 17

4.2. Comparison of gene body methylation of human daughter copies, human parental copies, and mouse orthologs: violin plots 17

4.3. Comparison of gene body methylation of human daughter copies, human parental copies, and mouse orthologs without retrogenes 18

4.4. Comparison of gene body methylation of human daughter copies, human parental copies, and mouse orthologs without retrogenes: violin plots 18

4.5. Comparison of gene body methylation of human daughter vs. human parental copies 19

4.6. Comparison of gene body methylation of human daughter vs. human parental copies: violin plots 19

4.7. Comparison of gene body methylation of human daughter vs. human parental copies without retrogenes 20

4.8. Comparison of gene body methylation of human daughter vs. human parental copies without retrogenes: violin plots 20

5. Gene body methylation in trios duplicated in mouse but not in human 21

5.1. Comparison of gene body methylation of mouse daughter copies, mouse parental copies, and human orthologs 21

5.2. Comparison of gene body methylation of mouse daughter copies, mouse parental copies, and human orthologs: violin plots 21

5.3. Comparison of gene body methylation of mouse daughter copies, mouse parental copies, and human orthologs without retrogenes 22

5.4. Comparison of gene body methylation of mouse daughter copies, mouse parental copies, and human orthologs without retrogenes: violin plots 22

5.5. Comparison of gene body methylation of mouse daughter vs. mouse parental copies 23

5.6. Comparison of gene body methylation of mouse daughter vs. mouse parental copies: violin plots 24

5.7. Comparison of gene body methylation of mouse daughter vs. mouse parental copies without retrogenes 25

5.8. Comparison of gene body methylation of mouse daughter vs. mouse parental copies without retrogenes: violin plots 26

1. Comparison of gene body methylation levels among singletons and duplicates

1.1. Human genes

Below are the representations of comparison of gene body methylation levels singletons vs. duplicates in the10 human tissues analyzed. In each plot, each dot represents a gene. The horizontal lines indicate significant differences based on two-sided Dunn's pairwise tests with Holm correction for multiple comparisons. Comparisons were deemed significant if corrected p-values were lower than 0.05.

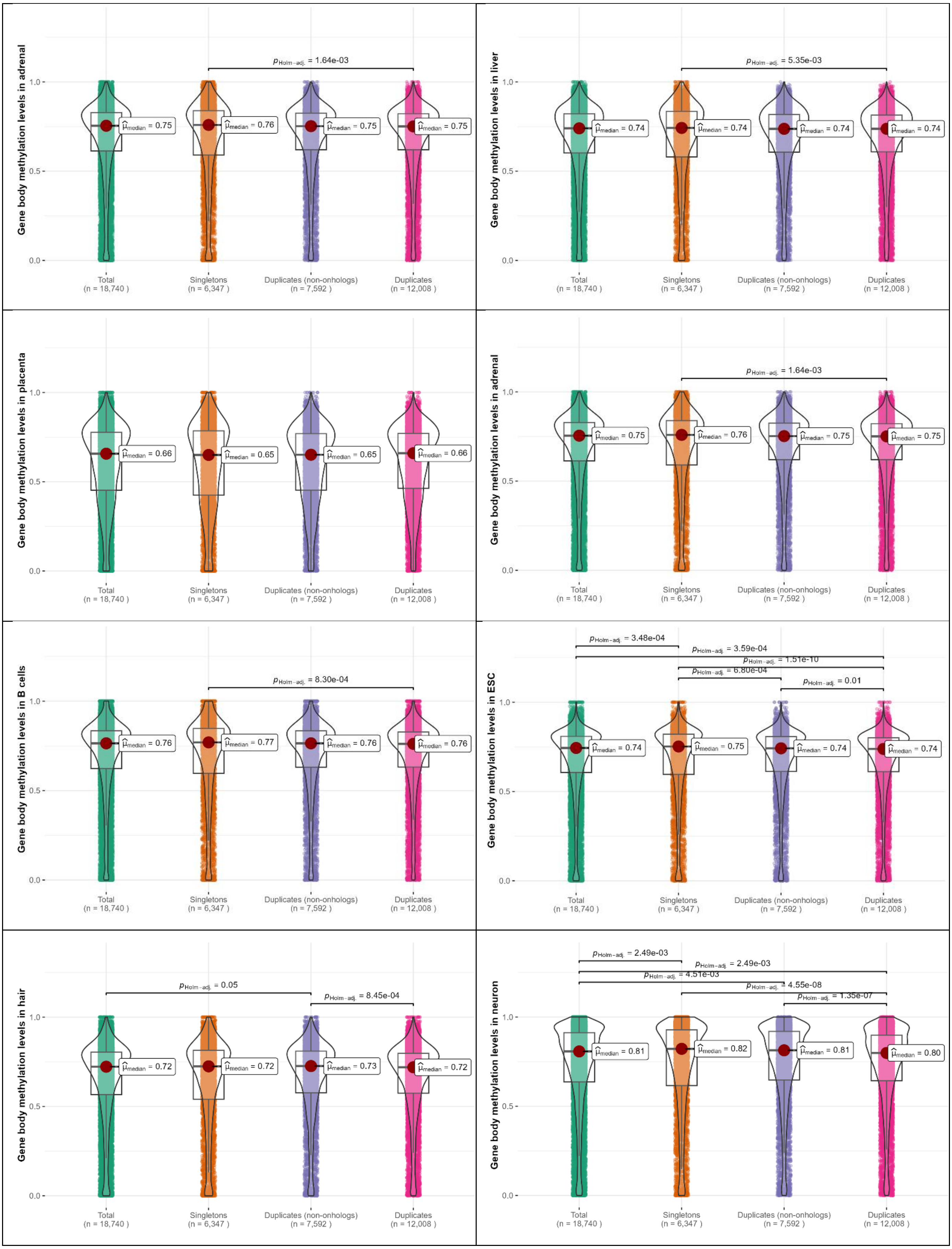

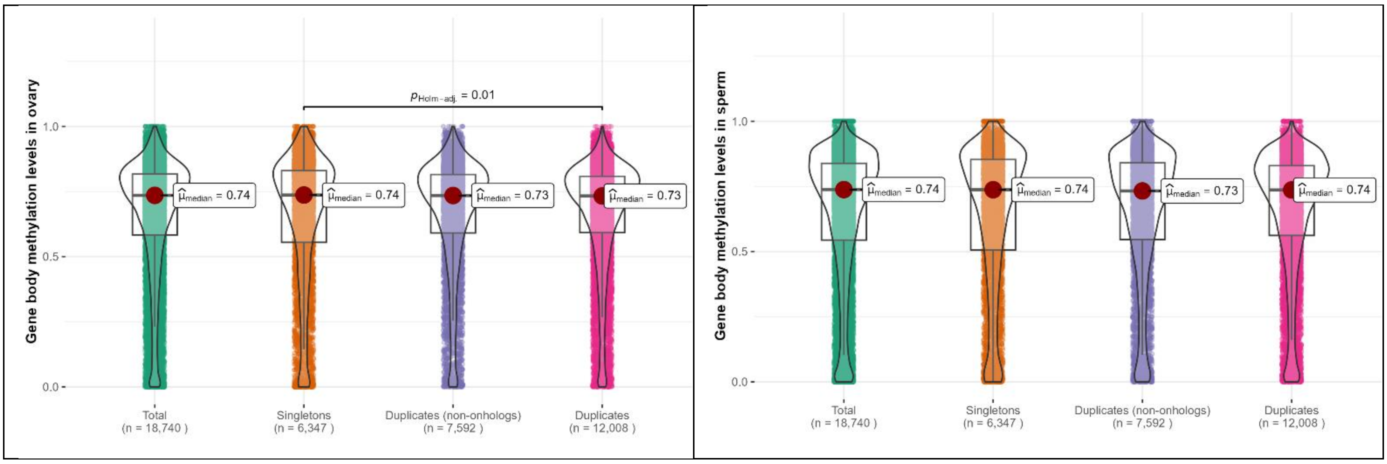

1.2. Mouse genes

Below are the representations of comparison of gene body methylation levels singletons vs. duplicates in the 16 mouse tissues analyzed. In each plot, each dot represents a gene. The horizontal lines indicate significant differences based on two-sided Dunn's pairwise tests with Holm correction for multiple comparisons. Comparisons were deemed significant if corrected p-values were lower than 0.05.

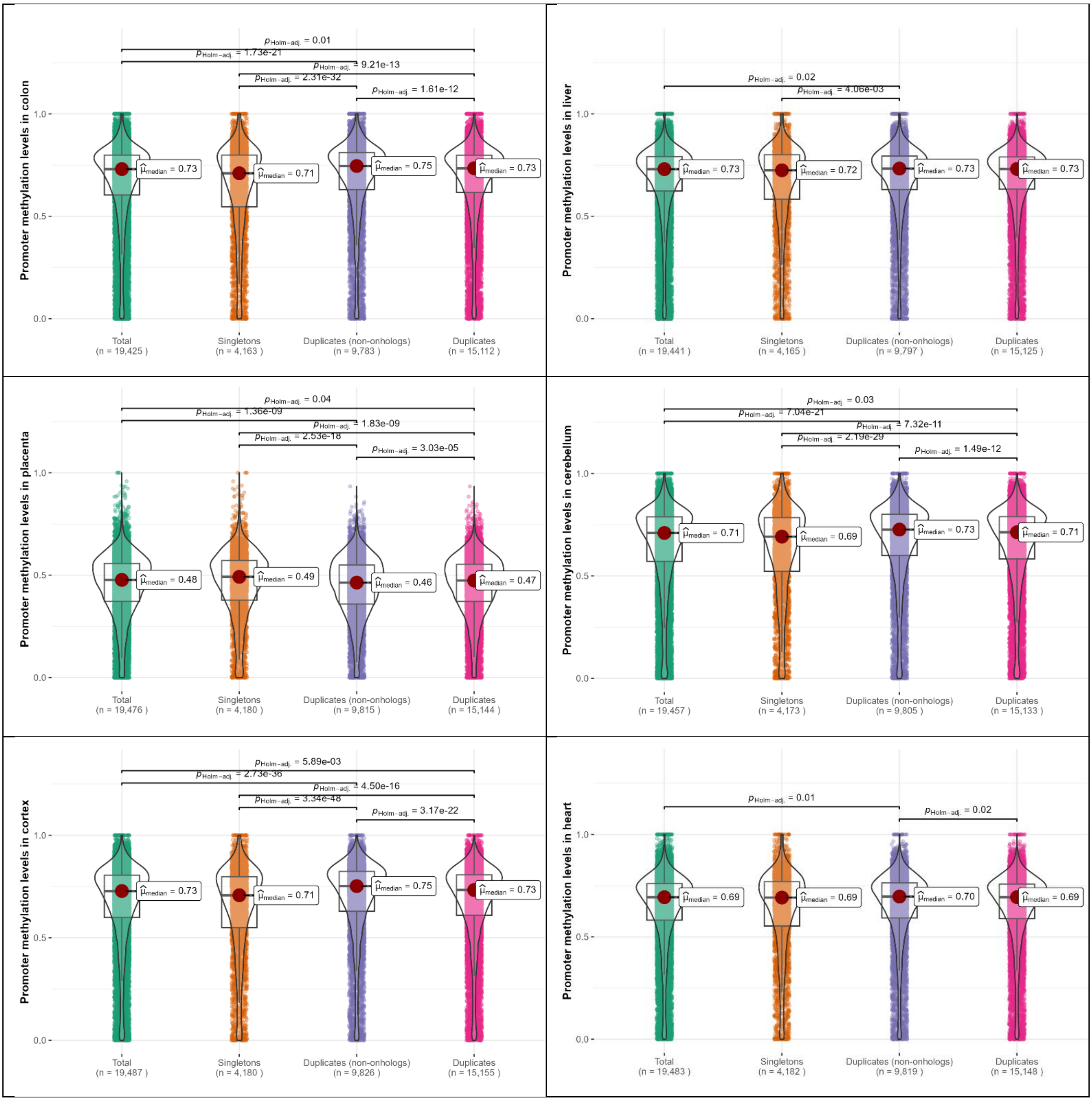

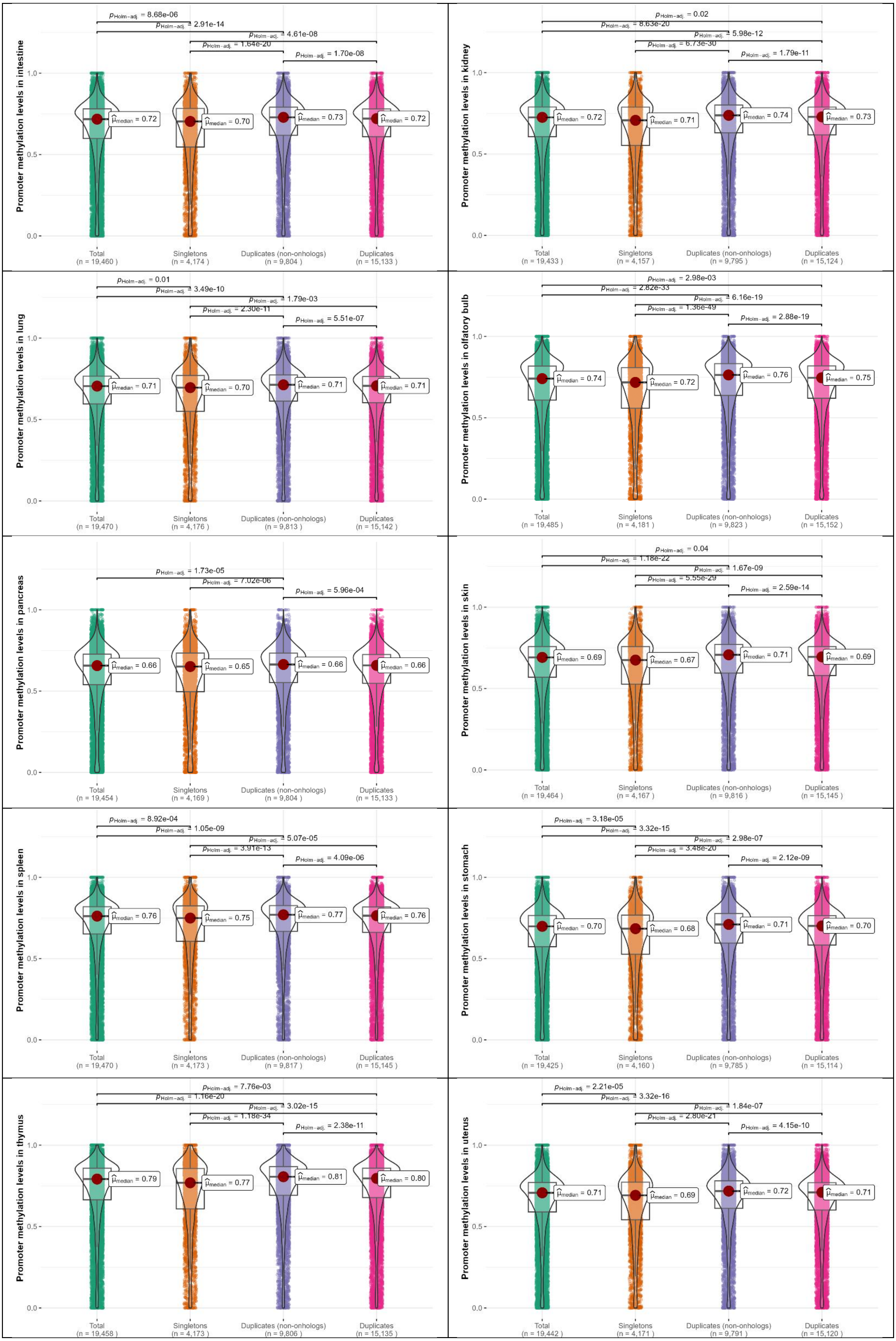

Promoter methylation levels in intestine
pHolm-adj. = 8.68e-06
pHolm-adj. = 2.91e-14
pHolm-adj. = 4.61e-08
pHolm-adj. = 1.64e-20
pHolm-adj. = 1.70e-08
μ̂median = 0.72
μ̂median = 0.70
μ̂median = 0.73
μ̂median = 0.72
Total (n = 19,460)
Singletons (n = 4,174)
Duplicates (non-onhologs) (n = 9,804)
Duplicates (n = 15,133)
Promoter methylation levels in kidney
pHolm-adj. = 0.02
pHolm-adj. = 8.63e-20
pHolm-adj. = 5.98e-12
pHolm-adj. = 6.73e-30
pHolm-adj. = 1.79e-11
μ̂median = 0.72
μ̂median = 0.71
μ̂median = 0.74
μ̂median = 0.73
Total (n = 19,433)
Singletons (n = 4,157)
Duplicates (non-onhologs) (n = 9,795)
Duplicates (n = 15,124)
Promoter methylation levels in lung
pHolm-adj. = 0.01
pHolm-adj. = 3.49e-10
pHolm-adj. = 1.79e-03
pHolm-adj. = 2.30e-11
pHolm-adj. = 5.51e-07
μ̂median = 0.71
μ̂median = 0.70
μ̂median = 0.71
μ̂median = 0.71
Total (n = 19,470)
Singletons (n = 4,176)
Duplicates (non-onhologs) (n = 9,813)
Duplicates (n = 15,142)
Promoter methylation levels in olfatory bulb
pHolm-adj. = 2.98e-03
pHolm-adj. = 2.82e-33
pHolm-adj. = 6.16e-19
pHolm-adj. = 1.36e-49
pHolm-adj. = 2.88e-19
μ̂median = 0.74
μ̂median = 0.72
μ̂median = 0.76
μ̂median = 0.75
Total (n = 19,485)
Singletons (n = 4,181)
Duplicates (non-onhologs) (n = 9,823)
Duplicates (n = 15,152)
Promoter methylation levels in pancreas
pHolm-adj. = 1.73e-05
pHolm-adj. = 7.02e-06
pHolm-adj. = 5.96e-04
μ̂median = 0.66
μ̂median = 0.65
μ̂median = 0.66
μ̂median = 0.66
Total (n = 19,454)
Singletons (n = 4,169)
Duplicates (non-onhologs) (n = 9,804)
Duplicates (n = 15,133)
Promoter methylation levels in skin
pHolm-adj. = 0.04
pHolm-adj. = 1.18e-22
pHolm-adj. = 1.67e-09
pHolm-adj. = 5.55e-29
pHolm-adj. = 2.59e-14
μ̂median = 0.69
μ̂median = 0.67
μ̂median = 0.71
μ̂median = 0.69
Total (n = 19,464)
Singletons (n = 4,167)
Duplicates (non-onhologs) (n = 9,816)
Duplicates (n = 15,145)
Promoter methylation levels in spleen
pHolm-adj. = 8.92e-04
pHolm-adj. = 1.05e-09
pHolm-adj. = 5.07e-05
pHolm-adj. = 3.91e-13
pHolm-adj. = 4.09e-06
μ̂median = 0.76
μ̂median = 0.75
μ̂median = 0.77
μ̂median = 0.76
Total (n = 19,470)
Singletons (n = 4,173)
Duplicates (non-onhologs) (n = 9,817)
Duplicates (n = 15,145)
Promoter methylation levels in stomach
pHolm-adj. = 3.18e-05
pHolm-adj. = 3.32e-15
pHolm-adj. = 2.98e-07
pHolm-adj. = 3.48e-20
pHolm-adj. = 2.12e-09
μ̂median = 0.70
μ̂median = 0.68
μ̂median = 0.71
μ̂median = 0.70
Total (n = 19,425)
Singletons (n = 4,160)
Duplicates (non-onhologs) (n = 9,785)
Duplicates (n = 15,114)
Promoter methylation levels in thymus
pHolm-adj. = 7.76e-03
pHolm-adj. = 1.16e-20
pHolm-adj. = 3.02e-15
pHolm-adj. = 1.18e-34
pHolm-adj. = 2.38e-11
μ̂median = 0.79
μ̂median = 0.77
μ̂median = 0.81
μ̂median = 0.80
Total (n = 19,458)
Singletons (n = 4,173)
Duplicates (non-onhologs) (n = 9,806)
Duplicates (n = 15,135)
Promoter methylation levels in uterus
pHolm-adj. = 2.21e-05
pHolm-adj. = 3.32e-16
pHolm-adj. = 1.84e-07
pHolm-adj. = 2.80e-21
pHolm-adj. = 4.15e-10
μ̂median = 0.71
μ̂median = 0.69
μ̂median = 0.72
μ̂median = 0.71
Total (n = 19,442)
Singletons (n = 4,171)
Duplicates (non-onhologs) (n = 9,791)
Duplicates (n = 15,120)

2. Comparison of gene body methylation levels among the different kinds of orthologs

2.1. Human genes

Below are the representations of levels of gene body methylation in the 10 human tissues analyzed among the different kinds of human-mouse orthologs. In each plot, each dot represents a gene. The horizontal lines indicate significant differences based on two-sided Dunn's pairwise tests with Holm correction for multiple comparisons. Comparisons were deemed significant if corrected p-values were lower than 0.05.

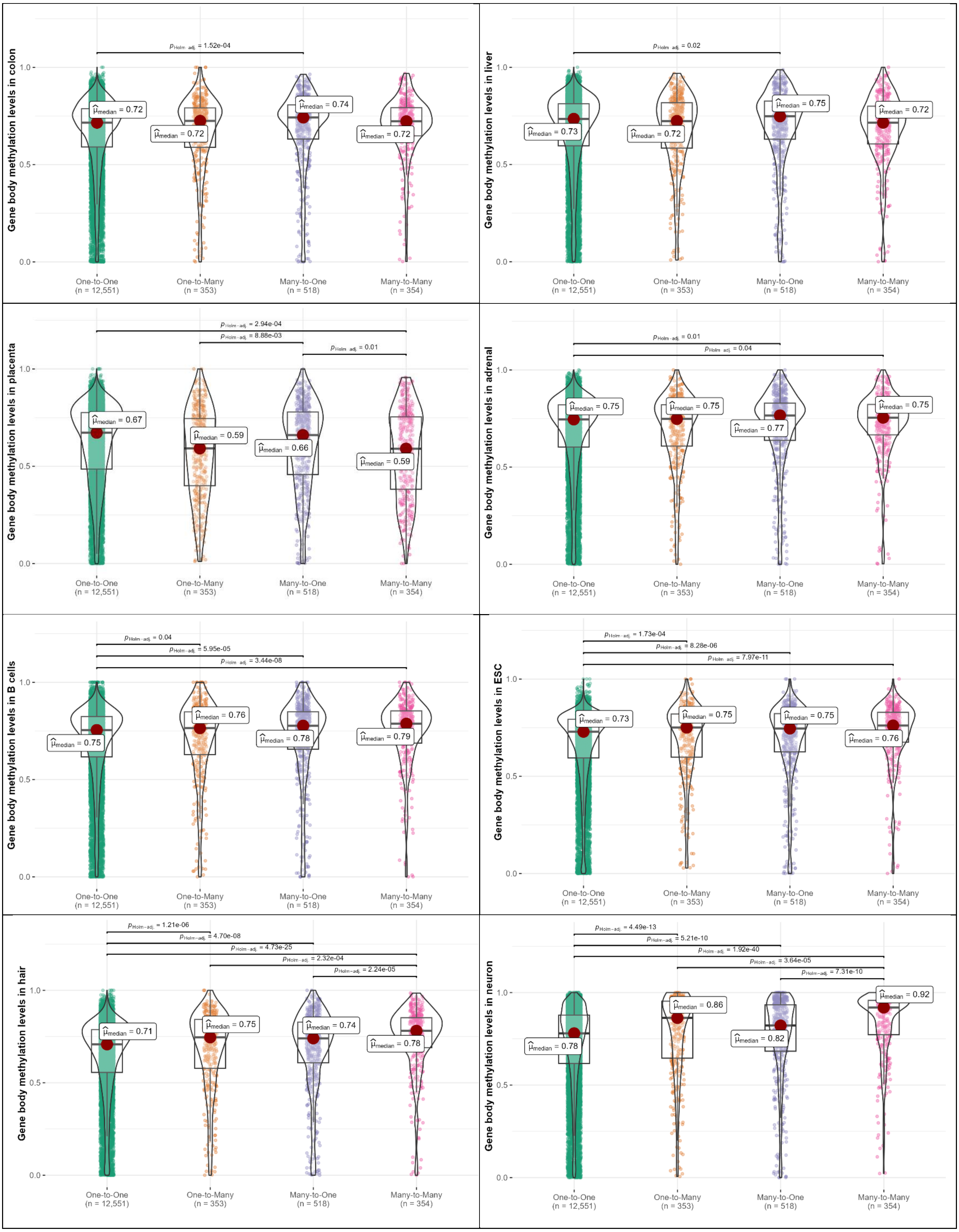

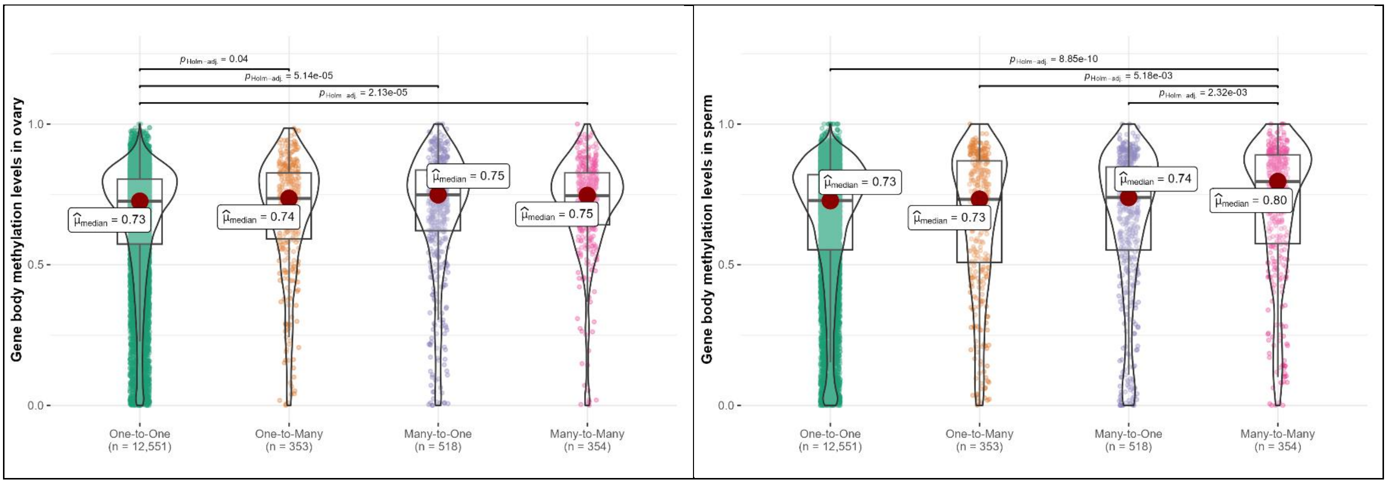

2.2. Mouse genes

Below are the representations of levels of gene body methylation in the 16 mouse tissues analyzed among the different kinds of mouse-human orthologs. In each plot, each dot represents a gene. The horizontal lines indicate significant differences based on two-sided Dunn's pairwise tests with Holm correction for multiple comparisons. Comparisons were deemed significant if corrected p-values were lower than 0.05.

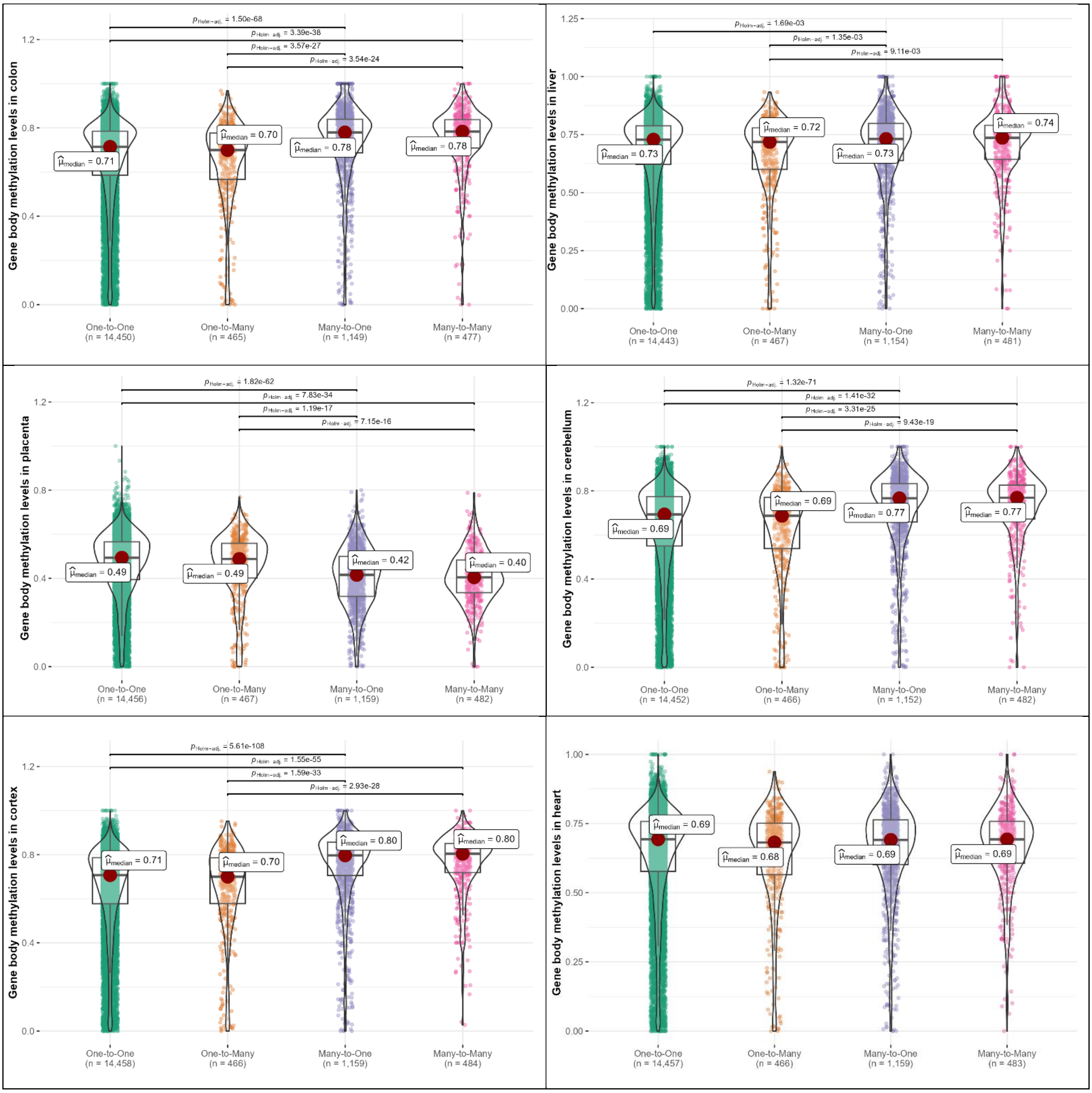

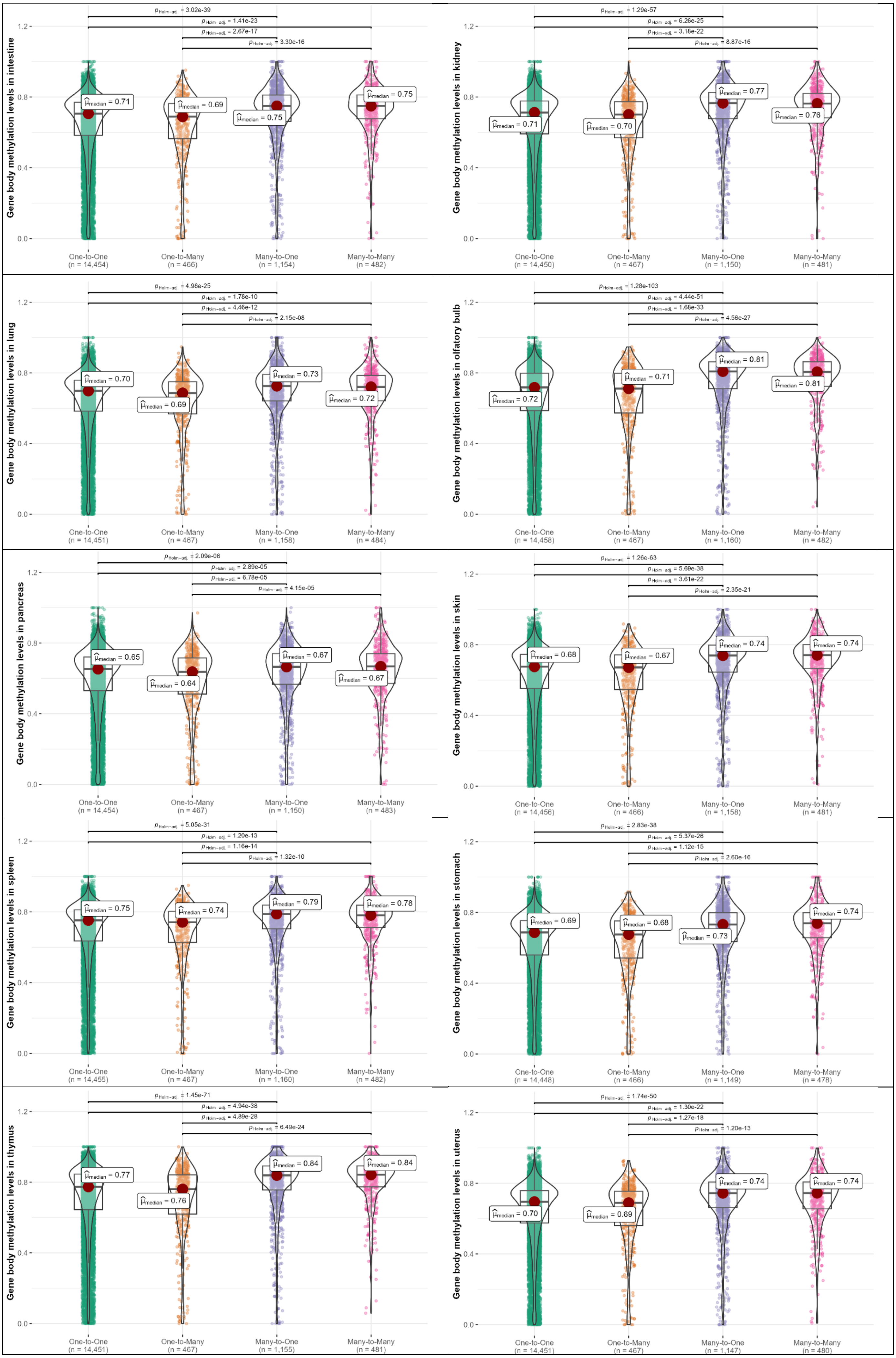

pHolm-adj. = 3.02e-39
pHolm-adj. = 1.41e-23
pHolm-adj. = 2.67e-17
pHolm-adj. = 3.30e-16
Gene body methylation levels in intestine
μ̂median = 0.71
μ̂median = 0.69
μ̂median = 0.75
μ̂median = 0.75
One-to-One (n = 14,454)
One-to-Many (n = 466)
Many-to-One (n = 1,154)
Many-to-Many (n = 482)
pHolm-adj. = 1.29e-57
pHolm-adj. = 6.26e-25
pHolm-adj. = 3.18e-22
pHolm-adj. = 8.87e-16
Gene body methylation levels in kidney
μ̂median = 0.71
μ̂median = 0.70
μ̂median = 0.77
μ̂median = 0.76
One-to-One (n = 14,450)
One-to-Many (n = 467)
Many-to-One (n = 1,150)
Many-to-Many (n = 481)
pHolm-adj. = 4.98e-25
pHolm-adj. = 1.78e-10
pHolm-adj. = 4.46e-12
pHolm-adj. = 2.15e-08
Gene body methylation levels in lung
μ̂median = 0.70
μ̂median = 0.69
μ̂median = 0.73
μ̂median = 0.72
One-to-One (n = 14,451)
One-to-Many (n = 467)
Many-to-One (n = 1,158)
Many-to-Many (n = 484)
pHolm-adj. = 1.28e-103
pHolm-adj. = 4.44e-51
pHolm-adj. = 1.68e-33
pHolm-adj. = 4.56e-27
Gene body methylation levels in olfatory bulb
μ̂median = 0.72
μ̂median = 0.71
μ̂median = 0.81
μ̂median = 0.81
One-to-One (n = 14,458)
One-to-Many (n = 467)
Many-to-One (n = 1,160)
Many-to-Many (n = 482)
pHolm-adj. = 2.09e-06
pHolm-adj. = 2.89e-05
pHolm-adj. = 6.78e-05
pHolm-adj. = 4.15e-05
Gene body methylation levels in pancreas
μ̂median = 0.65
μ̂median = 0.64
μ̂median = 0.67
μ̂median = 0.67
One-to-One (n = 14,454)
One-to-Many (n = 467)
Many-to-One (n = 1,150)
Many-to-Many (n = 483)
pHolm-adj. = 1.26e-63
pHolm-adj. = 5.69e-38
pHolm-adj. = 3.61e-22
pHolm-adj. = 2.35e-21
Gene body methylation levels in skin
μ̂median = 0.68
μ̂median = 0.67
μ̂median = 0.74
μ̂median = 0.74
One-to-One (n = 14,456)
One-to-Many (n = 466)
Many-to-One (n = 1,158)
Many-to-Many (n = 481)
pHolm-adj. = 5.05e-31
pHolm-adj. = 1.20e-13
pHolm-adj. = 1.16e-14
pHolm-adj. = 1.32e-10
Gene body methylation levels in spleen
μ̂median = 0.75
μ̂median = 0.74
μ̂median = 0.79
μ̂median = 0.78
One-to-One (n = 14,455)
One-to-Many (n = 467)
Many-to-One (n = 1,160)
Many-to-Many (n = 482)
pHolm-adj. = 2.83e-38
pHolm-adj. = 5.37e-26
pHolm-adj. = 1.12e-15
pHolm-adj. = 2.60e-16
Gene body methylation levels in stomach
μ̂median = 0.69
μ̂median = 0.68
μ̂median = 0.73
μ̂median = 0.74
One-to-One (n = 14,448)
One-to-Many (n = 466)
Many-to-One (n = 1,149)
Many-to-Many (n = 478)
pHolm-adj. = 1.45e-71
pHolm-adj. = 4.94e-38
pHolm-adj. = 4.89e-28
pHolm-adj. = 6.49e-24
Gene body methylation levels in thymus
μ̂median = 0.77
μ̂median = 0.76
μ̂median = 0.84
μ̂median = 0.84
One-to-One (n = 14,451)
One-to-Many (n = 467)
Many-to-One (n = 1,155)
Many-to-Many (n = 481)
pHolm-adj. = 1.74e-50
pHolm-adj. = 1.30e-22
pHolm-adj. = 1.27e-18
pHolm-adj. = 1.20e-13
Gene body methylation levels in uterus
μ̂median = 0.70
μ̂median = 0.69
μ̂median = 0.74
μ̂median = 0.74
One-to-One (n = 14,451)
One-to-Many (n = 467)
Many-to-One (n = 1,147)
Many-to-Many (n = 480)

3. Correlation of gene body methylation levels of recent duplicates with the Gene Order Conservation (GOC) scores

3.1. Human genes

Below are the representations of the gene body methylation levels among many-to-one genes (duplicated in human, but not duplicated in mouse) with different Gene Order Conservation (GOC) scores, in the 10 human tissues analyzed. In each plot, each dot represents a gene. The horizontal lines indicate significant differences based on two-sided Dunn's pairwise tests with Holm correction for multiple comparisons. Comparisons were deemed significant if corrected p-values were lower than 0.05.

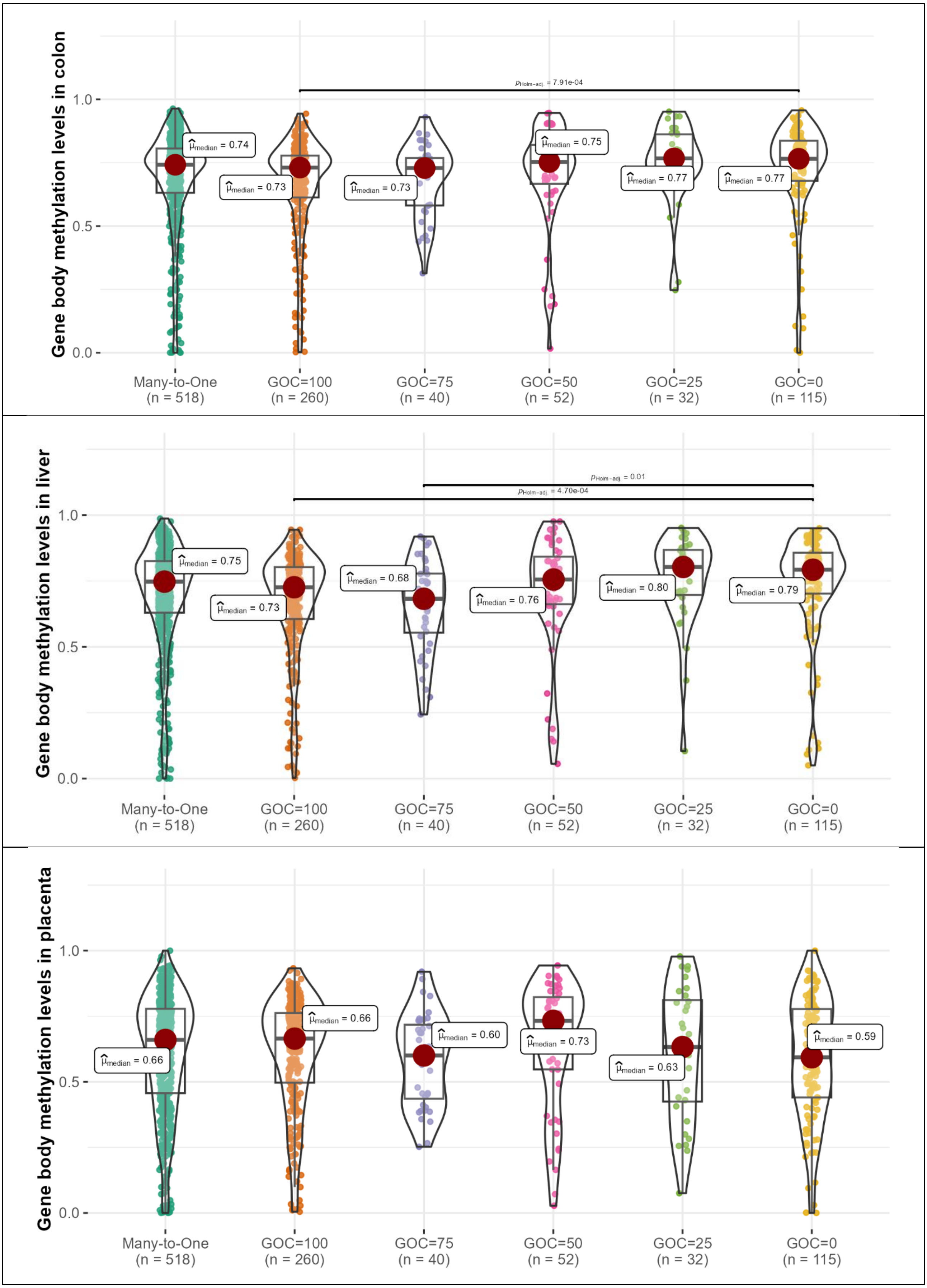

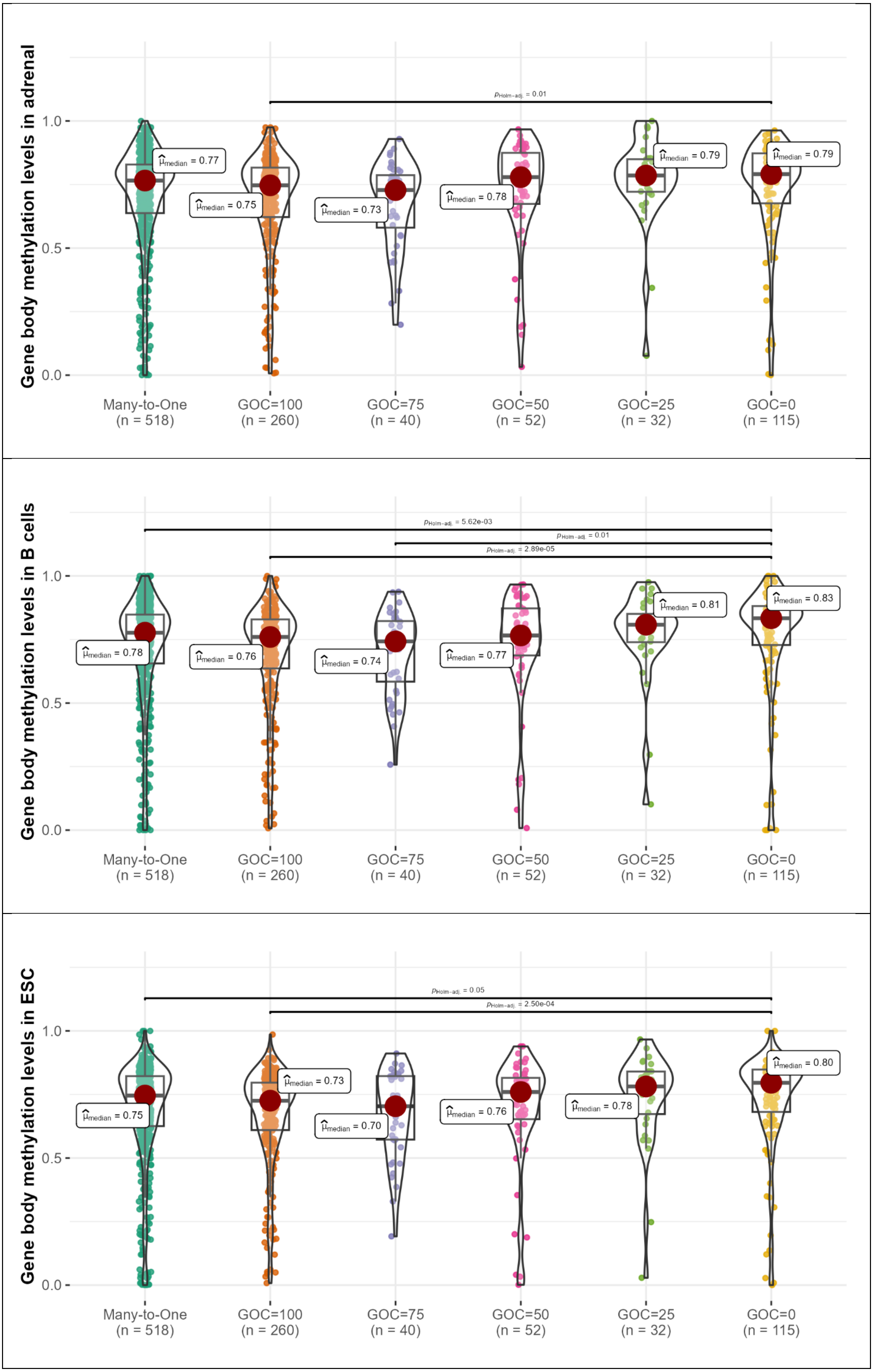
Gene body methylation levels in adrenal
$p_{Holm-adj.}$ = 0.01
$\hat{\mu}_{median}$ = 0.77
$\hat{\mu}_{median}$ = 0.75
$\hat{\mu}_{median}$ = 0.73
$\hat{\mu}_{median}$ = 0.78
$\hat{\mu}_{median}$ = 0.79
$\hat{\mu}_{median}$ = 0.79
1.0
0.5
0.0
Many-to-One (n = 518)
GOC=100 (n = 260)
GOC=75 (n = 40)
GOC=50 (n = 52)
GOC=25 (n = 32)
GOC=0 (n = 115)
Gene body methylation levels in B cells
$p_{Holm-adj.}$ = 5.62e-03
$p_{Holm-adj.}$ = 0.01
$p_{Holm-adj.}$ = 2.89e-05
$\hat{\mu}_{median}$ = 0.78
$\hat{\mu}_{median}$ = 0.76
$\hat{\mu}_{median}$ = 0.74
$\hat{\mu}_{median}$ = 0.77
$\hat{\mu}_{median}$ = 0.81
$\hat{\mu}_{median}$ = 0.83
1.0
0.5
0.0
Many-to-One (n = 518)
GOC=100 (n = 260)
GOC=75 (n = 40)
GOC=50 (n = 52)
GOC=25 (n = 32)
GOC=0 (n = 115)
Gene body methylation levels in ESC
$p_{Holm-adj.}$ = 0.05
$p_{Holm-adj.}$ = 2.50e-04
$\hat{\mu}_{median}$ = 0.75
$\hat{\mu}_{median}$ = 0.73
$\hat{\mu}_{median}$ = 0.70
$\hat{\mu}_{median}$ = 0.76
$\hat{\mu}_{median}$ = 0.78
$\hat{\mu}_{median}$ = 0.80
1.0
0.5
0.0
Many-to-One (n = 518)
GOC=100 (n = 260)
GOC=75 (n = 40)
GOC=50 (n = 52)
GOC=25 (n = 32)
GOC=0 (n = 115)

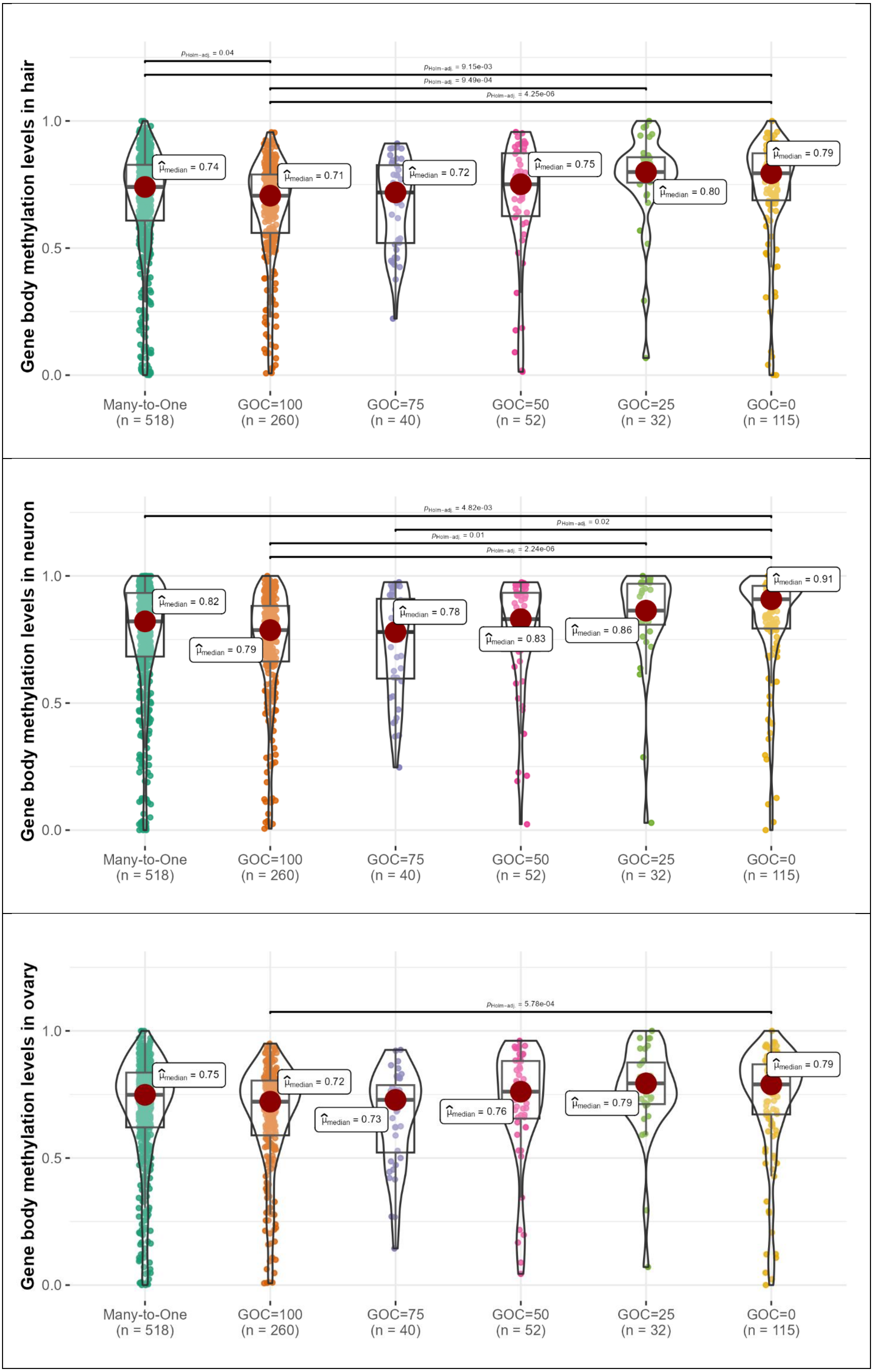

Gene body methylation levels in hair
p Holm-adj. = 0.04
p Holm-adj. = 9.15e-03
p Holm-adj. = 9.49e-04
p Holm-adj. = 4.25e-06
μ̂ median = 0.74
μ̂ median = 0.71
μ̂ median = 0.72
μ̂ median = 0.75
μ̂ median = 0.80
μ̂ median = 0.79
Gene body methylation levels in neuron
p Holm-adj. = 4.82e-03
p Holm-adj. = 0.02
p Holm-adj. = 0.01
p Holm-adj. = 2.24e-06
μ̂ median = 0.82
μ̂ median = 0.79
μ̂ median = 0.78
μ̂ median = 0.83
μ̂ median = 0.86
μ̂ median = 0.91
Gene body methylation levels in ovary
p Holm-adj. = 5.78e-04
μ̂ median = 0.75
μ̂ median = 0.72
μ̂ median = 0.73
μ̂ median = 0.76
μ̂ median = 0.79
μ̂ median = 0.79
0.0
0.5
1.0
Many-to-One (n = 518)
GOC=100 (n = 260)
GOC=75 (n = 40)
GOC=50 (n = 52)
GOC=25 (n = 32)
GOC=0 (n = 115)

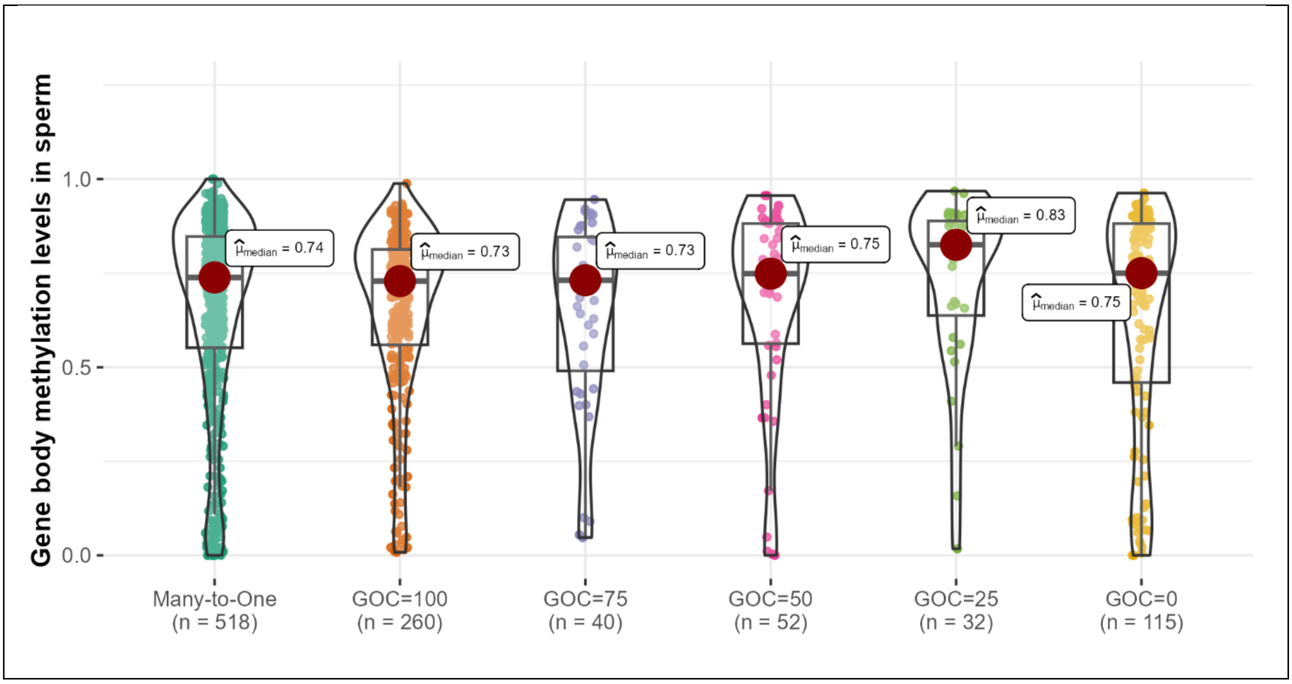

3.2. Mouse genes

Below are the representations of the gene body methylation levels among many-to-one genes (duplicated in mouse, but not duplicated in human) with different Gene Order Conservation (GOC) scores, in the 16 mouse tissues analyzed. In each plot, each dot represents a gene. The horizontal lines indicate significant differences based on two-sided Dunn's pairwise tests with Holm correction for multiple comparisons. Comparisons were deemed significant if corrected p-values were lower than 0.05.

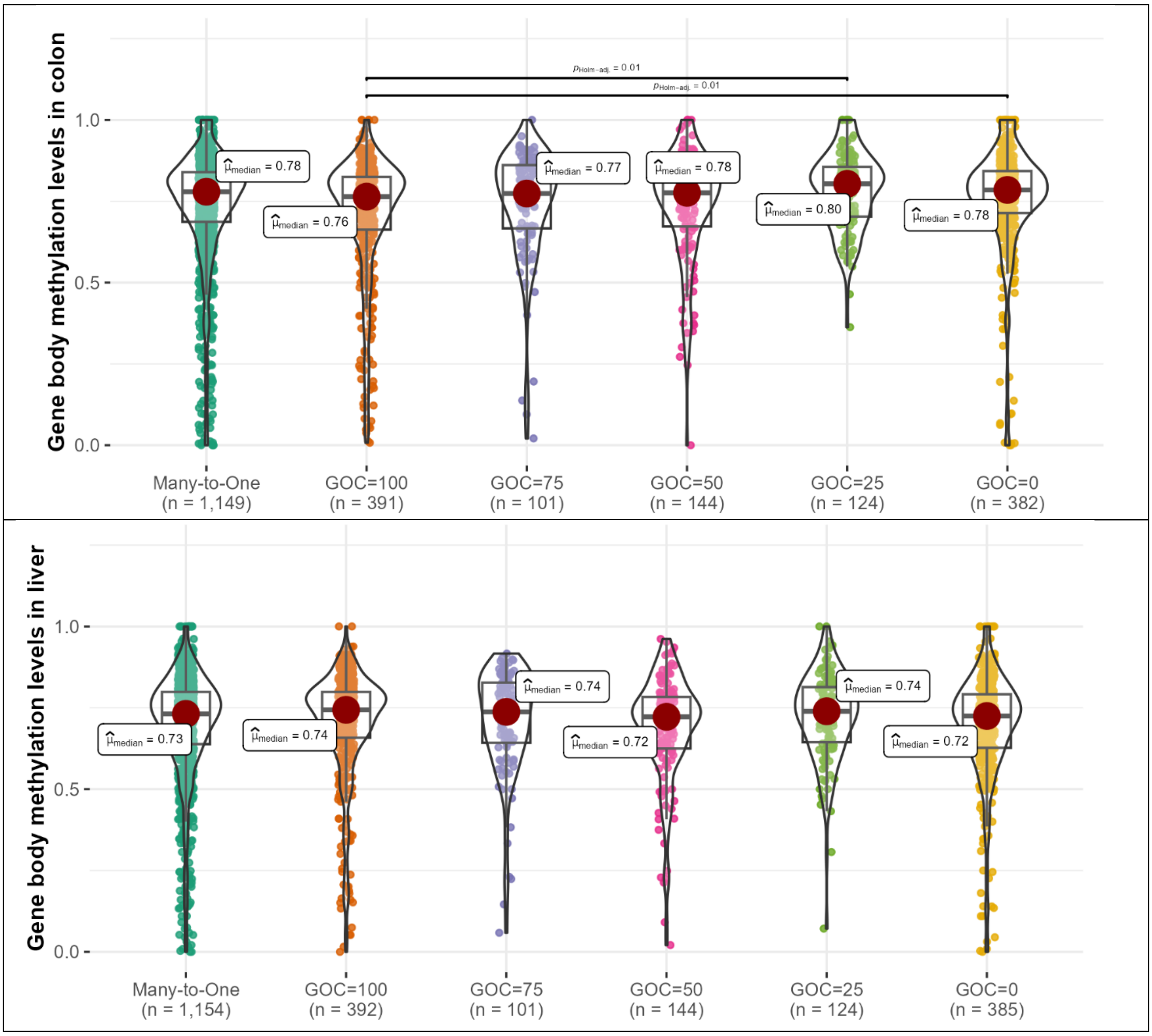

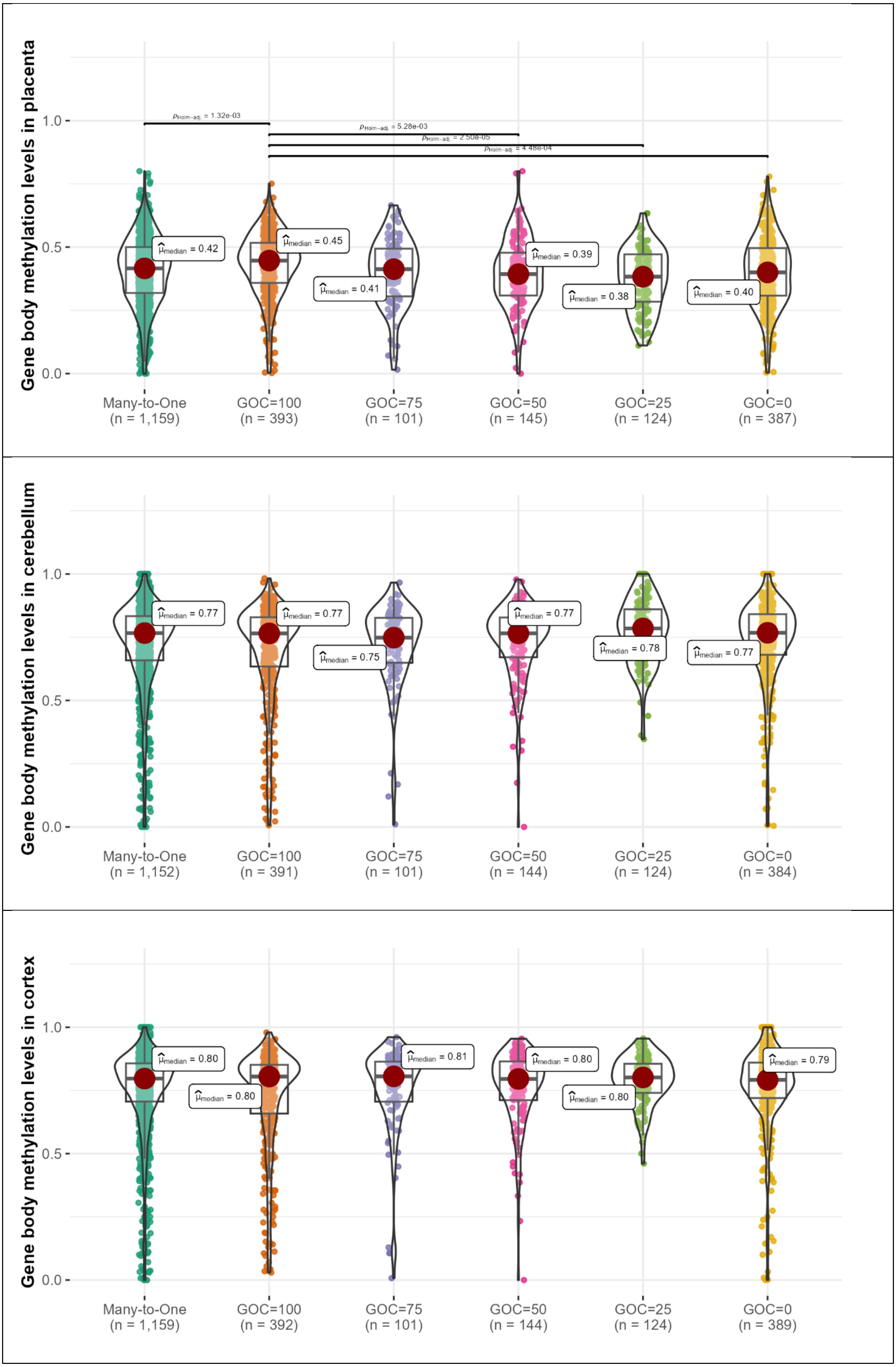
Gene body methylation levels in placenta
1.0
0.5
0.0
pHolm-adj. = 1.32e-03
pHolm-adj. = 5.28e-03
pHolm-adj. = 2.50e-05
pHolm-adj. = 4.48e-04
μ̂median = 0.42
μ̂median = 0.45
μ̂median = 0.41
μ̂median = 0.39
μ̂median = 0.38
μ̂median = 0.40
Many-to-One (n = 1,159)
GOC=100 (n = 393)
GOC=75 (n = 101)
GOC=50 (n = 145)
GOC=25 (n = 124)
GOC=0 (n = 387)
Gene body methylation levels in cerebellum
1.0
0.5
0.0
μ̂median = 0.77
μ̂median = 0.77
μ̂median = 0.75
μ̂median = 0.77
μ̂median = 0.78
μ̂median = 0.77
Many-to-One (n = 1,152)
GOC=100 (n = 391)
GOC=75 (n = 101)
GOC=50 (n = 144)
GOC=25 (n = 124)
GOC=0 (n = 384)
Gene body methylation levels in cortex
1.0
0.5
0.0
μ̂median = 0.80
μ̂median = 0.80
μ̂median = 0.81
μ̂median = 0.80
μ̂median = 0.80
μ̂median = 0.79
Many-to-One (n = 1,159)
GOC=100 (n = 392)
GOC=75 (n = 101)
GOC=50 (n = 144)
GOC=25 (n = 124)
GOC=0 (n = 389)

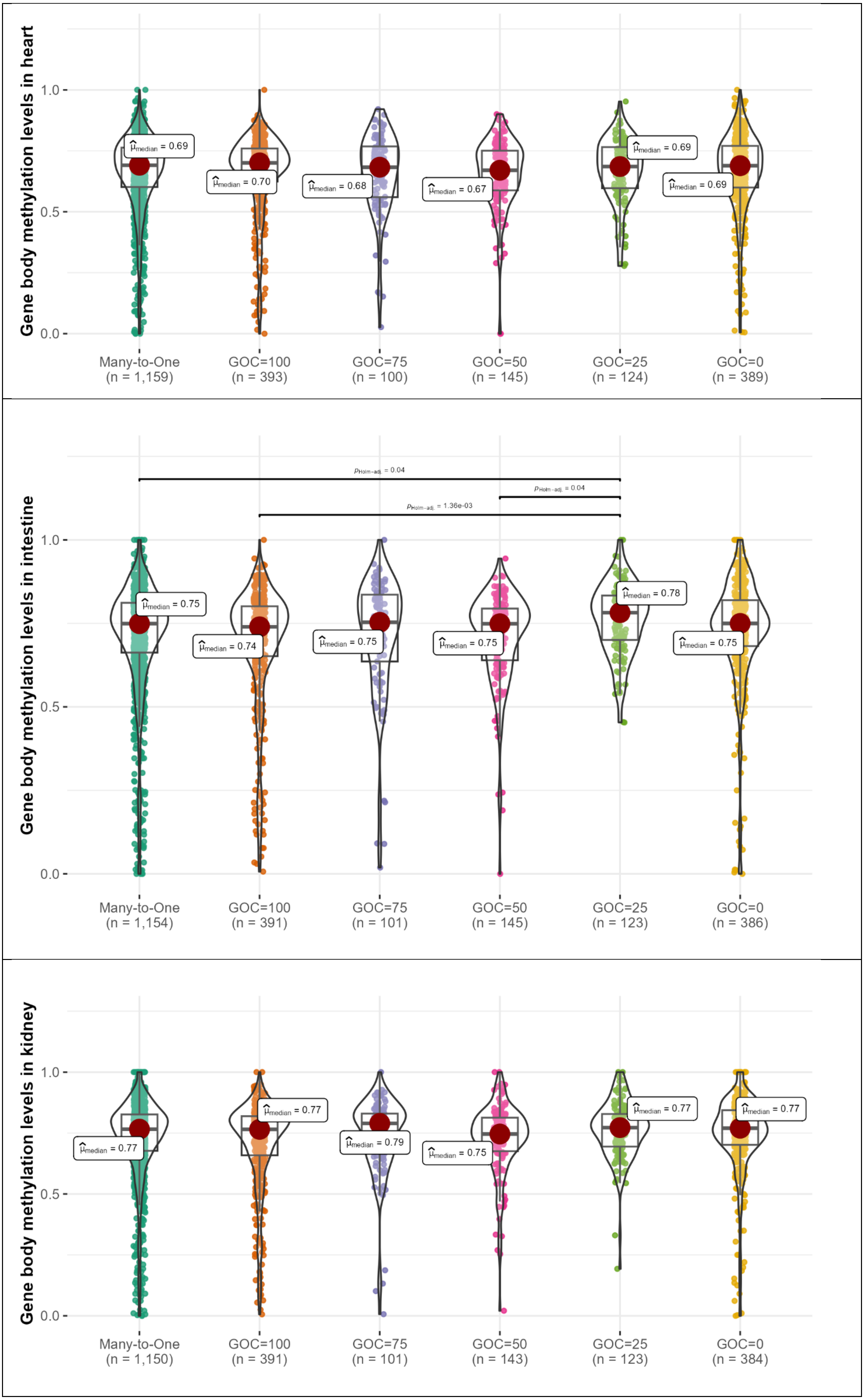

Gene body methylation levels in heart
1.0
0.5
0.0
μ̂median = 0.69
μ̂median = 0.70
μ̂median = 0.68
μ̂median = 0.67
μ̂median = 0.69
μ̂median = 0.69
Many-to-One (n = 1,159)
GOC=100 (n = 393)
GOC=75 (n = 100)
GOC=50 (n = 145)
GOC=25 (n = 124)
GOC=0 (n = 389)
Gene body methylation levels in intestine
pHolm-adj. = 0.04
pHolm-adj. = 0.04
pHolm-adj. = 1.36e-03
1.0
0.5
0.0
μ̂median = 0.75
μ̂median = 0.74
μ̂median = 0.75
μ̂median = 0.75
μ̂median = 0.78
μ̂median = 0.75
Many-to-One (n = 1,154)
GOC=100 (n = 391)
GOC=75 (n = 101)
GOC=50 (n = 145)
GOC=25 (n = 123)
GOC=0 (n = 386)
Gene body methylation levels in kidney
1.0
0.5
0.0
μ̂median = 0.77
μ̂median = 0.77
μ̂median = 0.79
μ̂median = 0.75
μ̂median = 0.77
μ̂median = 0.77
Many-to-One (n = 1,150)
GOC=100 (n = 391)
GOC=75 (n = 101)
GOC=50 (n = 143)
GOC=25 (n = 123)
GOC=0 (n = 384)

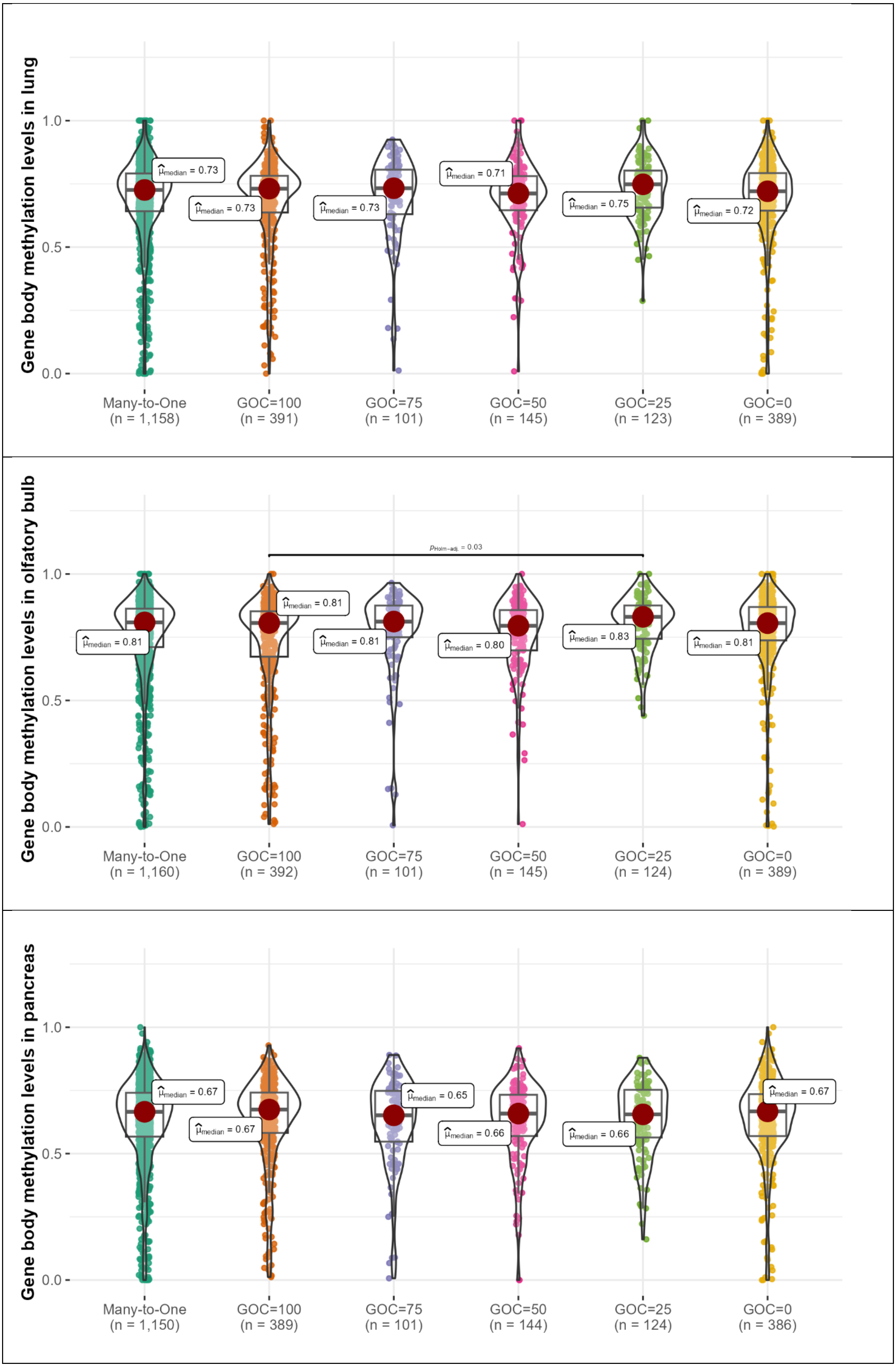
Gene body methylation levels in lung
1.0
0.5
0.0
μ̂median = 0.73
μ̂median = 0.73
μ̂median = 0.73
μ̂median = 0.71
μ̂median = 0.75
μ̂median = 0.72
Many-to-One (n = 1,158)
GOC=100 (n = 391)
GOC=75 (n = 101)
GOC=50 (n = 145)
GOC=25 (n = 123)
GOC=0 (n = 389)
Gene body methylation levels in olfatory bulb
pHolm-adj. = 0.03
1.0
0.5
0.0
μ̂median = 0.81
μ̂median = 0.81
μ̂median = 0.81
μ̂median = 0.80
μ̂median = 0.83
μ̂median = 0.81
Many-to-One (n = 1,160)
GOC=100 (n = 392)
GOC=75 (n = 101)
GOC=50 (n = 145)
GOC=25 (n = 124)
GOC=0 (n = 389)
Gene body methylation levels in pancreas
1.0
0.5
0.0
μ̂median = 0.67
μ̂median = 0.67
μ̂median = 0.65
μ̂median = 0.66
μ̂median = 0.66
μ̂median = 0.67
Many-to-One (n = 1,150)
GOC=100 (n = 389)
GOC=75 (n = 101)
GOC=50 (n = 144)
GOC=25 (n = 124)
GOC=0 (n = 386)

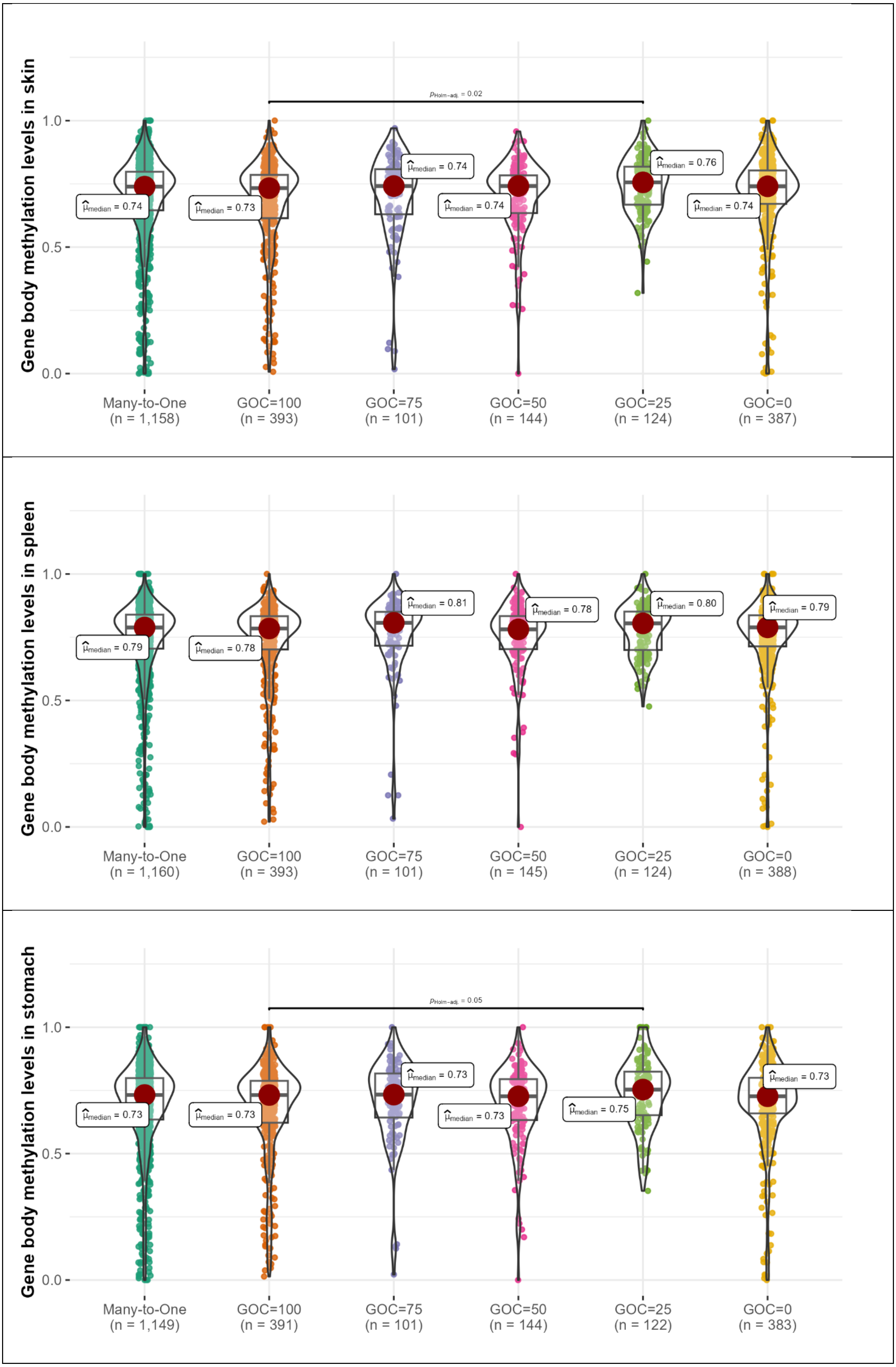
Gene body methylation levels in skin
pHolm-adj. = 0.02
μ̂median = 0.74
μ̂median = 0.73
μ̂median = 0.74
μ̂median = 0.74
μ̂median = 0.76
μ̂median = 0.74
1.0
0.5
0.0
Many-to-One (n = 1,158)
GOC=100 (n = 393)
GOC=75 (n = 101)
GOC=50 (n = 144)
GOC=25 (n = 124)
GOC=0 (n = 387)
Gene body methylation levels in spleen
μ̂median = 0.79
μ̂median = 0.78
μ̂median = 0.81
μ̂median = 0.78
μ̂median = 0.80
μ̂median = 0.79
1.0
0.5
0.0
Many-to-One (n = 1,160)
GOC=100 (n = 393)
GOC=75 (n = 101)
GOC=50 (n = 145)
GOC=25 (n = 124)
GOC=0 (n = 388)
Gene body methylation levels in stomach
pHolm-adj. = 0.05
μ̂median = 0.73
μ̂median = 0.73
μ̂median = 0.73
μ̂median = 0.73
μ̂median = 0.75
μ̂median = 0.73
1.0
0.5
0.0
Many-to-One (n = 1,149)
GOC=100 (n = 391)
GOC=75 (n = 101)
GOC=50 (n = 144)
GOC=25 (n = 122)
GOC=0 (n = 383)

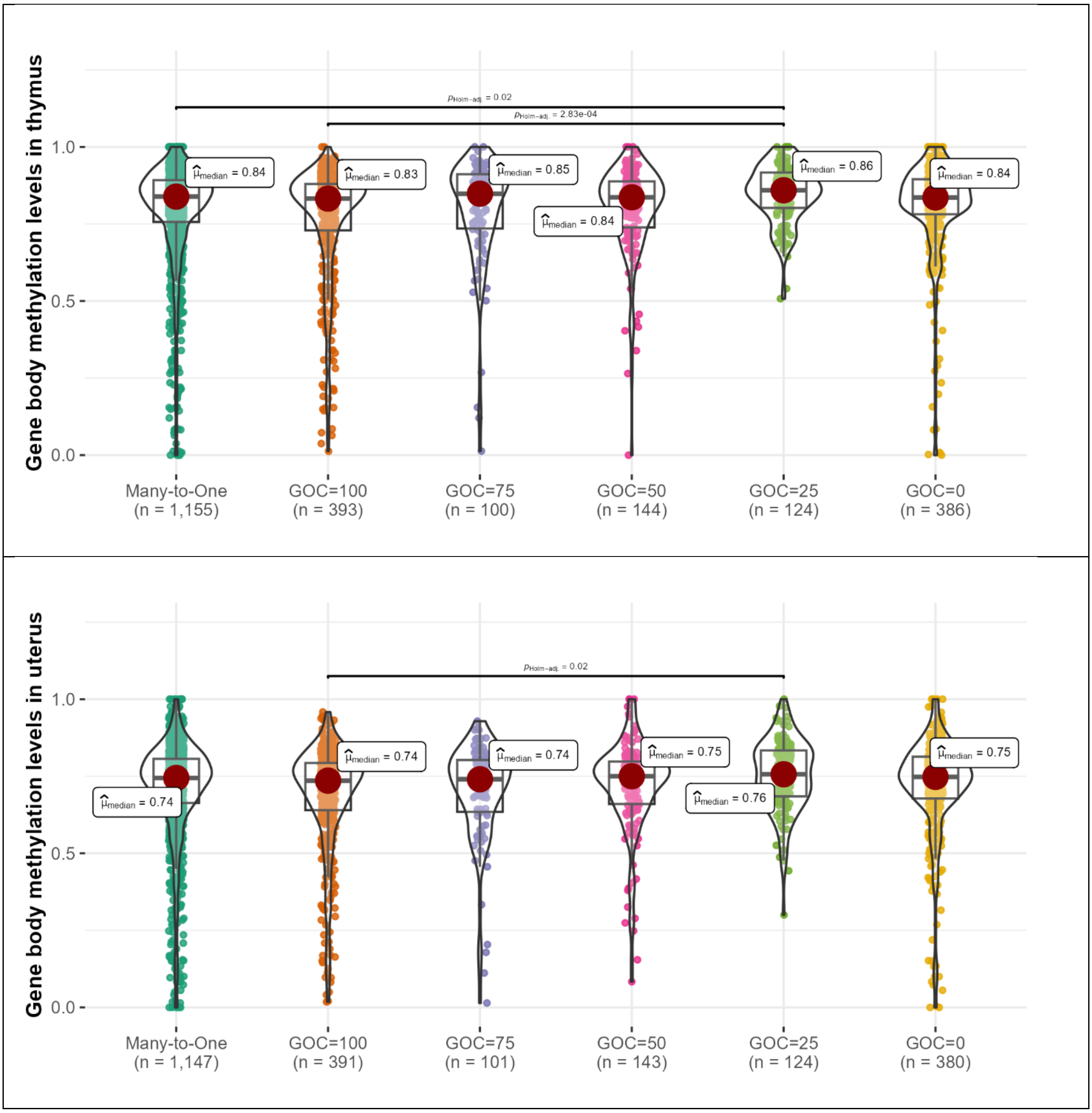
Gene body methylation levels in thymus
p_Holm-adj. = 0.02
p_Holm-adj. = 2.83e-04
μ̂_median = 0.84
μ̂_median = 0.83
μ̂_median = 0.85
μ̂_median = 0.84
μ̂_median = 0.86
μ̂_median = 0.84
1.0
0.5
0.0
Many-to-One (n = 1,155)
GOC=100 (n = 393)
GOC=75 (n = 100)
GOC=50 (n = 144)
GOC=25 (n = 124)
GOC=0 (n = 386)
Gene body methylation levels in uterus
p_Holm-adj. = 0.02
μ̂_median = 0.74
μ̂_median = 0.74
μ̂_median = 0.74
μ̂_median = 0.75
μ̂_median = 0.76
μ̂_median = 0.75
1.0
0.5
0.0
Many-to-One (n = 1,147)
GOC=100 (n = 391)
GOC=75 (n = 101)
GOC=50 (n = 143)
GOC=25 (n = 124)
GOC=0 (n = 380)

4. Gene body methylation in trios duplicated in human but not in mouse

4.1. Comparison of gene body methylation of human daughter copies, human parental copies, and mouse orthologs

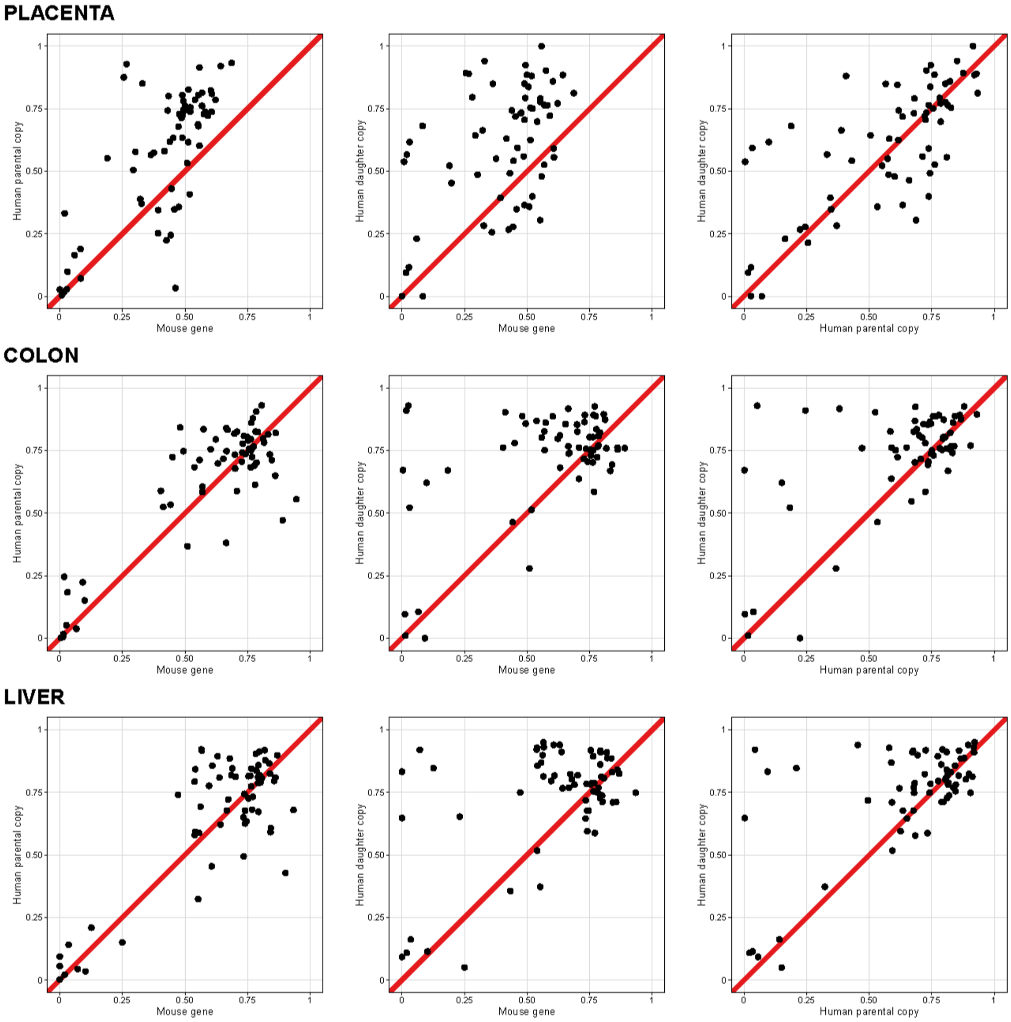

Each dot represents a pair of genes within a trio. For each graph, the red line corresponds to an equal amount of methylation in both genes.

4.2. Comparison of gene body methylation of human daughter copies, human parental copies, and mouse orthologs: violin plots

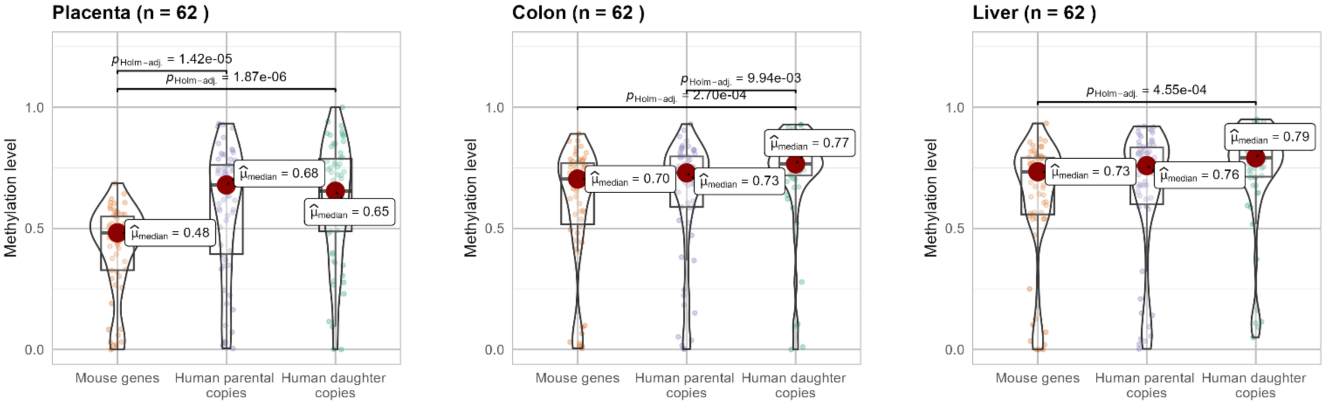

4.3. Comparison of gene body methylation of human daughter copies, human parental copies, and mouse orthologs without retrogenes

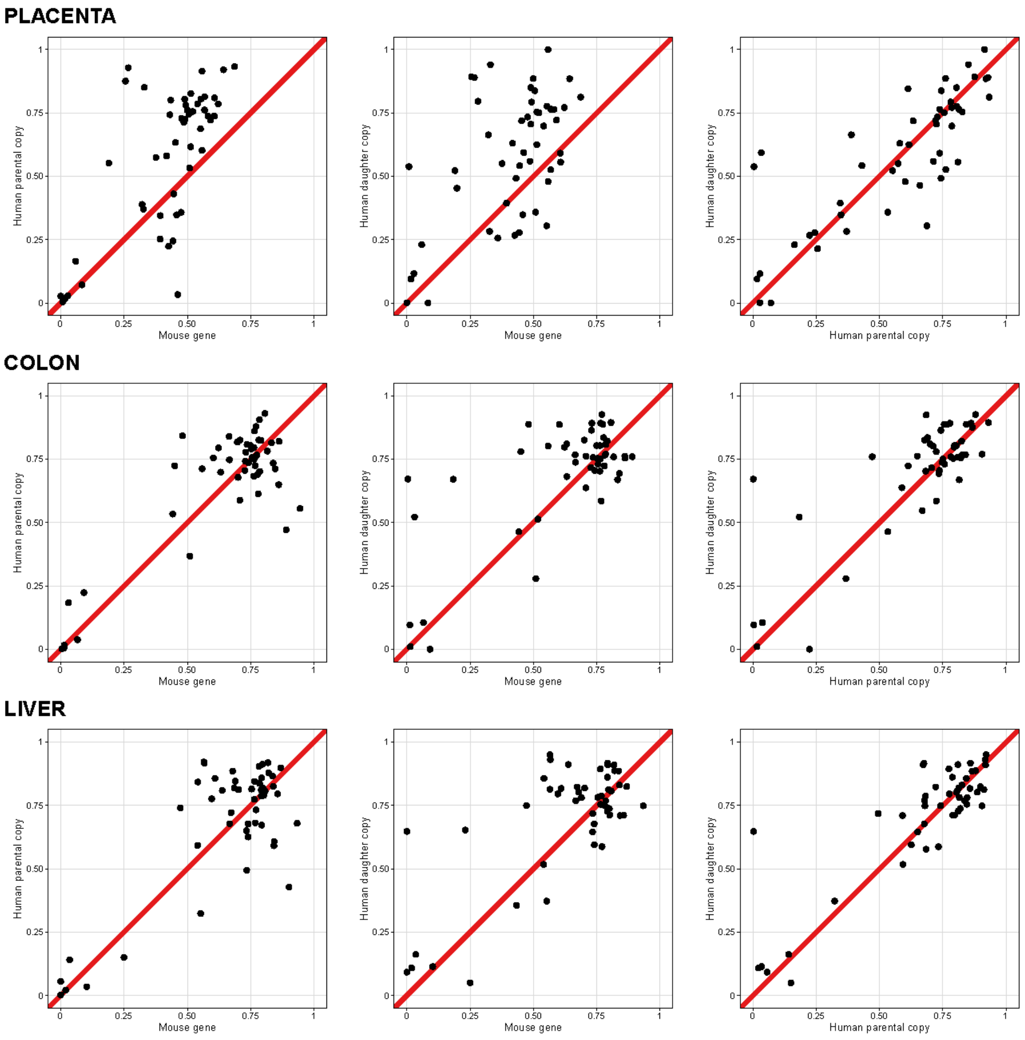

Each dot represents a pair of genes within a trio. For each graph, the red line corresponds to an equal amount of methylation in both genes.

4.4. Comparison of gene body methylation of human daughter copies, human parental copies, and mouse orthologs without retrogenes: violin plots

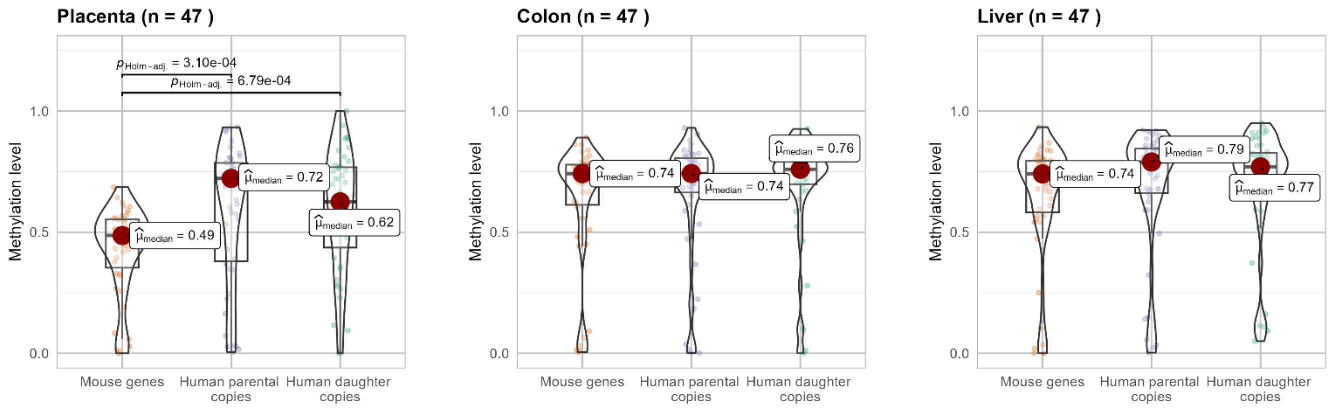

4.5. Comparison of gene body methylation of human daughter vs. human parental copies

Each dot represents a pair of genes within a trio. For each graph, the red line corresponds to an equal amount of methylation in both genes.

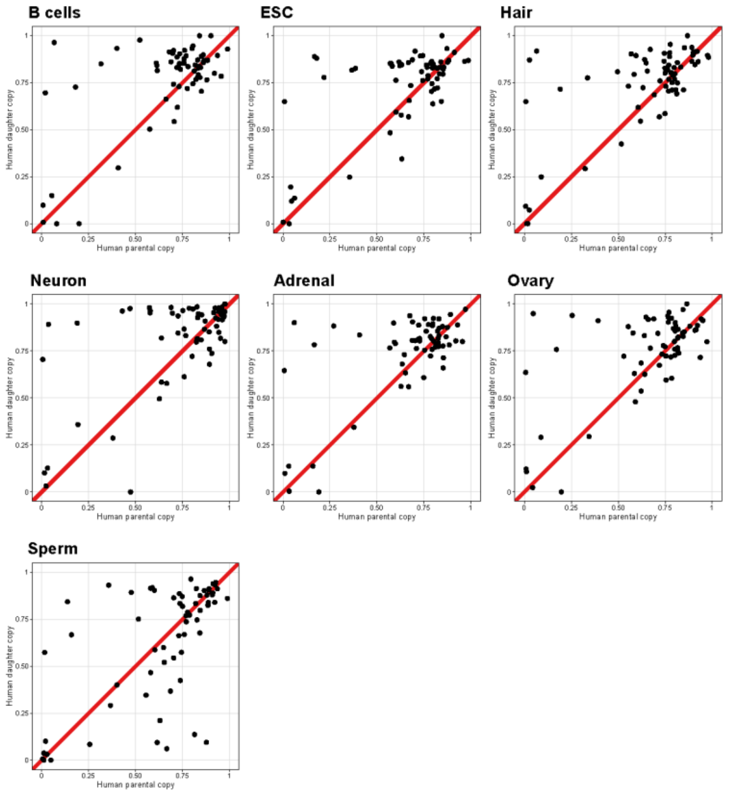

Each dot represents a pair of genes within a trio. For each graph, the red line corresponds to an equal amount of methylation in both genes.

4.6. Comparison of gene body methylation of human daughter vs. human parental copies: violin plots

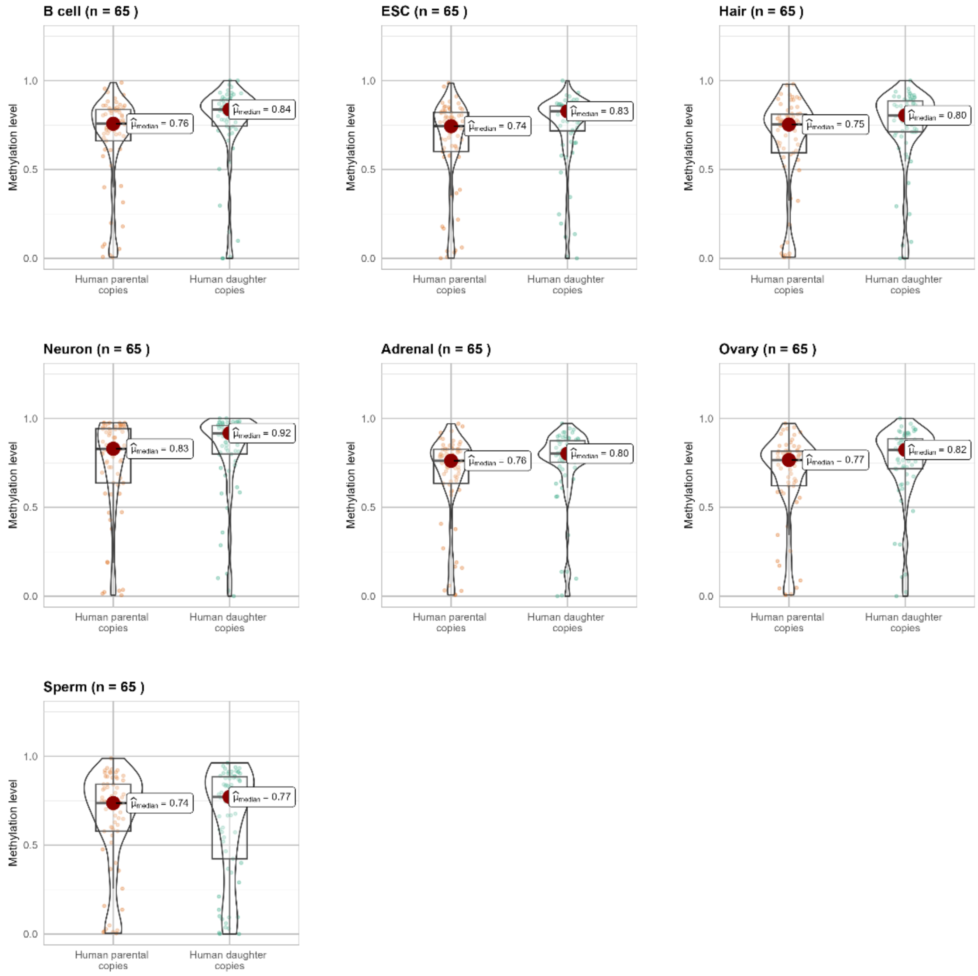

4.7. Comparison of gene body methylation of human daughter vs. human parental copies without retrogenes

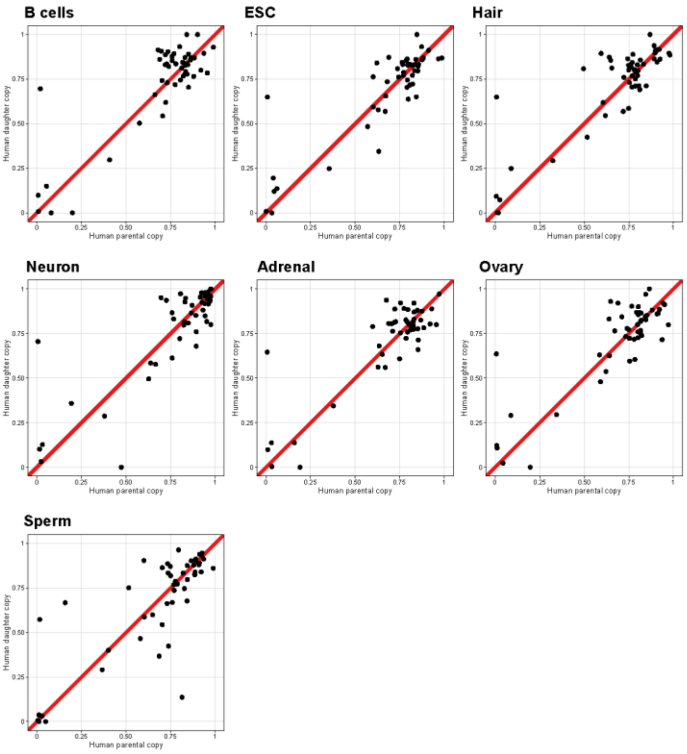

Each dot represents a pair of genes within a trio. For each graph, the red line corresponds to an equal amount of methylation in both genes.

4.8. Comparison of gene body methylation of human daughter vs. human parental copies without retrogenes: violin plots

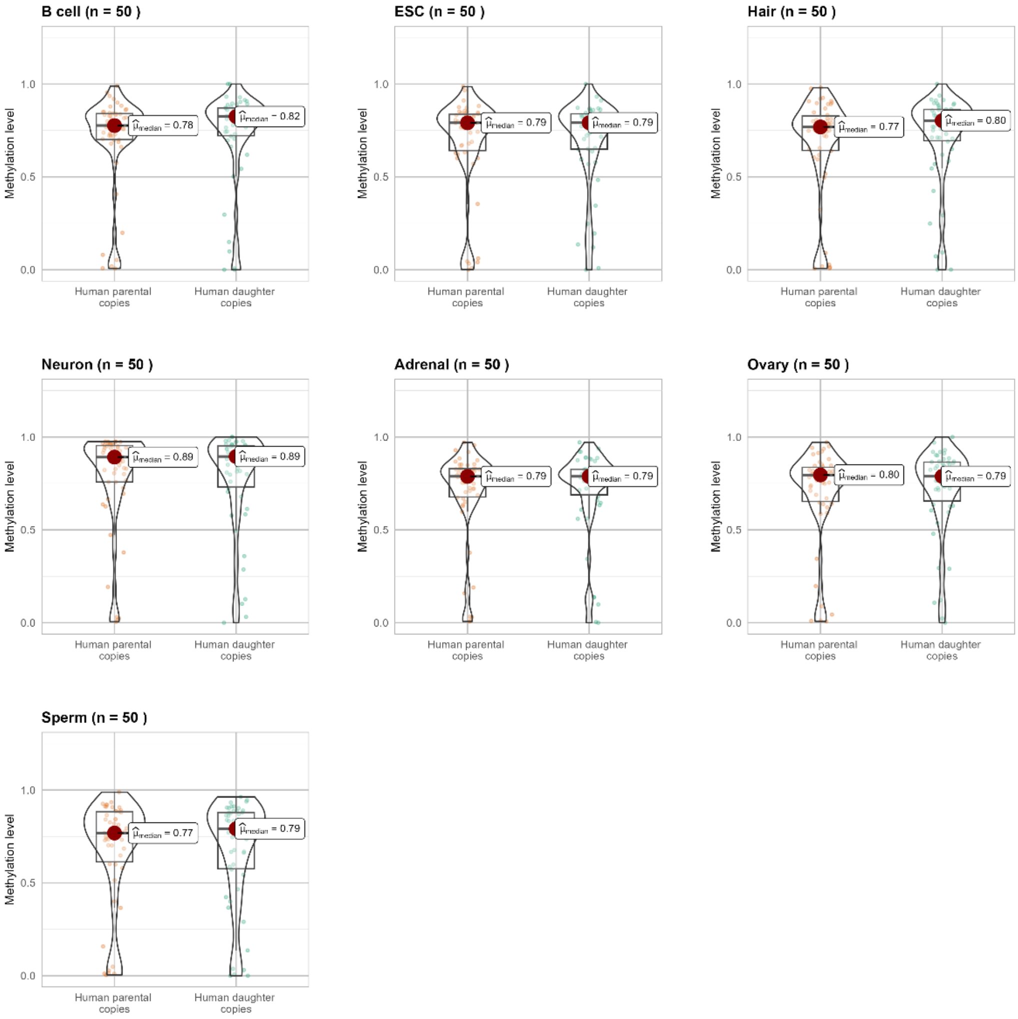

5. Gene body methylation in trios duplicated in mouse but not in human

5.1. Comparison of gene body methylation of mouse daughter copies, mouse parental copies, and human orthologs

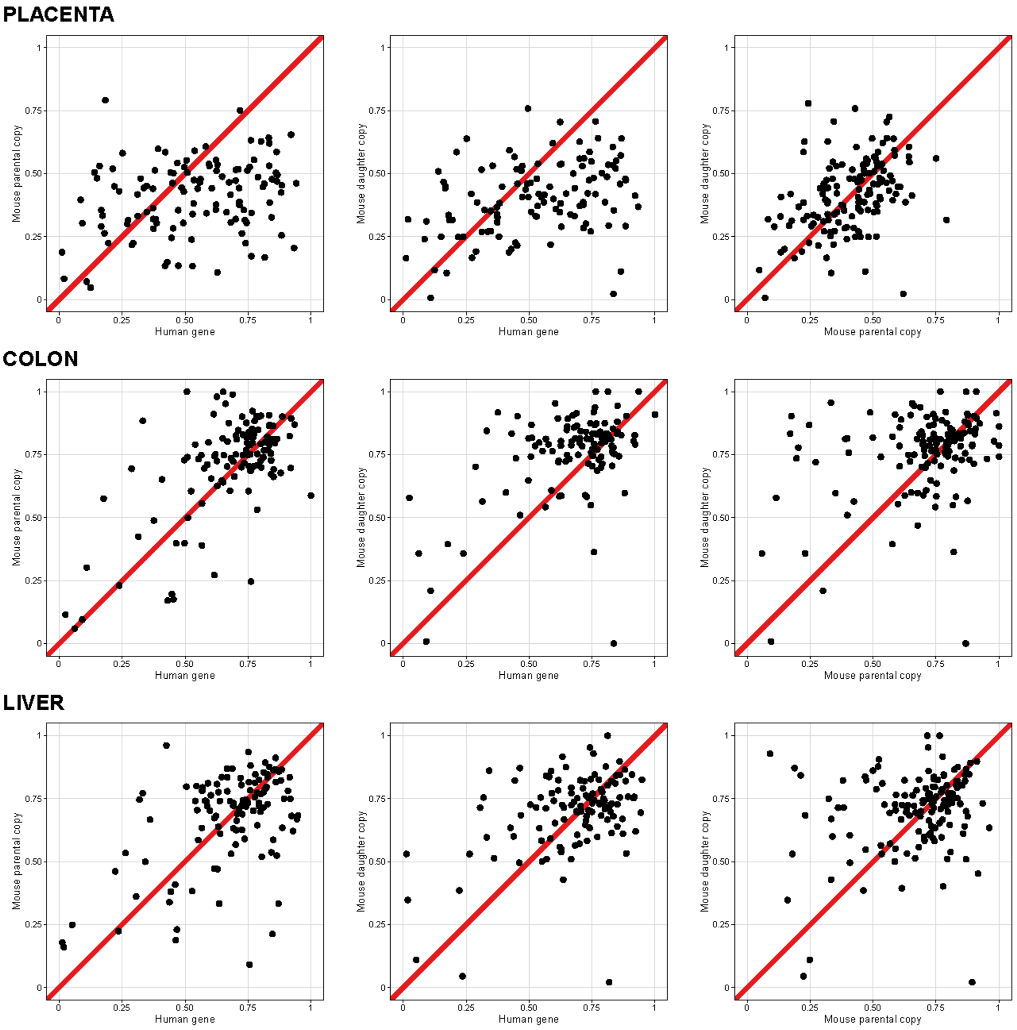

Each dot represents a pair of genes within a trio. For each graph, the red line corresponds to an equal amount of methylation in both genes.

5.2. Comparison of gene body methylation of mouse daughter copies, mouse parental copies, and human orthologs: violin plots

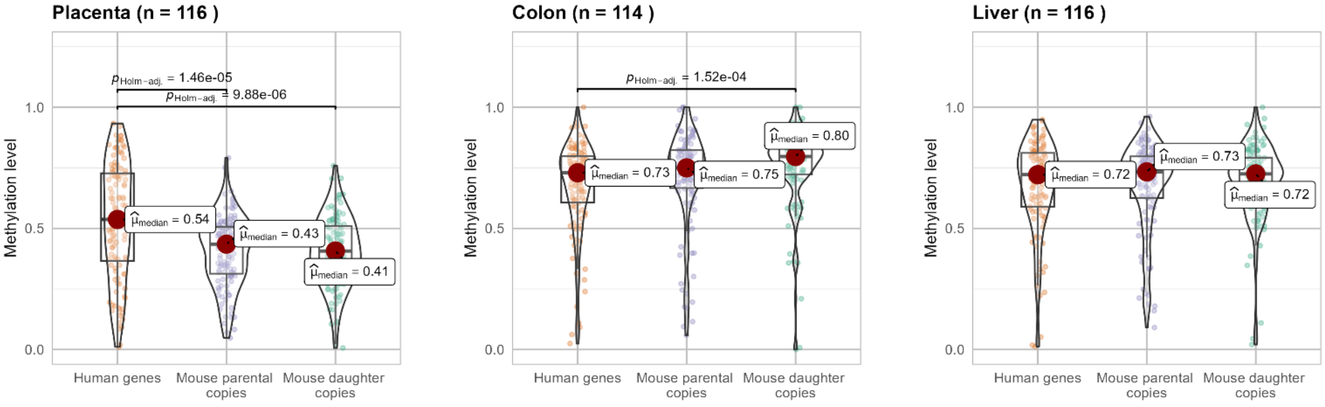

5.3. Comparison of gene body methylation of mouse daughter copies, mouse parental copies, and human orthologs without retrogenes

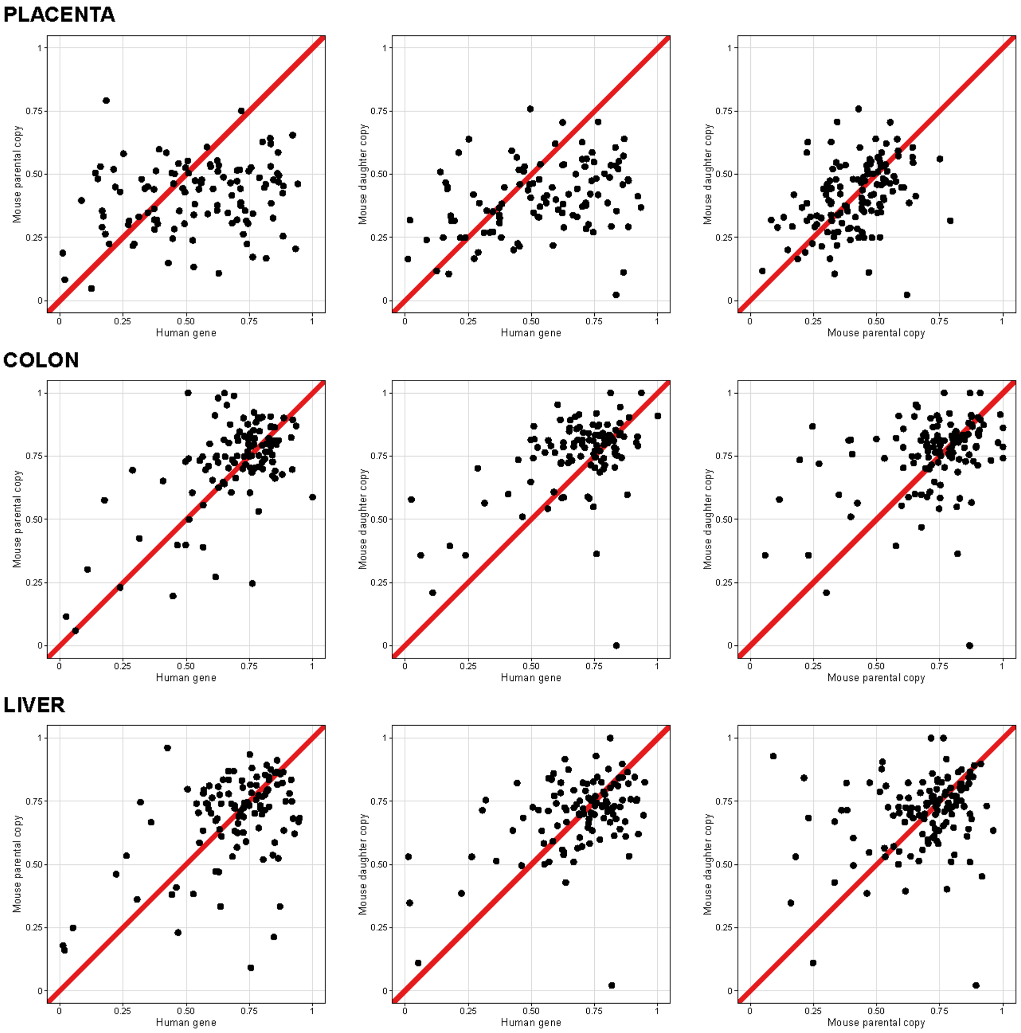

Each dot represents a pair of genes within a trio. For each graph, the red line corresponds to an equal amount of methylation in both genes.

5.4. Comparison of gene body methylation of mouse daughter copies, mouse parental copies, and human orthologs without retrogenes: violin plots

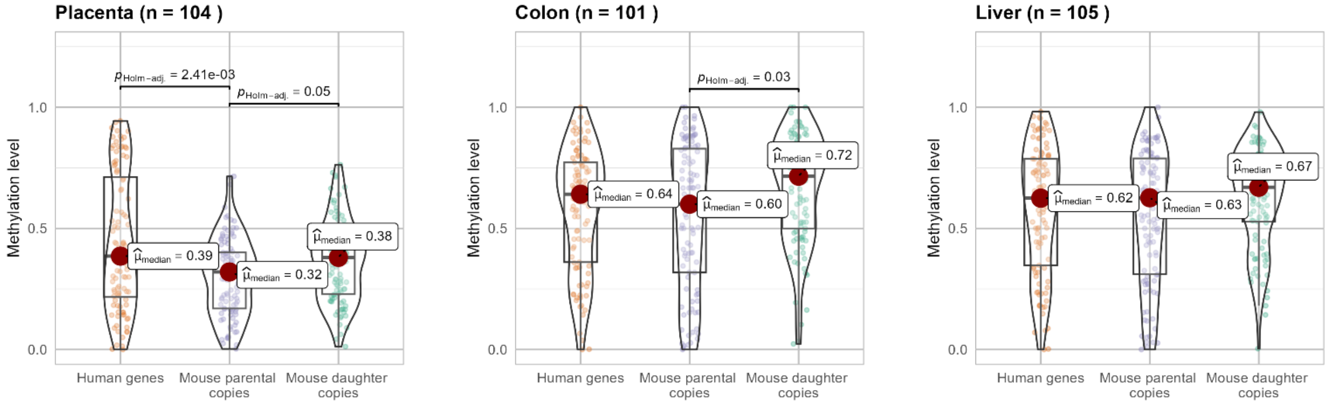

5.5. Comparison of gene body methylation of mouse daughter vs. mouse parental copies

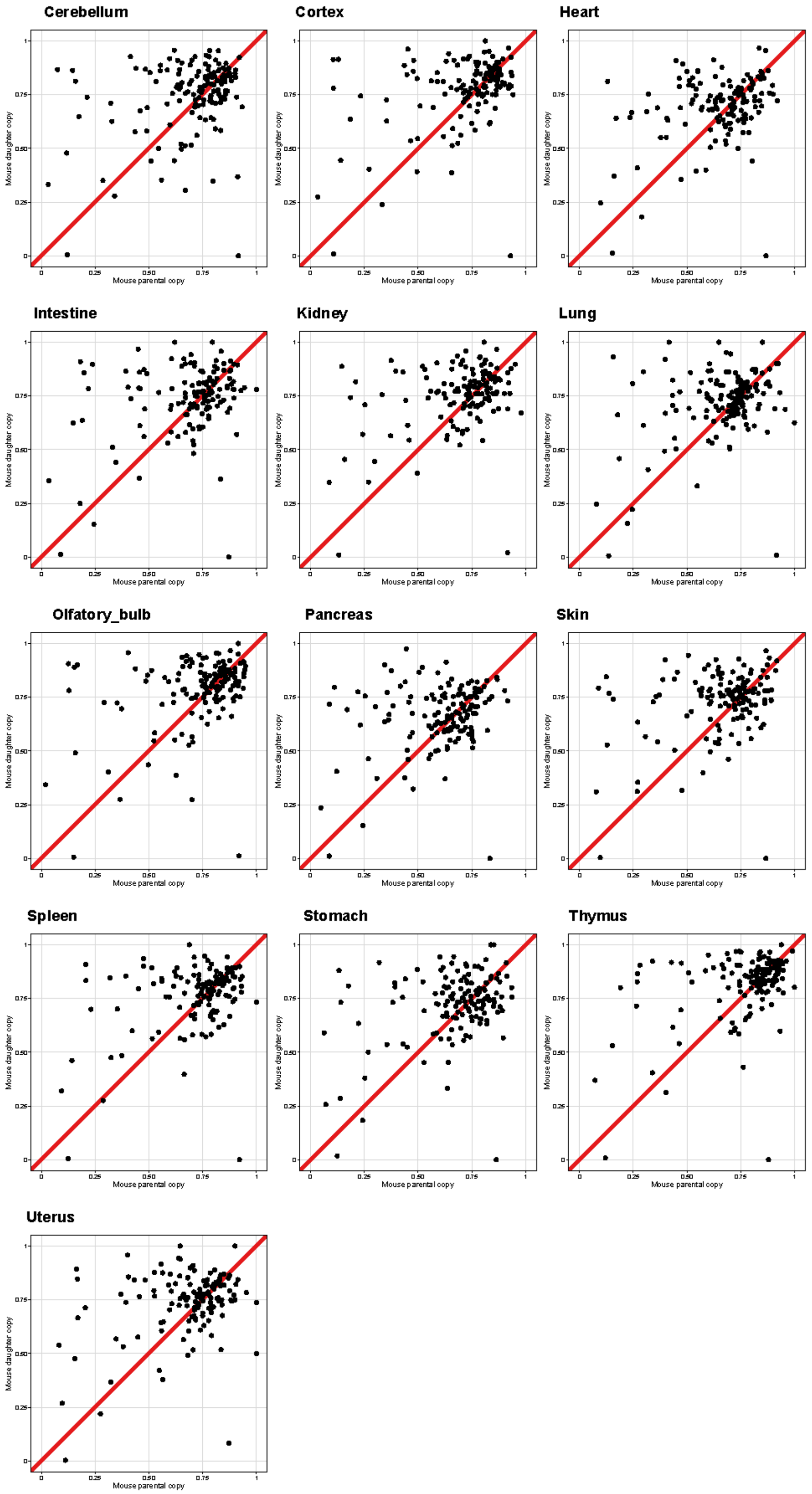

Each dot represents a pair of genes within a trio. For each graph, the red line corresponds to an equal amount of methylation in both genes.

5.6. Comparison of gene body methylation of mouse daughter vs. mouse parental copies: violin plots

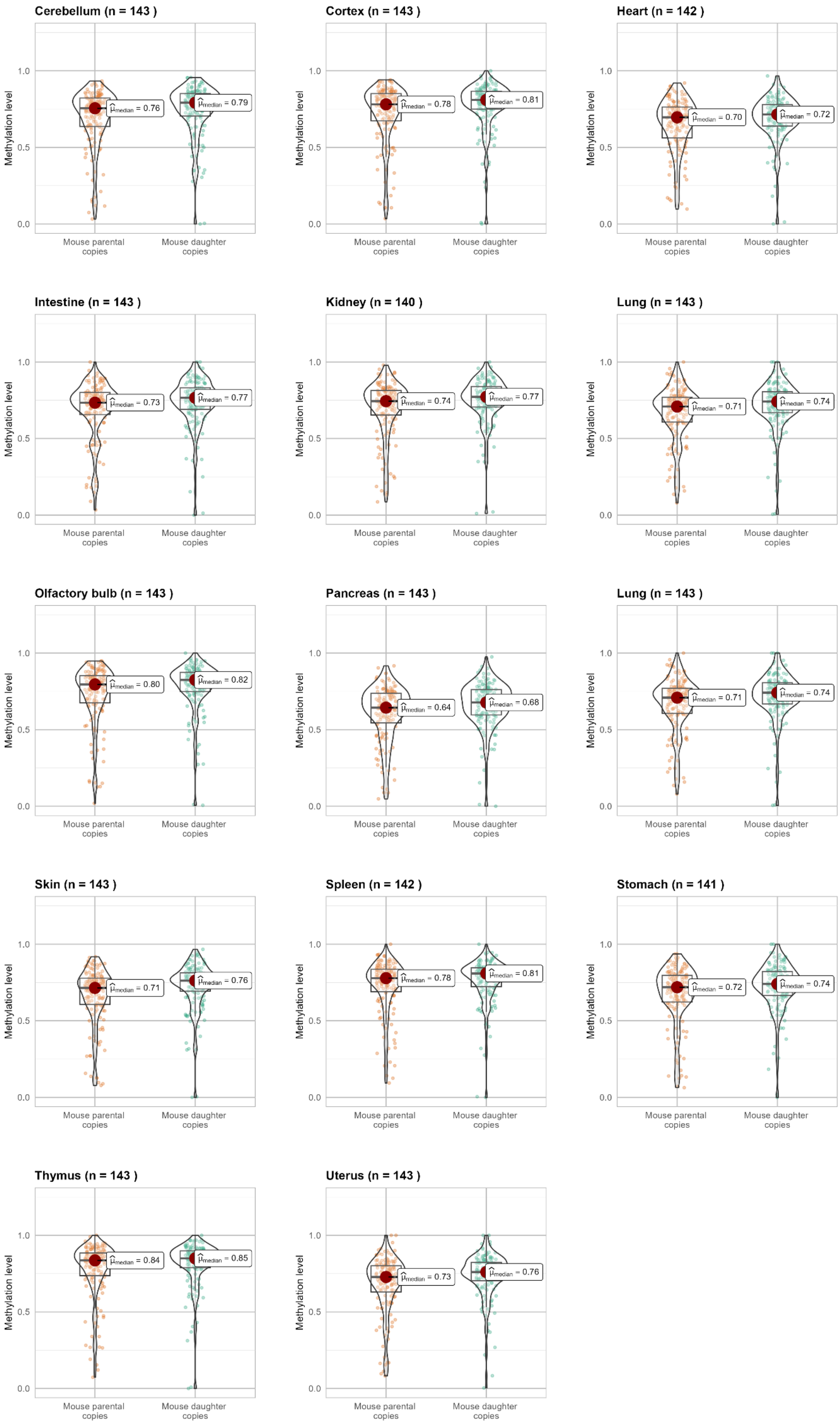

5.7. Comparison of gene body methylation of mouse daughter vs. mouse parental copies without retrogenes

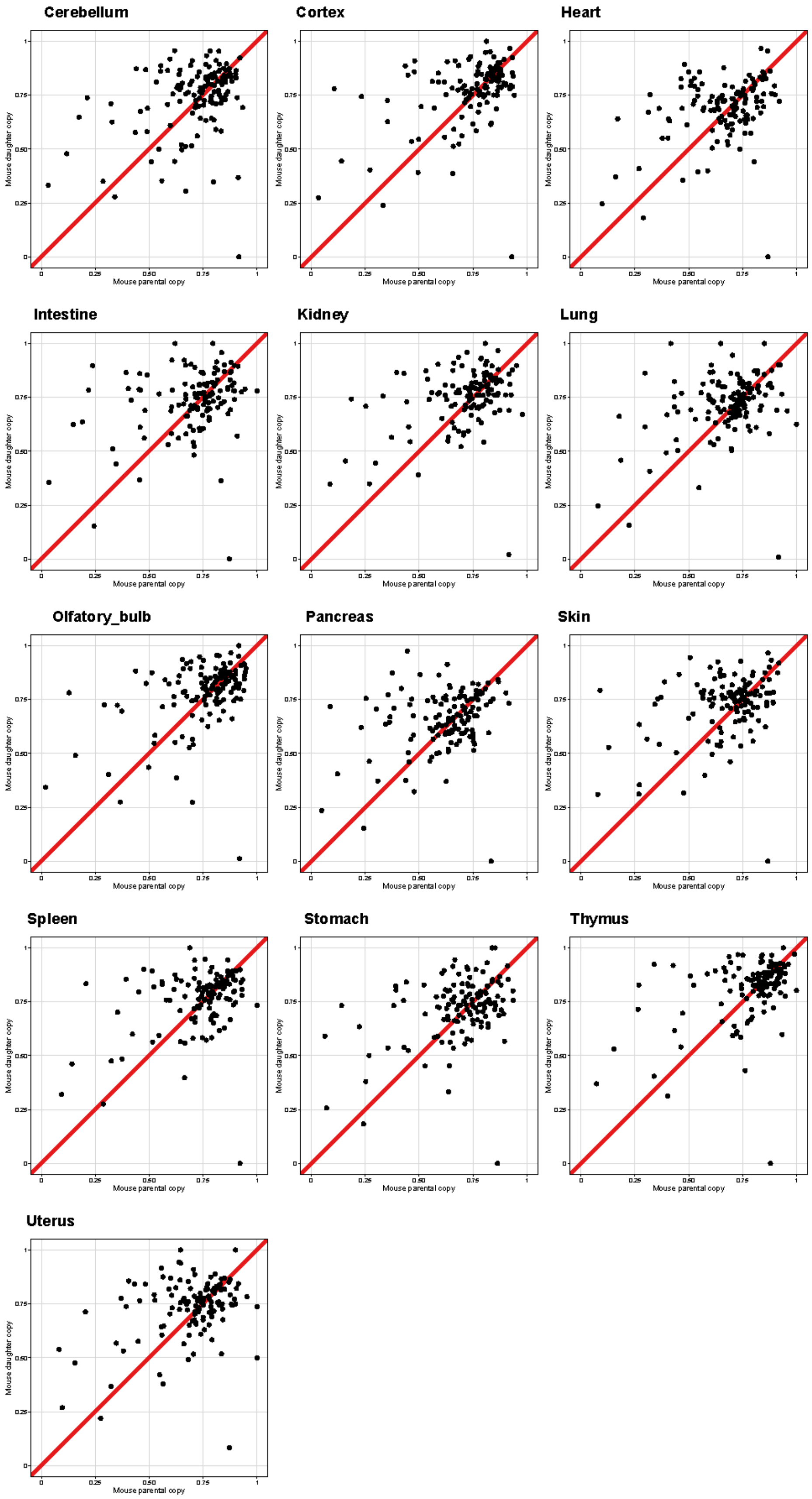

Each dot represents a pair of genes within a trio. For each graph, the red line corresponds to an equal amount of methylation in both genes.

5.8. Comparison of gene body methylation of mouse daughter vs. mouse parental copies without retrogenes: violin plots

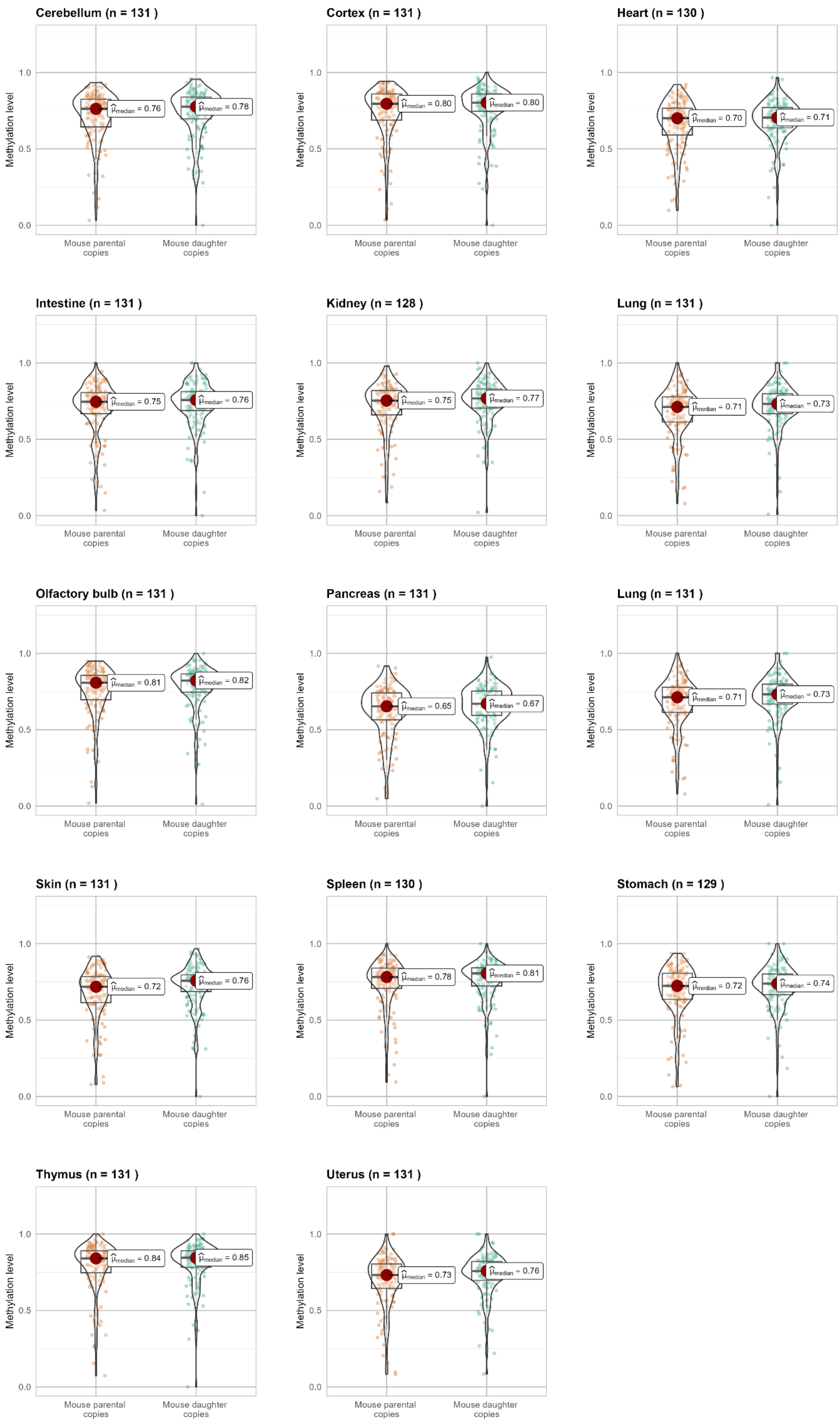

Supplement: msae259_Supplementary_Data [file msae259_supplementary_data.zip › DatasetS3.pdf]
