# Supplementary material for: Asymmetrical Evolution of Promoter Methylation of Mammalian Genes after Duplication
Source: Mol Biol Evol. 2024 Dec 17;41(12):msae259. doi: 10.1093/molbev/msae259 (PMC11683416; doi:10.1093/molbev/msae259)
Supplement: msae259_Supplementary_Data [file msae259_supplementary_data.zip › DatasetS4.pdf]

Supplementary Materials – Dataset S4: Expression and Observed/Expected CpG ratio analyses

CONTENTS

1. Correlation of Observed/Expected CpG ratio of recent duplicates with the Gene Order Conservation (GOC) scores 2

1.1. Human genes 2

1.2. Mouse genes 2

2. Correlation of mRNA expression levels of recent duplicates with the Gene Order Conservation (GOC) scores 3

2.1. Human genes 3

2.2. Mouse genes 5

1. Correlation of Observed/Expected CpG ratio of recent duplicates with the Gene Order Conservation (GOC) scores

1.1. Human genes

Below are the representations of the Observed/Expected CpG ratio ($CpG_{o/e}$) among many-to-one genes (duplicated in human, but not duplicated in mouse) with different Gene Order Conservation (GOC) scores. The horizontal lines indicate significant differences based on two-sided Dunn's pairwise tests with Holm correction for multiple comparisons. Comparisons were deemed significant if corrected p-values were lower than 0.05.

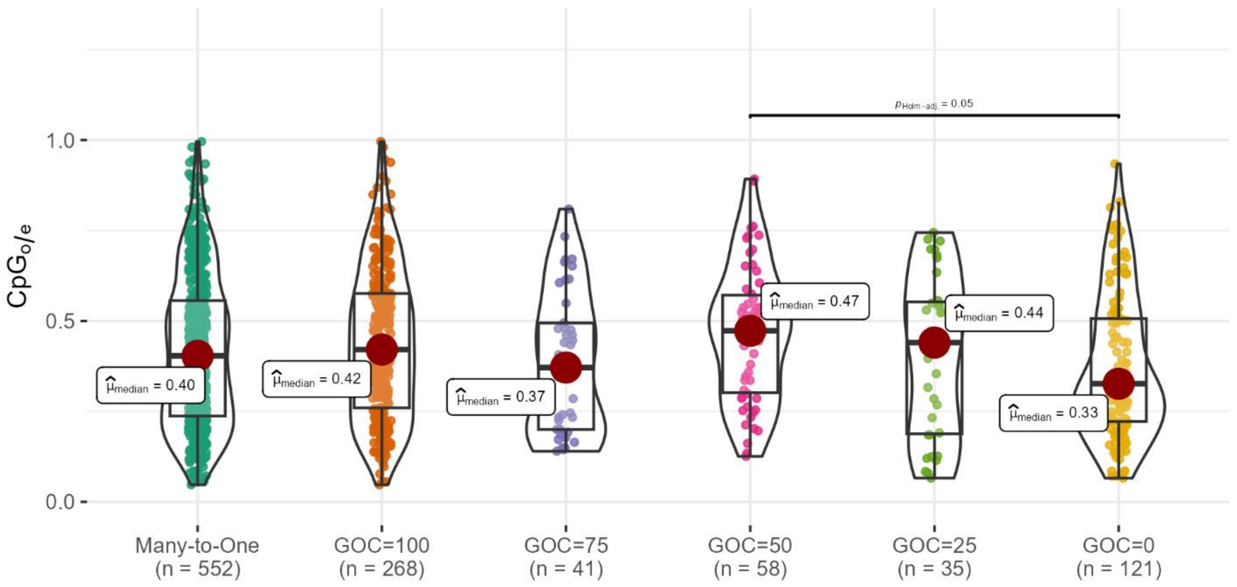

1.2. Mouse genes

Below are the representations of the Observed/Expected CpG ratio ($CpG_{o/e}$) among many-to-one genes (duplicated in mouse, but not duplicated in human) with different Gene Order Conservation (GOC) scores. The horizontal lines indicate significant differences based on two-sided Dunn's pairwise tests with Holm correction for multiple comparisons. Comparisons were deemed significant if corrected p-values were lower than 0.05.

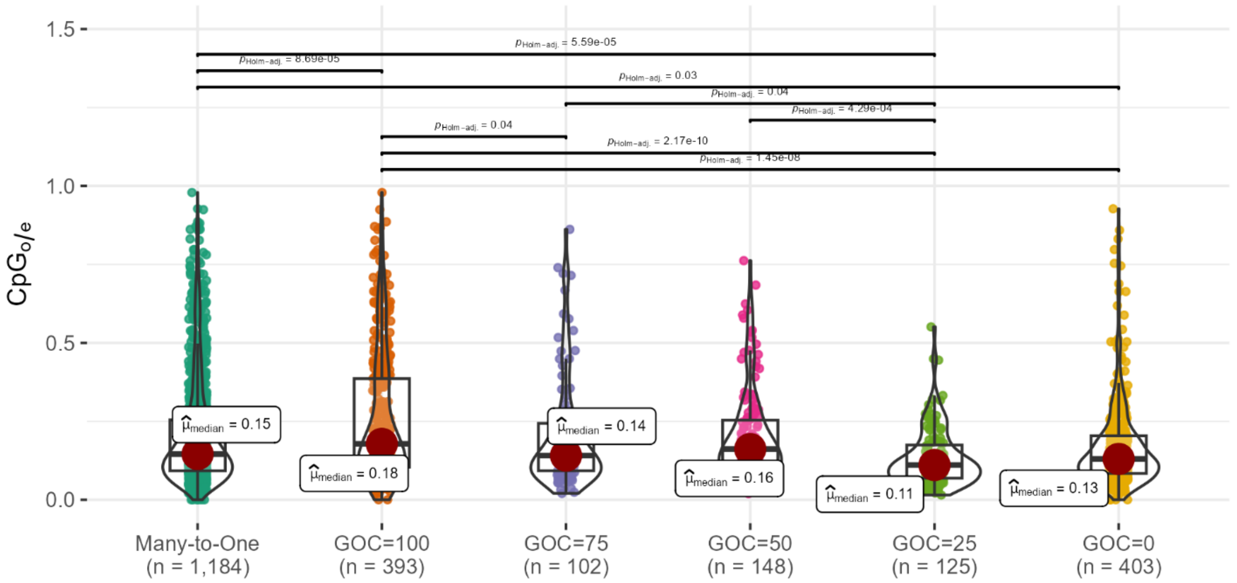

2. Correlation of mRNA expression levels of recent duplicates with the Gene Order Conservation (GOC) scores

2.1. Human genes

Below are the representations of the mRNA expression levels among many-to-one genes (duplicated in human, but not duplicated in mouse) with different Gene Order Conservation (GOC) scores, in the 5 human tissues analyzed. Expression values were log-transformed using $\log(x + 1)$, to handle zero values in the data. The horizontal lines indicate significant differences based on two-sided Dunn's pairwise tests with Holm correction for multiple comparisons. Comparisons were deemed significant if corrected p-values were lower than 0.05.

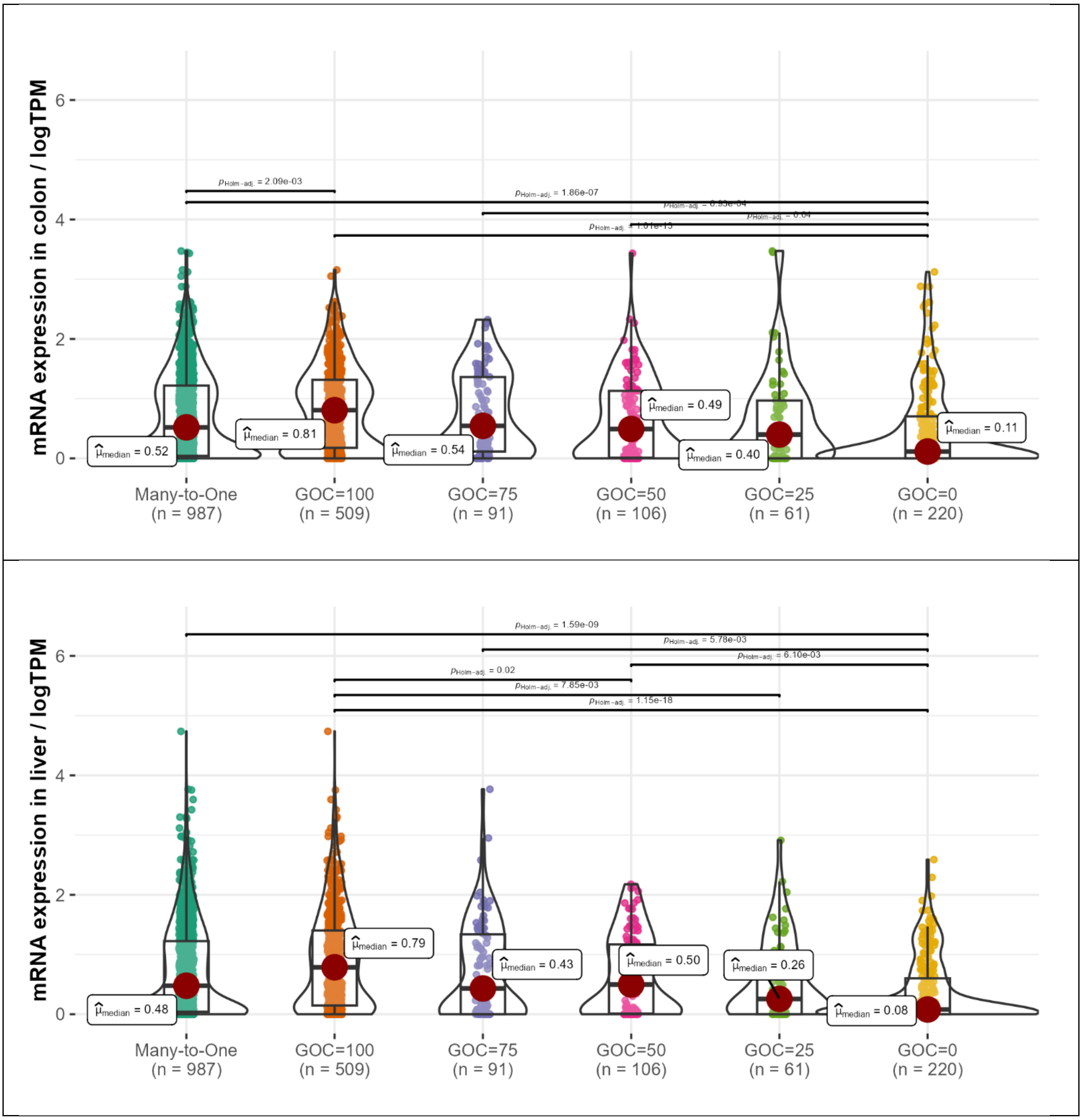

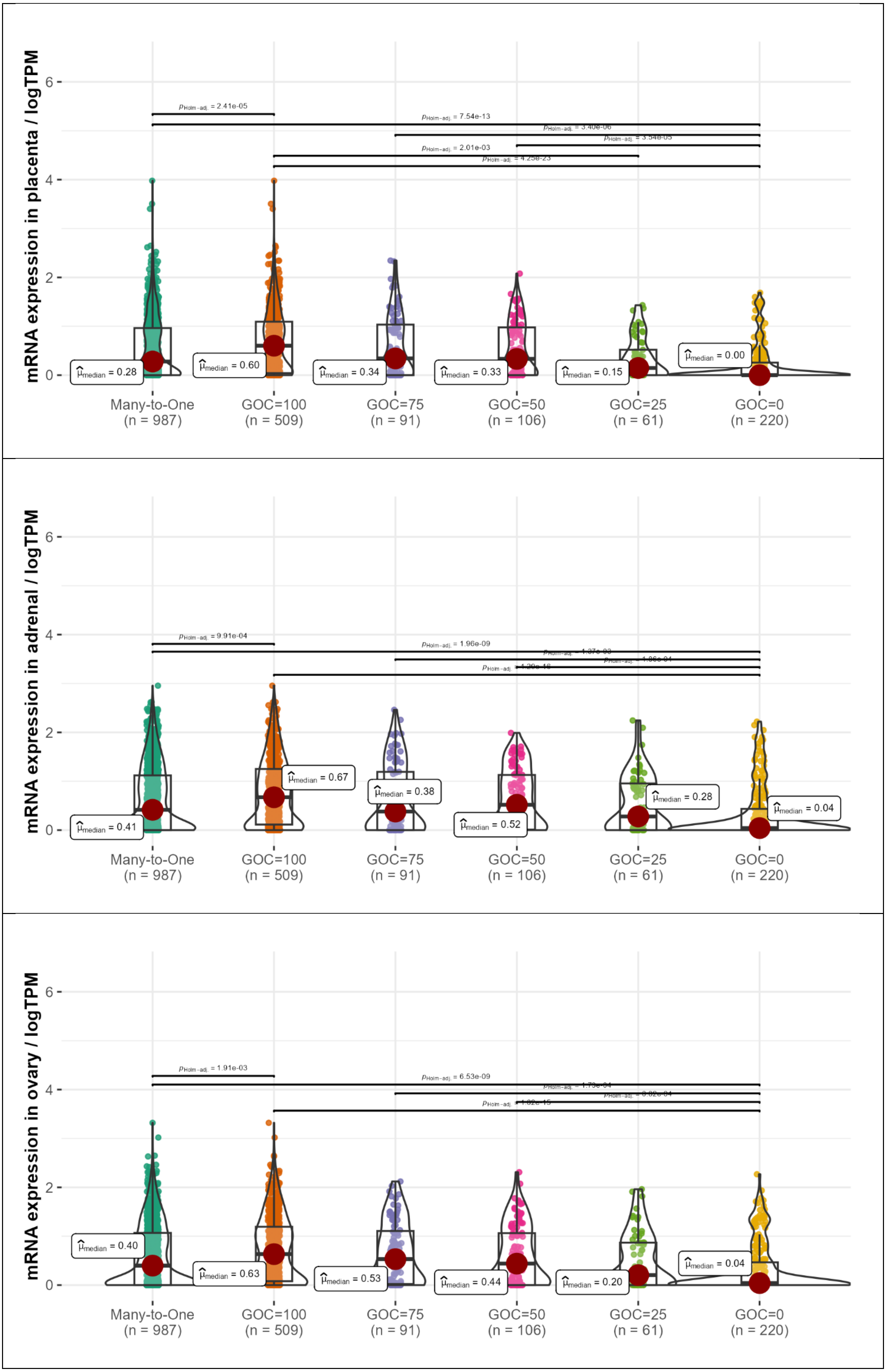

mRNA expression in placenta / logTPM
$p_{Holm-adj.}$ = 2.41e-05
$p_{Holm-adj.}$ = 7.54e-13
$p_{Holm-adj.}$ = 2.01e-03
$\hat{\mu}_{median}$ = 0.28
$\hat{\mu}_{median}$ = 0.60
$\hat{\mu}_{median}$ = 0.34
$\hat{\mu}_{median}$ = 0.33
$\hat{\mu}_{median}$ = 0.15
$\hat{\mu}_{median}$ = 0.00
Many-to-One (n = 987)
GOC=100 (n = 509)
GOC=75 (n = 91)
GOC=50 (n = 106)
GOC=25 (n = 61)
GOC=0 (n = 220)
mRNA expression in adrenal / logTPM
$p_{Holm-adj.}$ = 9.91e-04
$p_{Holm-adj.}$ = 1.96e-09
$\hat{\mu}_{median}$ = 0.41
$\hat{\mu}_{median}$ = 0.67
$\hat{\mu}_{median}$ = 0.38
$\hat{\mu}_{median}$ = 0.52
$\hat{\mu}_{median}$ = 0.28
$\hat{\mu}_{median}$ = 0.04
Many-to-One (n = 987)
GOC=100 (n = 509)
GOC=75 (n = 91)
GOC=50 (n = 106)
GOC=25 (n = 61)
GOC=0 (n = 220)
mRNA expression in ovary / logTPM
$p_{Holm-adj.}$ = 1.91e-03
$p_{Holm-adj.}$ = 6.53e-09
$\hat{\mu}_{median}$ = 0.40
$\hat{\mu}_{median}$ = 0.63
$\hat{\mu}_{median}$ = 0.53
$\hat{\mu}_{median}$ = 0.44
$\hat{\mu}_{median}$ = 0.20
$\hat{\mu}_{median}$ = 0.04
Many-to-One (n = 987)
GOC=100 (n = 509)
GOC=75 (n = 91)
GOC=50 (n = 106)
GOC=25 (n = 61)
GOC=0 (n = 220)

2.2. Mouse genes

Below are the representations of the mRNA expression levels among many-to-one genes (duplicated in mouse, but not duplicated in human) with different Gene Order Conservation (GOC) scores, in the 5 human tissues analyzed. Expression values were log-transformed using $\log(x + 1)$, to handle zero values in the data. The horizontal lines indicate significant differences based on two-sided Dunn's pairwise tests with Holm correction for multiple comparisons. Comparisons were deemed significant if corrected p-values were lower than 0.05.

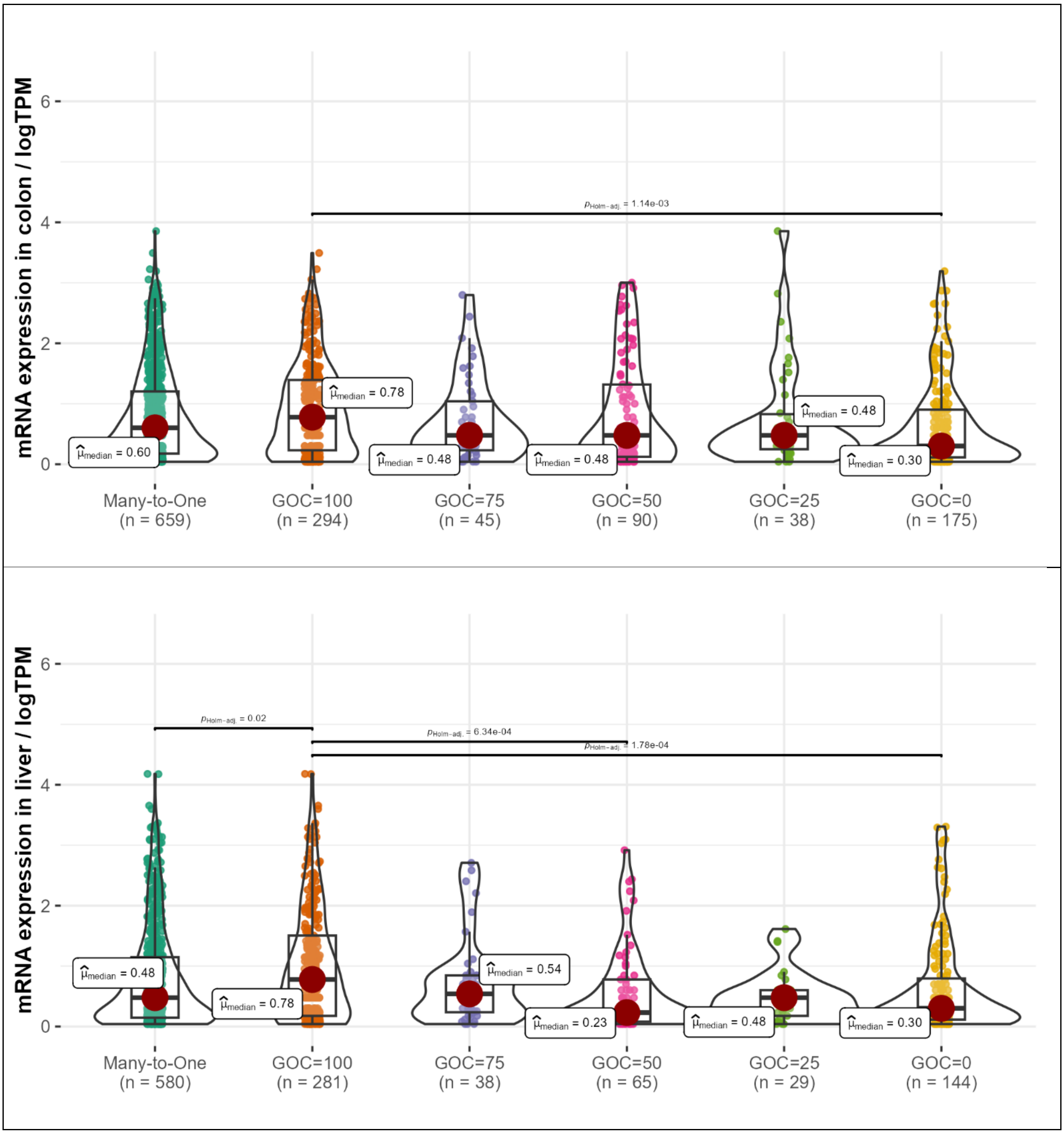

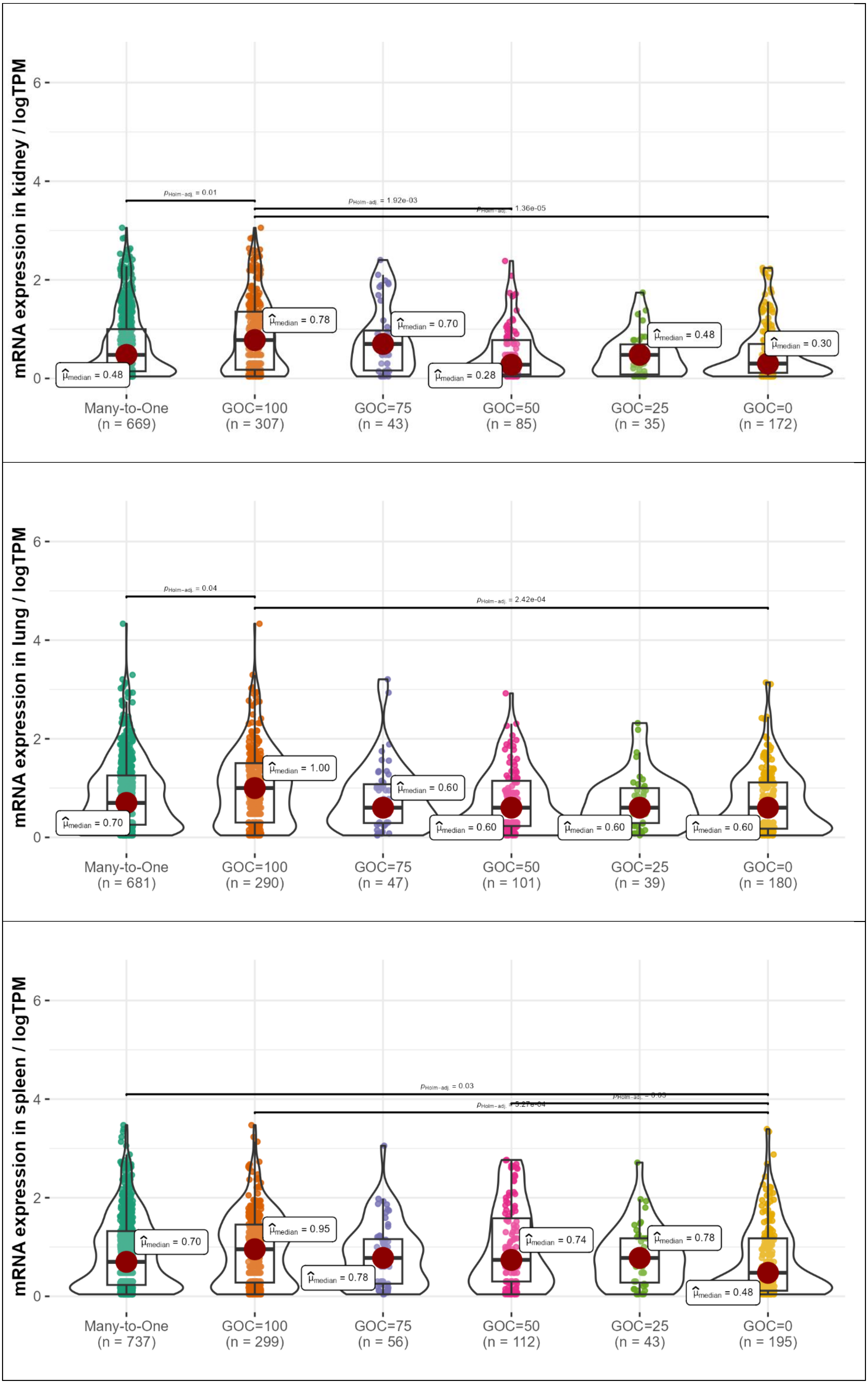

mRNA expression in kidney / logTPM
0
2
4
6
$p_{\text{Holm-adj.}}$ = 0.01
$p_{\text{Holm-adj.}}$ = 1.92e-03
$p_{\text{Holm-adj.}}$ = 1.36e-05
$\hat{\mu}_{\text{median}}$ = 0.48
$\hat{\mu}_{\text{median}}$ = 0.78
$\hat{\mu}_{\text{median}}$ = 0.70
$\hat{\mu}_{\text{median}}$ = 0.28
$\hat{\mu}_{\text{median}}$ = 0.48
$\hat{\mu}_{\text{median}}$ = 0.30
Many-to-One (n = 669)
GOC=100 (n = 307)
GOC=75 (n = 43)
GOC=50 (n = 85)
GOC=25 (n = 35)
GOC=0 (n = 172)
mRNA expression in lung / logTPM
0
2
4
6
$p_{\text{Holm-adj.}}$ = 0.04
$p_{\text{Holm-adj.}}$ = 2.42e-04
$\hat{\mu}_{\text{median}}$ = 0.70
$\hat{\mu}_{\text{median}}$ = 1.00
$\hat{\mu}_{\text{median}}$ = 0.60
$\hat{\mu}_{\text{median}}$ = 0.60
$\hat{\mu}_{\text{median}}$ = 0.60
$\hat{\mu}_{\text{median}}$ = 0.60
Many-to-One (n = 681)
GOC=100 (n = 290)
GOC=75 (n = 47)
GOC=50 (n = 101)
GOC=25 (n = 39)
GOC=0 (n = 180)
mRNA expression in spleen / logTPM
0
2
4
6
$p_{\text{Holm-adj.}}$ = 0.03
$p_{\text{Holm-adj.}}$ = 0.03
$p_{\text{Holm-adj.}}$ = 5.27e-04
$\hat{\mu}_{\text{median}}$ = 0.70
$\hat{\mu}_{\text{median}}$ = 0.95
$\hat{\mu}_{\text{median}}$ = 0.78
$\hat{\mu}_{\text{median}}$ = 0.74
$\hat{\mu}_{\text{median}}$ = 0.78
$\hat{\mu}_{\text{median}}$ = 0.48
Many-to-One (n = 737)
GOC=100 (n = 299)
GOC=75 (n = 56)
GOC=50 (n = 112)
GOC=25 (n = 43)
GOC=0 (n = 195)
