# Supplementary material for: Asymmetrical Evolution of Promoter Methylation of Mammalian Genes after Duplication
Source: Mol Biol Evol. 2024 Dec 17;41(12):msae259. doi: 10.1093/molbev/msae259 (PMC11683416; doi:10.1093/molbev/msae259)
Supplement: msae259_Supplementary_Data [file msae259_supplementary_data.zip › SupplementaryMaterial.pdf]

Supplementary Material

CONTENTS

This PDF file includes:

1. **Figure S1.** Description of the Gene Order Conservation (GOC) score from Ensembl.
2. **Table S1.** Spearman's correlations between levels of promoter or gene body methylation of recent duplicates and GOCs.
3. **Tables S2-S5.** Comparison of promoter methylation levels in trios (parental copy, daughter copy, unique ortholog). Results of binominal tests.
4. **Tables S6-S9.** Comparison of gene body methylation levels in trios (parental copy, daughter copy, unique ortholog). Results of binominal tests.
5. **Tables S10.** Comparison of observed/expected CpG ratio ($CpG_{o/e}$) in trios (parental copy, daughter copy, unique ortholog). Results of binominal tests.
6. **Table S11.** Spearman's correlations between mRNA abundances and GOCs.
7. **Tables S12-S13.** Comparison of mRNA expression levels in trios (parental copy, daughter copy, unique ortholog). Results of binominal tests.
8. **Table S14.** Spearman's correlations between levels of promoter methylation and levels of gene body methylation.

Other Supplementary Materials for this manuscript include the following:

- **Dataset S1.** Promoter methylation analyses.
- **Dataset S2.** UCSC Genome browser screenshots displaying DNA methylation data for specific genes.
- **Dataset S3.** Gene body methylation analyses.
- **Dataset S4.** Expression and Observed/Expected CpG ratio analyses.

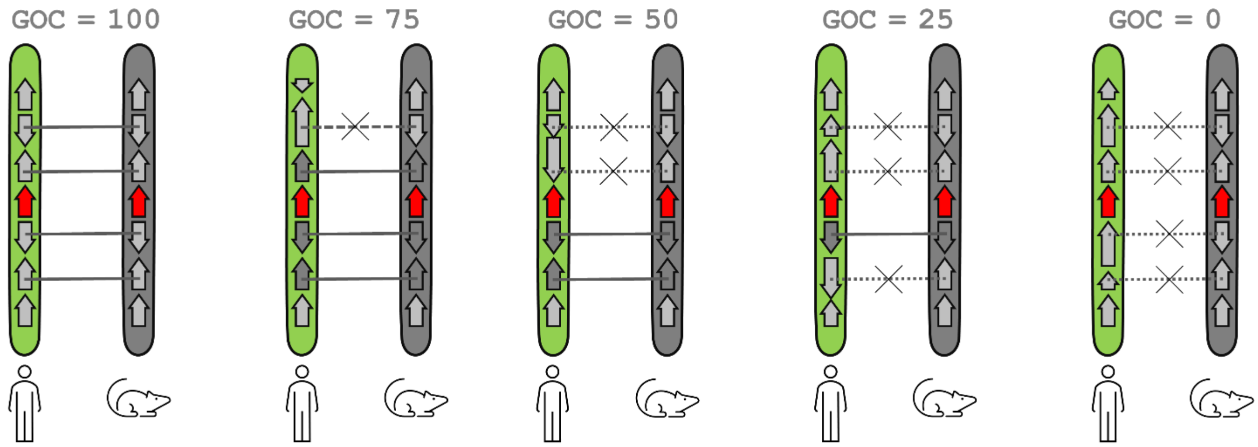

Figure S1. Description of the Gene Order Conservation (GOC) score from Ensembl.

Table S1: Spearman's correlations between levels of promoter methylation of recent duplicates and GOCs.

Organism	Tissue	*n*	ρ	p-value
Human	Adrenal gland	451	-0.225	1.31e-06*
	B cells	451	-0.224	1.48e-06*
	Colon	451	-0.226	1.26e-06*
	ESC	451	-0.210	7.01e-06*
	Hair	451	-0.232	6.09e-07*
	Liver	451	-0.243	1.77e-07*
	Neuron	451	-0.220	2.46e-06*
	Ovary	451	-0.230	7.56e-07*
	Placenta	451	-0.132	5.05e-03*
	Sperm	451	-0.123	8.91e-03*
Mouse	Cerebellum	1104	-0.123	4.13e-05*
	Colon	1094	-0.189	2.87e-10*
	Cortex	1131	-0.111	1.80e-04*
	Heart	1131	-0.079	8.11e-03*
	Intestine	1116	-0.173	5.51e-09*
	Kidney	1109	-0.138	4.28e-06*
	Liver	1131	-0.119	6.40e-05*
	Lung	1123	-0.103	5.80e-04*
	Olfactory bulb	1126	-0.126	2.13e-05*
	Pancreas	1112	-0.101	7.65e-04*
	Placenta	1122	-0.071	1.72e-02*
	Skin	1122	-0.142	1.71e-06*
	Spleen	1132	-0.113	1.45e-04*
	Stomach	1096	-0.142	2.38e-06*
	Thymus	1121	-0.130	1.26e-05*
	Uterus	1113	-0.159	1.01e-07*

*, p-value < 0.05.

Table S2 Comparison of promoter methylation levels in trios including two copies in human and one in mouse: parental copy vs. daughter copy.

Gene set	Tissue	Trios with hypermethylated daughter copies	Total trios	p-value
**Including retrogenes**				
	Adrenal gland	33	50	1.64e-02*
	B cells	30	50	1.01e-01
	Colon	32	50	3.25e-02*
	ESC	37	50	4.68e-04*
	Hair	31	50	5.95e-02
	Liver	33	50	1.64e-02*
	Neuron	33	50	1.64e-02*
	Ovary	34	50	7.67e-03*
	Placenta	33	50	1.64e-02*
	Sperm	32	50	3.25e-02*
**Without retrogenes**				
	Adrenal gland	19	36	4.34e-01
	B cells	18	36	5.66e-01
	Colon	19	36	4.34e-01
	ESC	24	36	3.26e-02*
	Hair	19	36	4.34e-01
	Liver	20	36	3.09e-01
	Neuron	20	36	3.09e-01
	Ovary	20	36	3.09e-01
	Placenta	20	36	3.09e-01
	Sperm	19	36	4.34e-01

ESC, embryonic stem cells. *, p-value < 0.05 (one-tailed binomial test).

Table S3: Comparison of promoter methylation levels in trios including two copies in human and one in mouse: human parental copy or human daughter copy vs. unique mouse ortholog.

Gene set	Tissue	Hypermethylated human gene (parental or daughter) vs. mouse gene	Total trios	p-value
**Including retrogenes**				
Human parental vs. mouse	Placenta	45	64	7.81e-04*
	Colon	36	64	1.91e-01
	Liver	38	64	8.43e-02
Human daughter vs. mouse	Placenta	43	55	1.65e-05*
	Colon	37	55	7.23e-03*
	Liver	39	55	1.33e-03*
**Without retrogenes**				
Human parental vs. mouse	Placenta	33	50	1.64e-02*
	Colon	26	50	4.44e-01
	Liver	30	50	1.01e-01
Human daughter vs. mouse	Placenta	29	41	5.75e-03*
	Colon	23	41	2.66e-01
	Liver	25	41	1.06e-01

*, p-value < 0.05 (one-tailed binomial test).

Table S4: Comparison of promoter methylation levels in trios including two copies in mouse and one in human: parental copy vs. daughter copy.

Gene set	Tissue	Trios with hypermethylated daughter copies	Total trios	p-value
**Including retrogenes**				
	Cerebellum	80	136	2.41e-02*
	Colon	86	135	9.19e-04*
	Cortex	84	142	1.78e-02*
	Heart	85	141	9.03e-03*
	Intestine	91	137	7.52e-05*
	Kidney	89	136	1.99e-04*
	Liver	86	141	5.63e-03*
	Lung	93	139	4.14e-05*
	Olfactory bulb	84	138	6.64e-03*
	Pancreas	81	136	1.58e-02*
	Placenta	86	139	3.22e-03*
	Skin	83	140	1.71e-02*
	Spleen	81	141	4.59e-02*
	Stomach	96	135	5.03e-07*
	Thymus	82	139	2.07e-02*
	Uterus	82	139	2.07e-02*
**Without retrogenes**				
	Cerebellum	70	126	1.23e-01
	Colon	77	125	5.98e-03*
	Cortex	74	132	9.57e-02
	Heart	76	131	4.01e-02*
	Intestine	81	127	1.21e-03*
	Kidney	80	126	1.56e-03*
	Liver	77	131	2.71e-02*
	Lung	83	129	7.15e-04*
	Olfactory bulb	74	128	4.63e-02*
	Pancreas	72	126	6.48e-02
	Placenta	77	129	1.71e-02*
	Skin	74	130	6.78e-02
	Spleen	72	131	1.47e-01
	Stomach	86	125	1.59e-05*
	Thymus	73	129	7.93e-02
	Uterus	72	129	1.09e-01

*, p-value < 0.05 (one-tailed binomial test).

Table S5: Comparison of promoter methylation levels in trios including two copies in mouse and one in human: mouse parental copy or mouse daughter copy vs. unique human ortholog.

Gene set	Tissue	Hypermethylated mouse gene (parental or daughter) vs. human gene	Total trios	p-value
**Including retrogenes**				
Mouse parental vs. human	Placenta	61	137	9.14e-01
	Colon	74	135	1.51e-01
	Liver	75	138	1.75e-01
Mouse daughter vs. human	Placenta	73	136	2.20e-01
	Colon	89	135	1.35e-04*
	Liver	88	137	5.46e-04*
**Without retrogenes**				
Mouse parental vs. human	Placenta	52	124	9.71e-01
	Colon	64	122	3.25e-01
	Liver	66	125	2.96e-01
Mouse daughter vs. human	Placenta	61	124	6.06e-01
	Colon	77	123	3.30e-03*
	Liver	76	125	9.84e-03*

*, p-value < 0.05 (one-tailed binomial test).

Table S6: Comparison of gene body methylation levels in trios including two copies in human and one in mouse: parental copy vs. daughter copy.

Gene set	Tissue	Trios with hypermethylated daughter copies	Total trios	p-value
**Including retrogenes**				
	Adrenal gland	37	63	1.04e-01
	B cells	40	63	2.15e-02*
	Colon	41	63	1.13e-02*
	ESC	38	63	6.50e-02
	Hair	38	62	4.90e-02*
	Liver	41	63	1.13e-02*
	Neuron	36	63	1.57e-01
	Ovary	39	63	3.85e-02*
	Placenta	35	63	2.25e-01
	Sperm	29	63	7.75e-01
**Without retrogenes**				
	Adrenal gland	22	48	7.65e-01
	B cells	25	48	4.43e-01
	Colon	27	48	2.35e-01
	ESC	23	48	6.67e-01
	Hair	25	47	3.85e-01
	Liver	27	48	2.35e-01
	Neuron	22	48	7.65e-01
	Ovary	25	48	4.43e-01
	Placenta	23	48	6.67e-01
	Sperm	22	48	7.65e-01

ESC, embryonic stem cells. *, p-value < 0.05 (one-tailed binomial test).

Table S7: Comparison of gene body methylation levels in trios including two copies in human and one in mouse: human parental copy or human daughter copy vs. unique mouse ortholog.

Gene set	Tissue	Hypermethylated human gene (parental or daughter) vs. mouse gene	Total trios	p-value
**Including retrogenes**				
Human parental vs. mouse	Placenta	55	68	1.37e-07*
	Colon	43	68	1.92e-02*
	Liver	43	68	1.92e-02*
Human daughter vs. mouse	Placenta	53	68	2.06e-06*
	Colon	47	68	1.09e-03*
	Liver	45	68	5.17e-03*
**Without retrogenes**				
Human parental vs. mouse	Placenta	41	53	4.09e-05*
	Colon	30	53	2.05e-01
	Liver	34	53	2.67e-02*
Human daughter vs. mouse	Placenta	40	53	1.34e-04*
	Colon	32	53	8.45e-02
	Liver	32	53	8.45e-02

*, p-value < 0.05 (one-tailed binomial test).

Table S8: Comparison of gene body methylation levels in trios including two copies in mouse and one in human: parental copy vs. daughter copy.

Gene set	Tissue	Trios with hypermethylated daughter copies	Total trios	p-value
**Including retrogenes**				
	Cerebellum	82	143	4.70e-02*
	Colon	75	140	2.23e-01
	Cortex	84	142	1.78e-02*
	Heart	74	141	3.07e-01
	Intestine	78	142	1.38e-01
	Kidney	79	139	6.33e-02
	Liver	74	142	3.37e-01
	Lung	82	142	3.88e-02*
	Olfactory bulb	81	142	5.53e-02
	Pancreas	77	142	1.78e-01
	Placenta	72	142	4.67e-01
	Skin	88	142	2.72e-03*
	Spleen	81	141	4.59e-02*
	Stomach	78	140	1.02e-01
	Thymus	74	142	3.37e-01
	Uterus	84	142	1.78e-02*
**Without retrogenes**				
	Cerebellum	72	131	1.47e-01
	Colon	66	129	4.30e-01
	Cortex	74	131	8.09e-02
	Heart	65	130	5.35e-01
	Intestine	67	131	4.31e-01
	Kidney	70	128	1.65e-01
	Liver	66	131	5.00e-01
	Lung	74	131	8.09e-02
	Olfactory bulb	71	131	1.91e-01
	Pancreas	68	131	3.63e-01
	Placenta	64	131	6.37e-01
	Skin	78	131	1.78e-02*
	Spleen	73	130	9.41e-02
	Stomach	70	129	1.89e-01
	Thymus	64	131	6.37e-01
	Uterus	73	131	1.11e-01

*, p-value < 0.05 (one-tailed binomial test).

Table S9: Comparison of gene body methylation levels in trios including two copies in mouse and one in human: mouse parental copy or mouse daughter copy vs. unique human ortholog.

Gene set	Tissue	Hypermethylated mouse gene (parental or daughter) vs. human gene	Total trios	p-value
**Including retrogenes**				
Mouse parental vs. human	Placenta	63	145	9.52e-01
	Colon	91	145	1.33e-03*
	Liver	84	145	3.37e-02*
Mouse daughter vs. human	Placenta	59	143	9.85e-01
	Colon	106	141	8.47e-10*
	Liver	85	143	1.47e-02*
**Without retrogenes**				
Mouse parental vs. human	Placenta	55	132	9.78e-01
	Colon	81	132	5.66e-03*
	Liver	75	132	6.93e-02
Mouse daughter vs. human	Placenta	51	130	9.95e-01
	Colon	94	128	5.44e-08*
	Liver	73	130	9.41e-02

*, p-value < 0.05 (one-tailed binomial test).

Table S10: Comparison of observed/expected CpG ratio ($CpG_{o/e}$) in trios (parental copy, daughter copy, unique ortholog).

Gene set	Trios with less $CpG_{o/e}$ in promoter of daughter copies	Total trios	p-value
**Including retrogenes***97			
Two copies in human and one in mouse	33	52	3.52e-02*
Two copies in mouse and one in human	97	148	9.72e-05*
**Without retrogenes**			
Two copies in human and one in mouse	20	38	4.36e-01
Two copies in mouse and one in human	85	135	1.64e-03*

*, p-value < 0.05 (one-tailed binomial test).

Table S11: Spearman's correlations between mRNA abundances and GOCs.

Organism	Tissue	*n*	ρ	p-value
Human	Adrenal gland	987	0.262	6.53e-17*
	Colon	987	0.247	3.65e-15*
	Liver	987	0.293	4.85e-21*
	Ovary	987	0.255	4.35e-16*
	Placenta	987	0.317	1.61e-24*
Mouse	Colon	642	0.153	9.59e-05*
	Kidney	642	0.215	3.60e-08*
	Liver	557	0.214	3.28e-07*
	Lung	657	0.178	4.49e-06*
	Spleen	705	0.144	1.19e-04*

*, p-value < 0.05

Table S12: Comparison of mRNA abundances in trios including two copies in human and one in mouse: trios with underexpressed daughter copies.

Gene set	Tissue	Trios with underexpressed daughter copies	Total trios	p-value
**Including retrogenes**				
	Placenta	33	44	6.30e-04*
	Colon	35	47	5.44e-04*
	Liver	34	46	8.21e-04*
	Adrenal	34	45	4.12e-04*
	Ovary	35	43	2.10e-05*
**Without retrogenes**				
	Placenta	20	30	4.94e-02*
	Colon	21	33	8.14e-02
	Liver	20	32	1.08e-01
	Adrenal	21	31	3.54e-02*
	Ovary	21	29	1.21e-02*

*, p-value < 0.05 (one-tailed binomial test).

Table S13: Comparison of mRNA abundances in trios including two copies in mouse and one in human: trios with underexpressed daughter copies.

Gene set	Tissue	Trios with underexpressed daughter copies	Total trios	p-value
**Including retrogenes**				
	Colon	31	45	8.05e-03*
	Kidney	32	46	5.68e-03*
	Liver	23	33	1.75e-02*
	Lung	35	51	5.49e-03*
	Spleen	32	47	9.31e-03*
**Without retrogenes**				
	Colon	28	42	2.18e-02*
	Kidney	29	43	1.58e-02*
	Liver	21	31	3.54e-02*
	Lung	33	48	6.64e-03*
	Spleen	27	42	4.42e-02*

*, p-value < 0.05 (one-tailed one-tailed binomial test).

Table S14: Spearman's correlations between levels of promoter methylation and levels of gene body methylation

Organism	Tissue	*n*	ρ	p-value
Human	Adrenal gland	18417	0.335	$<10^{-300}$*
	B-cells	18417	0.354	$<10^{-300}$*
	Colon	18417	0.268	1.93×10^{-301}*
	ESC	18417	0.489	$<10^{-300}$*
	Hair	18417	0.376	$<10^{-300}$*
	Liver	18417	0.334	$<10^{-300}$*
	Neuron	18417	0.541	$<10^{-300}$*
	Ovary	18417	0.346	$<10^{-300}$*
	Placenta	18417	0.215	1.80×10^{-191}*
	Sperm	18417	0.428	$<10^{-300}$*
Mouse	Cerebellum	19242	0.400	$<10^{-300}$*
	Colon	19155	0.396	$<10^{-300}$*
	Cortex	19343	0.491	$<10^{-300}$*
	Heart	19343	0.248	6.62×10^{-270}*
	Intestine	19268	0.343	$<10^{-300}$*
	Kidney	19229	0.369	$<10^{-300}$*
	Liver	19295	0.254	7.55×10^{-282}*
	Lung	19302	0.302	$<10^{-300}$*
	Olfactory bulb	19337	0.487	$<10^{-300}$*
	Pancreas	19259	0.282	$<10^{-300}$*
	Placenta	19314	0.033	3.72×10^{-6}*
	Skin	19296	0.387	$<10^{-300}$*
	Spleen	19330	0.344	$<10^{-300}$*
	Stomach	19178	0.352	$<10^{-300}$*
	Thymus	19290	0.434	$<10^{-300}$*
	Uterus	19240	0.335	$<10^{-300}$*

*, p-value < 0.05
